# Preparing Medical Students to Be Physician Leaders: A Leadership Training Program for Students Designed and Led by Students

**DOI:** 10.15766/mep_2374-8265.10863

**Published:** 2019-12-13

**Authors:** Kristen Richard, Michael Noujaim, Luanne E. Thorndyke, Melissa A. Fischer

**Affiliations:** 1 Resident, Department of Pediatrics, University of Massachusetts Medical School; 2 Resident, Department of Internal Medicine, Duke University School of Medicine; 3 Vice Provost for Faculty Affairs, University of Massachusetts Medical School; 4 Professor of Medicine, University of Massachusetts Medical School; 5 Associate Dean for Undergraduate Medical Education, University of Massachusetts Medical School; 6 Associate Dean for Curriculum Innovation & iCELS, University of Massachusetts Medical School

**Keywords:** Leadership, Teamwork, Leadership Training, Program Evaluation

## Abstract

**Introduction:**

Leadership is an area of education and training that is critical to the development of medical providers as health care professionals, yet few medical school curricula offer formal training in this area.

**Methods:**

We designed and implemented a course to develop and enhance the leadership and teamwork skills of first-year medical students to better prepare them for medical practice. Following a systematic literature review to identify leadership core competencies, the Leadership in Medicine Optional Enrichment Elective (OEE) was developed in accordance with the University of Massachusetts Medical School's course guidelines. The elective included six interactive sessions to advance skills in the areas of recognizing and utilizing effective leadership styles, communication within the health care team, giving and receiving feedback, delegating responsibilities, and direction setting. We designed a robust, evidence-based, scholarly evaluation plan for the OEE that was integral to ongoing quality improvement of the course.

**Results:**

Outcomes were assessed in alignment with the Kirkpatrick method of standardized evaluation. A total of 26 participants completed the course. At completion, participants demonstrated learning and advancement of skills in all five leadership domains. Furthermore, participants found meaning in the course and planned to utilize their skills in future medical practice.

**Discussion:**

The development, implementation, and evaluation of this program can serve as a model for future course development, and the program can be adapted and implemented by other institutions in an effort to address the learning gap regarding leadership education.

## Educational Objectives

By the end of this activity, learners will be able to:

1. Identify their personal style of leadership, recognizing strengths and weaknesses and how to accommodate both.
2. Utilize effective communication strategies specific to the appropriate learning environment.
3. Appropriately delegate team member responsibilities while practicing effective team communication.
4. Provide constructive feedback to help improve team function.
5. Create a team project with a clearly defined mission and vision.

## Introduction

Medical knowledge alone is insufficient to provide excellent medical care. The role of a physician goes beyond simply providing clinical care and encompasses a leadership role within the health care team, medical center, profession, and community. Clinicians must master the necessary skills to cooperate and collaborate with other health care professionals and effectively lead a team in the patient-care environment and in the larger context of health care systems. However, no standard curricular or accreditation requirement currently exists for US medical schools to provide leadership development for students. Exposure to these skills is achieved primarily through role modeling and passive observation of peers and mentors, which may provide subjective and variable experiences.^1^ Nevertheless, many medical schools recognize the necessity of leadership training,^2^ as evidenced by the increasing number of US medical programs that are just beginning to incorporate some form of leadership training into their curricula, although experiences remain rare and are inconsistent. In 2008, the UK's National Health Service developed the Medical Leadership Competency Framework (MLCF) to direct leadership training for physicians and medical students on the national level.^3,4^ In the US, surveys have shown that students strongly support the integration of these skills within the medical school curriculum.^5^ However, although medical students acknowledge the need for leadership education, there is currently no standard requirement to provide this training.

Barriers to standardized training include cost of implementation, staff training, and available schedule time. Additionally, evaluating instated leadership curricula adds additional time and potential cost.^6^ Given this new area of research, no single systematic review of current training curricula existed prior to the development of this course. As a part of our program development, a senior student conducted a systematic search of the PubMed and Education Resources Information Center databases and identified 11 programs with piloted educational assessments. Analysis of the information revealed that the most common learning method utilized by these programs was simulation-based scenarios,^7^ which unfortunately can be logistically and financially challenging to standardize. Our literature review was corroborated by an updated 2018 systematic review of leadership training for medical students, which found that, although overall positive, there is lack of consistent objective measures of effectiveness among training programs.^8^ As a result, we, who at the time were students at the University of Massachusetts Medical School (UMMS), successfully created a course intended to enhance the leadership and teamwork skills of medical students to prepare them for future experiences on the medical wards and in medical practice. Five leadership competencies were adapted from the five domains of the MLCF: leadership style, communication within the health care team, giving and receiving feedback, delegating responsibilities, and setting direction. Interactive sessions facilitated by faculty with special expertise in the associated area of development incorporated a multitude of educational strategies, including PowerPoint presentation, group discussion, team-based exercises, and reflective writing. The course was designed, implemented, and evaluated using a competency-based framework, and results showed that students increased their leadership skills and found the course relevant and worthwhile.

Although a few courses to teach leadership skills to trainees exist in the literature, no courses designed and implemented by students presently exist as a framework for a medical student leadership curriculum.^9,10^ A recent *MedEdPORTAL* publication presents a student-led program to teach leadership skills; however, that initiative is project and client focused, and it is neither intended to provide a comprehensive overview of leadership nor based on established leadership competencies.^11^ Therefore, our teaching module is novel in that it was designed and implemented by medical students, making it affordable and not time or labor intensive for academic administration, and can easily be adapted for other student groups. We targeted first-year medical students, as early training encourages participants to seek out leadership positions available to senior medical students that they may have not otherwise sought and reinforces skills utilization. We present our model as an example that another student or faculty administration for medical, nursing, or other graduate-level courses can easily adapt to fit individualized goals and objectives and can easily and efficiently implement at other institutions.

## Methods

### Course Development

UMMS provides opportunities for additional learning through Optional Enrichment Electives (OEEs), elective courses offered in various disciplines to enhance education in addition to that required in the standard curriculum. These courses are designed by students according to established criteria, overseen by faculty, and approved by the Longitudinal Curriculum Committee, a subcommittee of the Education Policy Committee. Under the guidance of the associate dean of undergraduate medical education (UME), we met regularly during the 2015–2016 academic year to develop the curriculum for six teaching sessions based on five competencies adapted from the five domains of the MLCF: leadership style, communication within the health care team, giving and receiving feedback, delegating responsibilities, and setting direction.^3^ A faculty review board approved the curriculum in accordance with OEE proposal and approval guidelines.

### Course Content

We created six relevant 90-minute interactive sessions. Each session consisted of a brief presentation by UMMS faculty with content knowledge and experience in the topic area (Appendices A–F), an interactive exercise to apply the learning (Appendices G–L), and student-led discussion to consolidate learning, as well as requiring students to complete a reflective writing application (Appendices N–R). We recruited faculty according to expertise and dedication to medical education. Qualification for faculty recruitment included the following:

• Recognized by students as a role model in leadership and teaching.
• Displayed an academic interest and/or publication(s) in the topic area.
• Obtained a leadership position within the medical school (assistant professor or professor) or a senior clinical leadership position within the hospital administration.

The road map of each session was adapted to teach the five aforementioned competencies. Course developers, in conjunction with faculty leaders, created introductory teaching PowerPoint presentations for each session to address the following topics:

• Session 1: recognizing and adapting effective leadership characteristics.
• Session 2: recognizing the value of interdisciplinary team communication.
• Session 3: incorporating delegation tools and techniques into leadership practice.
• Session 4: practicing effective constructive feedback.
• Session 5: constructing an appropriate vision and setting a direction for how to achieve it.
• Session 6: review.

We designed the sixth session as an optional review session to consolidate learning, in the form of a review activity. We have provided our resources for this optional session as a model that other course administrators may adapt (Appendix F). Multiple methods stimulated learner engagement, including individual reflection, small- and large-group discussion, small-group skill-building activities, and video technology. We followed the in-depth course outline, including reference to faculty facilitator qualifications, session-specific goals and objectives, and session materials, with referral to the appropriate appendix material for implementation, as noted in Table 1. In addition, we followed the instructions note in Appendices T–V for optional additional supplemental teaching activities for sessions 2, 3, and 4, respectively.

**Table 1.** Course Outline

Session	Faculty Leaders	Goals and Objectives	Outline	Materials	Evaluation
1. Leadership Styles: You as a Leader	UMass Memorial president/chief executive officer and vice president of operations Facilitator qualifications: • Recognized as a role model for leadership • Holds some type of leadership management position	Goals: • Discuss styles of leadership • Identify traits for successful leadership • Identify personal leadership style and recognize strengths and weaknesses, how to accommodate Objectives: • Identify individual leadership style based on article evaluation • Address pros and cons of your leadership style using group discussion	• 5:00–5:15: introduction^a^ • 5:15–6:00: PowerPoint lecture^b^ (Appendix A; note that this may include reading and discussion of optional supplemental material as an additional teaching activity) • 6:00–6:20: activity^c^ (style scenarios, Appendix G) • 6:20–6:30: wrap-up^d^	• PowerPoint, internet access, projector • Activity: clock • Evaluation: online survey software	• Students completed the precourse questionnaire (Appendix M) prior to the start of the course • Students completed the session questionnaire (Appendix N) after the session
2. Practicing Effective Communication	University of Massachusetts vice provost for faculty affairs and professor of medicine Facilitator qualification: • Holds some type of leadership management position	Goals: • Recognize how varied experiences of team members contribute to accomplish a shared goal • Learn to communicate effectively with various team members/roles Objective: • Use the technique of a PACE Palette activity to identify areas of strengths and weaknesses of communication	• 5:00–5:15: introduction^a^ • 5:15–5:30: PowerPoint lecture^b^ (Appendix B) • 5:30–6:20: activity^c^ (teamwork, Appendix H; note that this may include participation in optional supplemental activity [PACE Palette, Appendix T] as an alternative activity) • 6:20–6:30: wrap-up^d^	• PowerPoint, internet access, projector • Activity: one deck of cards, scissors • Optional activity: PACE Palette set (Appendix T) • Evaluation: online survey software	• Students completed the session questionnaire (Appendix O) after the session
3. Delegating Responsibilities	Assistant professor and instructor of multiple clinical skills courses for medical students Facilitator qualification: • Holds some type of leadership management position	Goal: • Learn to appropriately delegate responsibilities, practicing effective team communication Objective: • Use the technique of a skill-building exercise coupled with an educational video to develop skills of effective team communication, delegating responsibilities, and knowing one's limitations	• 5:00–5:15: introduction^a^ • 5:15–5:30: PowerPoint lecture^b^ (Appendix C) • 5:30–6:20: activity^c^ (practice delegation, Appendix I; note that this may include viewing of optional supplemental Advanced Cardiovascular Life Support video on team dynamics [Appendix U] as an additional teaching activity) • 6:20–6:30: wrap-up^d^	• PowerPoint, internet access, projector • Activity: three sets of children's building blocks (10 blocks per set) • Optional activity: internet access, projector • Evaluation: online survey software	• Students completed the session questionnaire (Appendix P) after the session
4. Giving and Receiving Feedback	Assistant professor and pediatric clerkship director Facilitator qualification: • Holds some type of leadership position as a team instructor	Goal: • Discuss components of effective feedback and become comfortable giving constructive criticism Objective: • Use the technique of a skill-building activity and an educational video to learn to incorporate feedback into regular interaction	• 5:00–5:15: introduction^a^ • 5:15–5:40: PowerPoint lecture^b^ (Appendix D; note that this may include viewing of optional supplemental video on feedback [Appendix V] as an additional teaching activity) • 5:40–6:20: activity^c^ (feedback figure, Appendix J) • 6:20–6:30: wrap-up^d^	• PowerPoint, internet access, projector • Activity: paper, pencil • Optional activity: internet access, projector • Evaluation: online survey software	• Students completed the session questionnaire (Appendix Q) after the session
5. Setting Direction: Vision, Mission, and Goals of a Team	Dean of the Graduate School of Nursing Facilitator qualification: • Holds some type of leadership position as a team instructor	Goal: • Conceptualize self as a leader in medicine and construct a vision of future career Objective: • Use the technique of a teamwork task to create personal vision/mission statement	• 5:00–5:15: introduction^a^ • 5:15–5:30: PowerPoint lecture^b^ (Appendix E) • 5:30–6:20: activity^c^ (mission statement, Appendix K) • 6:20–6:30: wrap-up^d^	• PowerPoint, internet, projector • Activity: paper, pencil • Evaluation: online survey software	• Students completed the session questionnaire (Appendix R) after the session
6. Consolidation: Bringing It All Together and Reflection (optional)	Professor and associate dean for undergraduate medical education, associate dean for curriculum innovation and interprofessional Center for Experiential Learning and Simulation Facilitator qualification: • Anyone recognized for leadership ability	Goal: • Review materials from previous sessions Objective: • Use a game to review takeaway points	• 5:00–5:15: introduction^a^ • 5:15–6:00: activity^c^ (consolidation game, Appendix L) • 6:00–6:20: PowerPoint lecture^b^ (Appendix F) • 6:20–6:30: wrap-up^d^	• PowerPoint, internet projector • Activity: jelly beans and a jar • Evaluation: online survey software	• Students completed the postcourse questionnaire (Appendix M) at the end of the session • Students completed the posttraining questionnaire (Appendix S) 8 months after course completion

a Consisting of a greeting, overview of session goals and objectives, and introduction of the faculty facilitator.

b Consisting of a PowerPoint taught by the faculty facilitator with teaching points, as well as time for questions and discussion.

c Consisting of an interactive team-based activity as a skill-building exercise to reinforce learning objectives.

d Consisting of closing remarks and instructions to complete a postsession questionnaire.

### Course Evaluation

We developed a robust evaluation according to the Kirkpatrick four-level assessment model for mentoring students.^12,13^ The students completed a precourse survey to assess baseline leadership skills prior to the start of the course. Following the last session, students completed a postcourse survey, which contained questions identical to those in the precourse questionnaire, to assess improvement and measure learning (Appendix M). Additionally, 8 months after completion of the course, participants completed a posttraining survey to gather information regarding the impact of the course on participant behavior over time as a measure of the effects of behavior change (Appendix S). On this survey, questions addressed skill utilization, skill confidence and application, and leadership aspiration and achievement. We developed session-specific questionnaires to assess participant reaction to and knowledge acquisition from each session, including questions that addressed satisfaction with session design, as well as participant proficiency related to the learning objectives of the session. These questionnaires were distributed to participants via email after each session (Appendices N–R). We utilized each session evaluation as indicated in Table 1. These evaluations were interpreted by course administrators according to the plan-do-study-act (PDSA) model for continued course evaluation and improvement. These participants received recognition of completion of the elective on their transcript. (The course was reviewed by the UMMS Institutional Review Board [IRB] for the distribution and analysis of participant surveys and was deemed exempt from IRB approval, “not human research.”)

### Course Logistics

We scheduled six 90-minute sessions over the spring semester. All sessions took place in a standard classroom at UMMS with internet and PowerPoint projector access. The two course developers provided additional low-budget materials, including paper, pencils, a deck of cards, and children's wooden building blocks. All student and faculty involvement was voluntary and volunteered. Course leaders and faculty communicated via email correspondence, as did course leaders and participants. Course leaders identified ideal faculty approximately 6 months prior to course scheduling and assessed interest via email invitation. The course developers created introductory teaching PowerPoint presentations for each session, which course faculty then adapted to suit their teaching needs (Appendices A–F). For evaluation, we created all questionnaires online using SurveyMonkey, a free online software program. We administered questionnaires to participants via email. Responses were anonymous. Each participant had a unique identifier (the last four digits of the participant's phone number) so that responses were anonymously matched for comparison pre- and postcompletion. We did not have access to this identifying information, and therefore, anonymity was preserved. All contact with facilitators/students was online. An email assessing course interest was sent to all first-year medical students, and first-year medical student participants were selected on a first-come, first-served basis.

## Results

Results were obtained from the first two offerings of the course. The review of the first offering (the pilot program) was positive overall, and the elective was approved for continuation, with very few changes made between the first and second offerings. We continued to evaluate this course with an analysis of longitudinal data to identify additional outcomes and for purposes of continuous quality improvement.

For the two offerings, a total of 26 students completed the course, 12 first-year students completing the pilot course and 14 first-year students completing the second course; these students committed to attending each session. All participants completed the demographic survey, which revealed that 25% had previously completed some form of formal leadership training, ranging from service trip instructors to a professional development course in the army. All participants had held some prior leadership position; the majority (75%) had been student leaders of undergraduate and graduate student body and organizational groups. Other leadership roles included college tutors, sport captains, and involvement in Greek life. All students took the elective to learn more about effective leadership and to further their interest in pursuing a leadership position either while in medical school or afterward.

**Figure 1.**
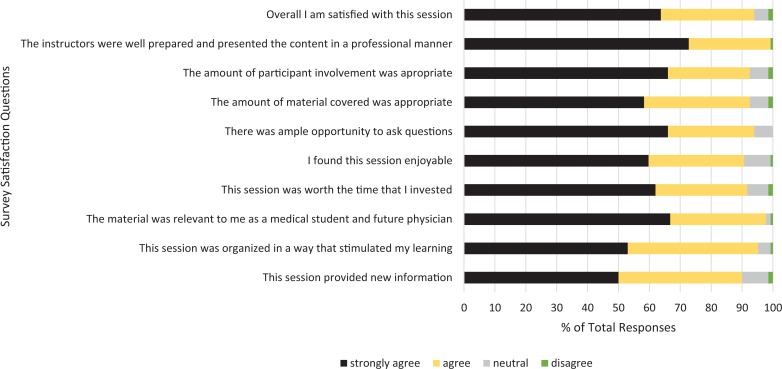
Cumulative results of participant satisfaction with each session (Kirkpatrick level 1). Participants rated their agreement with the listed statements as strongly disagree, disagree, no opinion, agree, or strongly agree after each session. The numbers of participant responses of each type in the pilot (*N* = 12) and second (*N* = 14) sessions were combined and expressed as a percentage of total responses.

Overall, participants demonstrated high satisfaction with the course and measurable learning, and they planned to utilize skills in future practice. Figure 1 displays the results of participant satisfaction. The Kirkpatrick model was used by course administration when creating a program evaluation to appropriately measure learning and impact of training. Table 2 organizes the results based on the Kirkpatrick evaluation.

**Table 2.** Kirkpatrick Four Levels of Training Evaluation

Level	Description	Assessment Tool	Results
1. Reaction	Measures how students felt, personal reaction to the training or learning experience	• Postsession questionnaires assessed reaction to individual sessions • Postcourse questionnaire assessed reaction to course overall • Open-ended feedback comments provided opportunity for subjective feedback not otherwise addressed	• Satisfaction with individual session components was high, and following the pilot course, 100% indicated that they would recommend this course to others (Figure 1). • Applicability: 100% felt topics were applicable in the near future to both medical student curriculum and future medical career paths. • Review of subjective feedback showed mostly positive comments, common themes emerging with satisfaction regarding participant interaction, establishment of peer-mentor relationships, and applicability.
2. Learning	Measures gain of knowledge or capability before versus after the course	• Pre-/postcourse questionnaire measured learning and confidence in skills	• Results showed significantly increased confidence in skills and knowledge related to all aspects of leadership compared to participants' level of confidence before the start of the program (Figure 2).
3. Behavior	Measures the extent to which the students applied the learning and changed their behavior	• Reflective writing assignments acted as a surrogate measure of predicted behavior change • Posttraining questionnaire measured utilization of skill 8 months later	• Considering that this course was relatively brief, it was difficult to measure direct training effects on behavior. All participants predicted use of skills in future scenarios and anticipated barriers to skill application. • Unfortunately, only one participant has completed the posttraining questionnaire to date from the pilot course; however, results were positive, with 100% utilization of skills applied on a regular basis.
4. Results	Measures the long-term impact of the training	• Final reflective writing assignment, projecting 1-, 3-, and 5-year achievement goals	• All students projected 1-, 3-, and 5-year leadership achievement goals with appropriate road map development.

**Figure 2.**
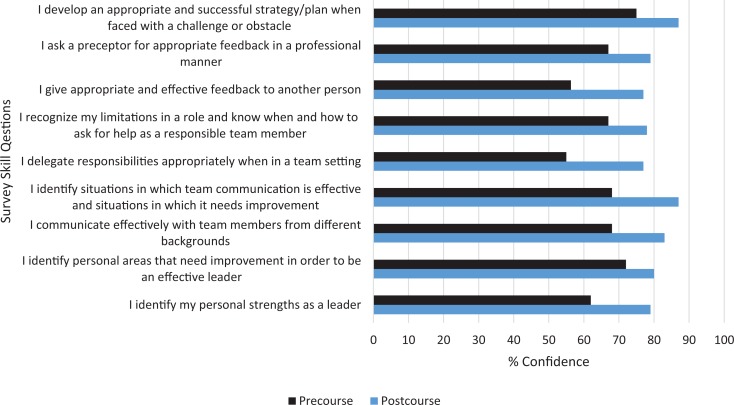
Participant confidence in ability to utilize pertinent knowledge and skills (Kirkpatrick level 2). Participants rated their confidence in skills and knowledge before and after the program on a scale of 1 (not at all confident) to 100 (very confident). Participant responses in the pilot (*N* = 12) and second (*N* = 14) sessions were combined, and means were calculated for each item. The pre- and postcourse responses are statistically significant ( *p* < .001), as determined by paired *t* test.

Our major aim was to create a course for students to learn leadership skills. Learning was assessed by comparing results of the pre- and postcourse surveys. By using a graphic scale model for survey questions, changes in participants’ perceived ability were measured, regardless of the starting point.^8^ A successful learning process was demonstrated by increased confidence in participants’ aptitude to apply and utilize the course information. Results showed an average increase in confidence of 11.2 points per area, although there was large variation per question (3.2–19.9 points). The largest average point increase occurred in relation to delegating responsibilities and giving and receiving feedback. In addition, responses to questions measuring confidence in knowledge and skill addressing session-specific learning objective competencies were high, with an average of 4.4 on the 5-point Likert scale (with self-assessment ratings falling between agree and strongly agree). The highest scores resulted from sessions teaching communication and feedback. Figure 2 displays the results of participant learning.

Furthermore, in relation to learning leadership skills, a secondary goal was to provide relevant skills in both immediate and long-term application. Postcourse skill utilization was assessed by the posttraining survey distributed 8 months after the course, reflecting interim skill use (Appendix S). Unfortunately, only one student from the pilot course completed this survey (one of 12 participants, completion rate: 8%). However, results indicate that course skills were very applicable to daily life; this participant incorporated leadership style insight, as well as practicing giving feedback and delegating responsibilities in relation to obtaining two new leadership positions in medical extracurricular activities. The posttraining survey was not distributed in the second course offering. Long-term skill utilization was assessed via the postcourse evaluation, on which all students appropriately projected anticipatory skill utilization as a surrogate marker for future application (Table 2).

## Discussion

We designed, implemented, and evaluated a successful leadership course for first-year medical students. The evaluation demonstrated that students enhanced their confidence in their leadership competencies and were highly satisfied with the course. The sessions were designed in a specific order; each session individually addressed educational objectives, and the learning competencies served as building blocks for further application. At the completion of the course, results demonstrated that students possessed qualities of effective student-physician leaders, showed objective changes in confidence related to leadership skills, experienced high confidence in their skills and abilities, and were motivated to attain leadership positions in the future. Additionally, we found that students engaged in this experience, reflected critically on their learning, and intended to apply that learning in the future.

We attribute the success of the course to multiple factors. First, the course competencies were selected based on prior research and publication in the field of medical leadership education. Second, the course was developed from the framework of student-centered learning, with most of the time dedicated to student-led discussion. This contributed to student engagement, individualized learning, and personal application. Third, the course promoted student-faculty relationships by facilitating personalized encounters with faculty holding high-level leadership positions. This interaction fostered a sense of student support and interest, as well as collaboration opportunities. Fourth, the course utilized multiple teaching methods to reach different types of student learners. This multifaceted approach encouraged student participation and partnerships and additionally promoted community and camaraderie. Finally, the design of a robust evaluation strategy utilizing an evidence-based and scholarly approach allowed assessment of the achievement of our overall goal, learning objectives, and outcomes, as well as highlighting areas for future improvement. We created an evaluation tool that captured and quantified the immediate course impact on learning and anticipated future application in a way that was sustainable, low budget, and adaptable to user objectives. Confidence measure of skills was subjective, with multifactorial influence, including self-awareness of skill building, opportunity for skill incorporation and practice, and time frame of achievement.

Here, we have described the development and piloting of the course. At the time of submission, the course was in its third offering; popularity grew, and enrollment increased. We took the opportunity to evaluate our design based on participant and instructor feedback and then refined our approach. We learned that when creating curricula, it was critical to develop a PDSA evaluation concurrently to ensure that objectives were adequately assessed. The PDSA format inherently promoted continued evaluation and revision via analysis of participant satisfaction on postsession assessments. This OEE was created at an institution with a standardized process of review of student-created courses, which helped ensure that course goals and objectives were attainable via course design. Two student codirectors were involved in pilot course development and implementation, and the need for additional student administrative effort has expanded due to the logistical requirements of scheduling, faculty recruitment, student recruitment, survey administration and follow-up, and so on. With the heavy emphasis on keeping the offering a student-led course, we recommend having two or more codirectors share logistical work. Recognizing participant time and effort was critical to involvement and engagement, and participants were awarded credit on their medical school transcript for course completion.

A major success in the development of the course was that it was student initiated and designed. Students identified a gap in the curriculum and were passionate advocates for addressing their learning needs. Course creators were invested learners and willing to participate above the requirements of the standard curriculum. Therefore, this course was developed without a supporting budget and was an extremely cost-effective mechanism for curriculum development. Very few financial resources were required, as the course was developed utilizing volunteer student labor, faculty willing to volunteer their time, and use of school facilities without charge.

Although the course was low cost, our call for all physicians to master leadership skills lends itself to the assumption that we anticipated the course expanding above the level of volunteer financial resources. The need to increase resources if adapting the course into a standard medical school curriculum may be a limitation and a potential future direction. Our institution afforded a multitude of qualified faculty support; however, as a second limitation, we recognize that volunteer faculty participation may vary at other institutions based on participant and institution time constraints and/or availability and, therefore may merit some form of recognition or compensation. In concert with employing professionally established faculty, as a third limitation, we recognize that although our volunteer teaching staff held professorship or senior management positions, this may be less available and generalizable to other institutions. Given that we feel strong leadership and teaching skills are needed by all caretakers functioning in a team setting, any staff deemed a role model in this skill set (e.g., a department chair or division chief) could be asked to partake at the discretion of the course administrator(s).

Our results demonstrated that students increased competence in their leadership skills; however, we realize that our results might have been more robust if proficiency were rated before and after each session, as each individual participant had prior leadership experience or training that could have biased the participant's baseline skill confidence. As a future direction, we plan to obtain these data in future course offerings. Anticipated variation of pursuit of postcourse participant experiences made a projected time frame for tangible Kirkpatrick level 3 data collection complicated and likely reflected the high rate of loss to follow-up on the posttraining questionnaire. Although our results for the one participant who completed the posttraining questionnaire were encouraging, we understood the possibility for a biased interpretation. So, we inherently anticipated and proactively addressed and obtained level 3 Kirkpatrick results, as students appropriately predicted and projected the impact this course would have on their immediate and long-term future career as a surrogate indicator. Therefore, the efficacy of our course will be substantiated by the applicability of the training in participant clinical practice with extrapolation of impact on future achievement. In conjunction with Kirkpatrick level 4 results, as a future direction, we will track student participants' involvement in scholarly, volunteer, and academic leadership achievement roles across their 4 years of medical school and beyond in comparison with their peers as a longitudinal evaluation of course effect. In addition, as a future direction, it will be of interest to evaluate the leadership and teaching skills gained by the codirectors after leading the course. Given the ease of adaptability and implementation, it is our hope to expand the course, as this offering would be of benefit as an interprofessional course available to students studying medicine, nursing, and biomedical science. We plan to continue to utilize the evaluation tool as part of a PDSA program to enhance future offerings. Conducting an analysis for level 4 impact is scholarly research requiring longitudinal assessment, with reliability measures requiring a larger sample size attained from subsequent course implementation. We feel confident in our ability to address this utility gap with course modification in response to results from our evaluation.

The importance of leadership and teamwork skill training in UME is well recognized.^14^ However, standard curricula and methods of instruction for UME institutions do not exist, leading to varied training experiences.^7^ Barriers to implementation of such training include cost, time, and lack of research outcomes on the most efficacious structure.^4^ We have developed a successful leadership course in alignment with nationally recognized core leadership competencies that is cost effective, succinct, and sustainable. Additionally, we offer a scholarly evaluation that quantifies learning and can easily be adapted by other institutions. We offer this model to other medicals schools working to implement or augment curricula in leadership development.

## Appendices

A. Session 1 PPT Leadership Styles.pptx
B. Session 2 PPT Teamwork.pptx
C. Session 3 PPT Delegation.pptx
D. Session 4 PPT Feedback.pptx
E. Session 5 PPT Direction.pptx
F. Session 6 Optional Review PPT Consolidation.pptx
G. Session 1 Activity Instructions.docx
H. Session 2 Activity Instructions.docx
I. Session 3 Activity Instructions.docx
J. Session 4 Activity Instructions and Figure.docx
K. Session 5 Activity Instructions.docx
L. Session 6 Activity Instructions.docx
M. Precourse and Postcourse Evaluation.docx
N. Session 1 Evaluation.docx
O. Session 2 Evaluation.docx
P. Session 3 Evaluation.docx
Q. Session 4 Evaluation.docx
R. Session 5 Evaluation.docx
S. Posttraining Evaluation.docx
T. Supplemental Alternative Activity - PACE Palette.docx
U. Supplemental Alternative Activity - ACLS Video.docx
V. Supplemental Alternative Activity - Feedback Video.docx All appendices are peer reviewed as integral parts of the Original Publication.
