# Supplementary material for: Preparing Medical Students to Be Physician Leaders: A Leadership Training Program for Students Designed and Led by Students
Source: MedEdPORTAL. 2019 Dec 13;15:10863. doi: 10.15766/mep_2374-8265.10863 (PMC7012310; doi:10.15766/mep_2374-8265.10863)
Supplement: Supplementary file 1 — A. Session 1 PPT Leadership Styles.pptx B. Session 2 PPT Teamwork.pptx C. Session 3 PPT Delegation.pptx D. Session 4 PPT Feedback.pptx E. Session 5 PPT Direction.pptx F. Session 6 Optional Review PPT Consolidation.pptx G. Session 1 Activity Instructions.docx H. Session 2 Activity Instructions.docx I. Session 3 Activity Instructions.docx J. Session 4 Activity Instructions and Figure.docx K. Session 5 Activity Instructions.docx L. Session 6 Activity Instructions.docx M. Precourse and Postcourse Evaluation.docx N. Session 1 Evaluation.docx O. Session 2 Evaluation.docx P. Session 3 Evaluation.docx Q. Session 4 Evaluation.docx R. Session 5 Evaluation.docx S. Posttraining Evaluation.docx T. Supplemental Alternative Activity - PACE Palette.docx U. Supplemental Alternative Activity - ACLS Video.docx V. Supplemental Alternative Activity - Feedback Video.docx [file mep-15-10863-s001.zip › A. Session 1 PPT Leadership Styles.pptx]

## Slide 1
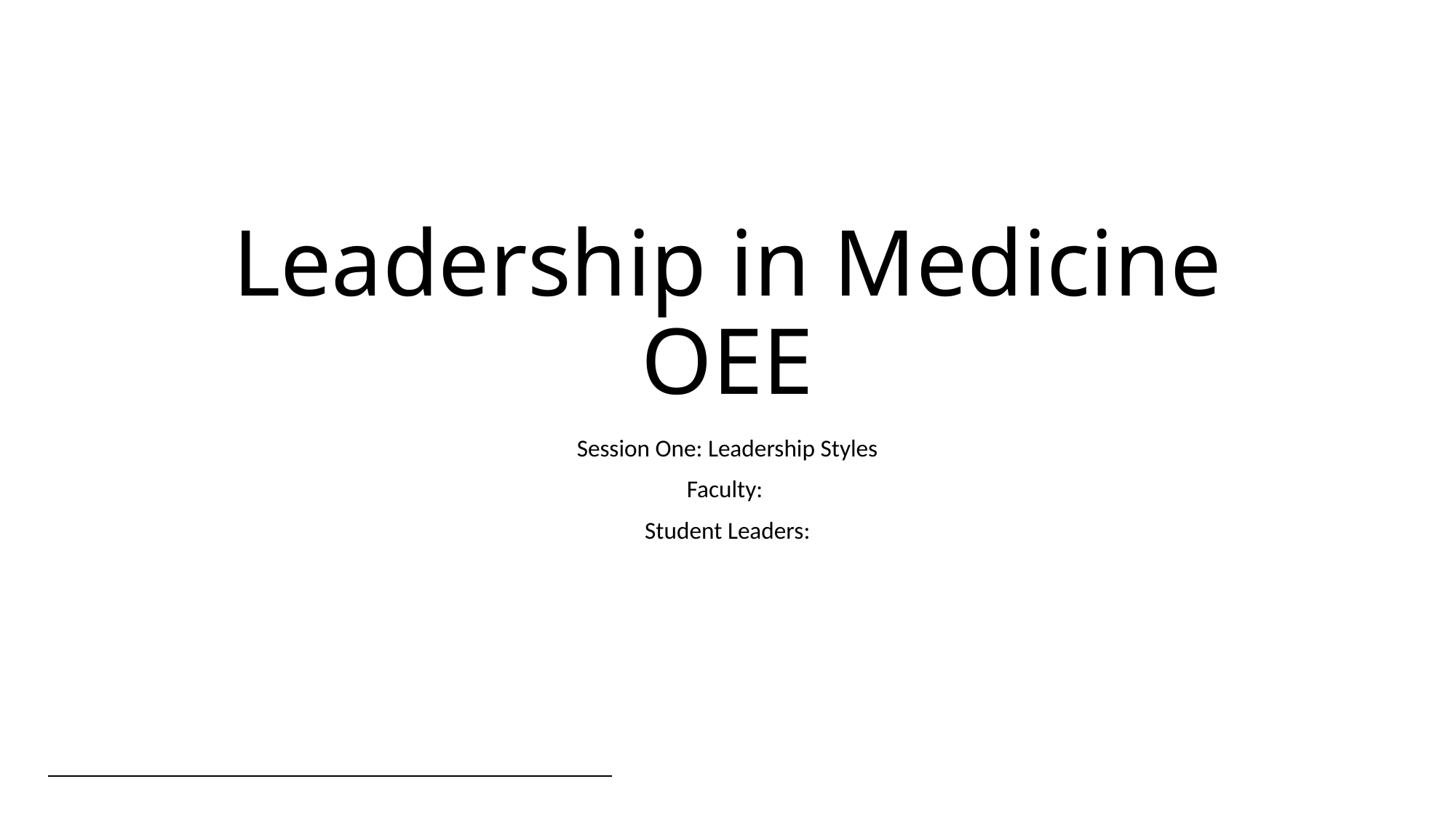

# Leadership in Medicine OEE
Session One: Leadership Styles
Faculty:
Student Leaders:
| |
| --- |

## Slide 2
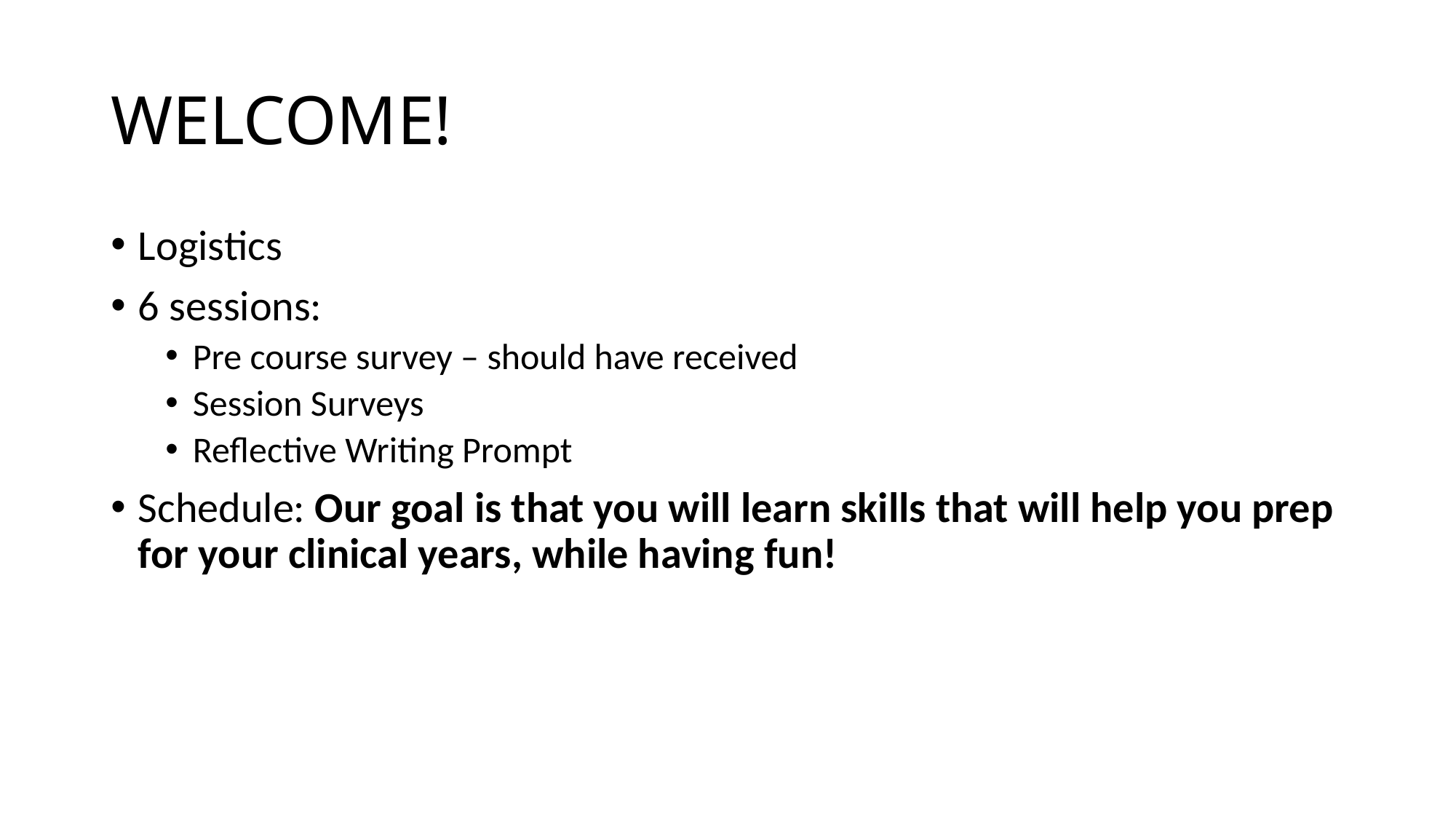

# WELCOME!
Logistics
6 sessions:
Pre course survey – should have received
Session Surveys
Reflective Writing Prompt
Schedule: Our goal is that you will learn skills that will help you prep for your clinical years, while having fun!

## Slide 3
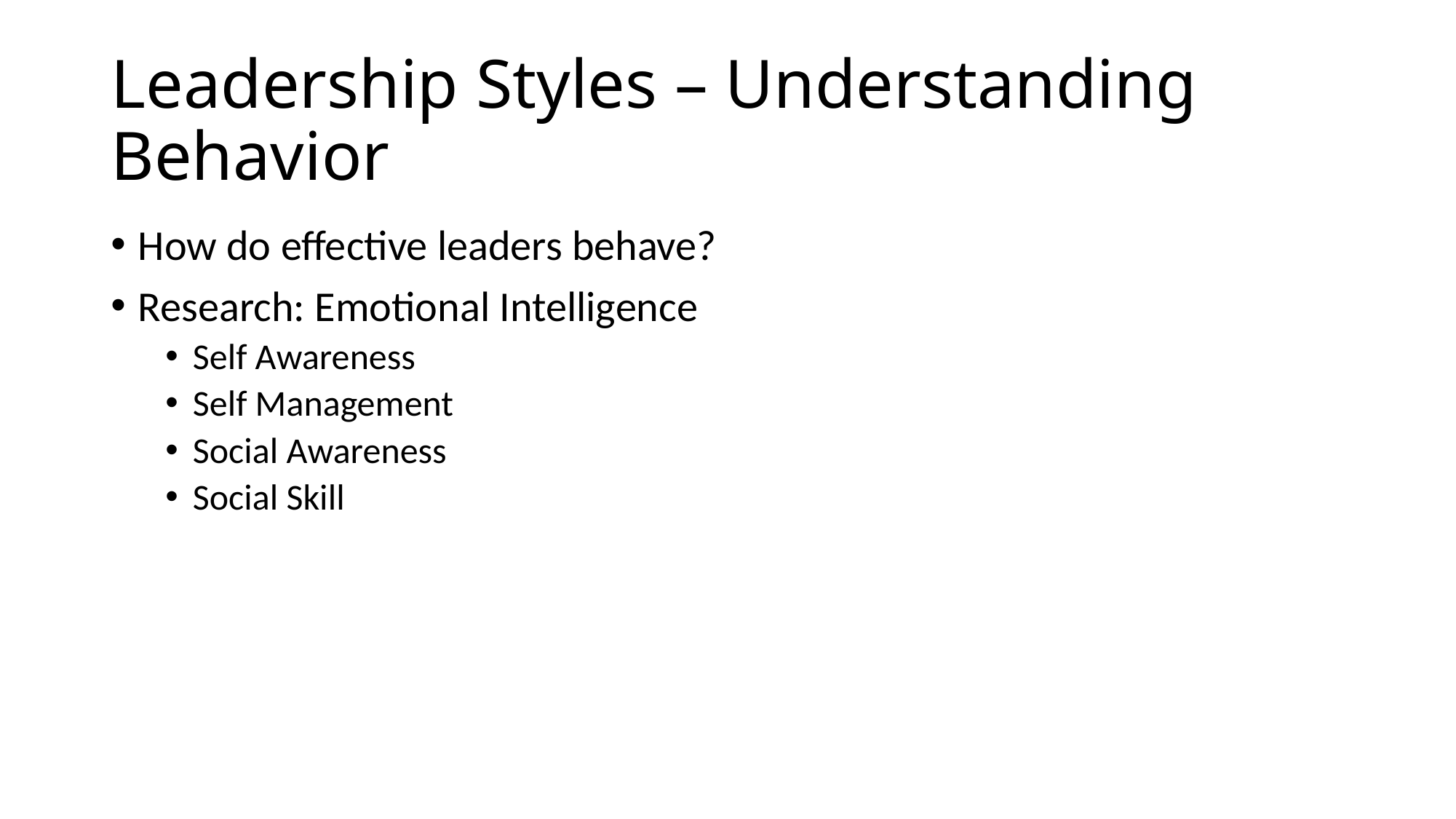

# Leadership Styles – Understanding Behavior
How do effective leaders behave?
Research: Emotional Intelligence
Self Awareness
Self Management
Social Awareness
Social Skill

## Slide 4
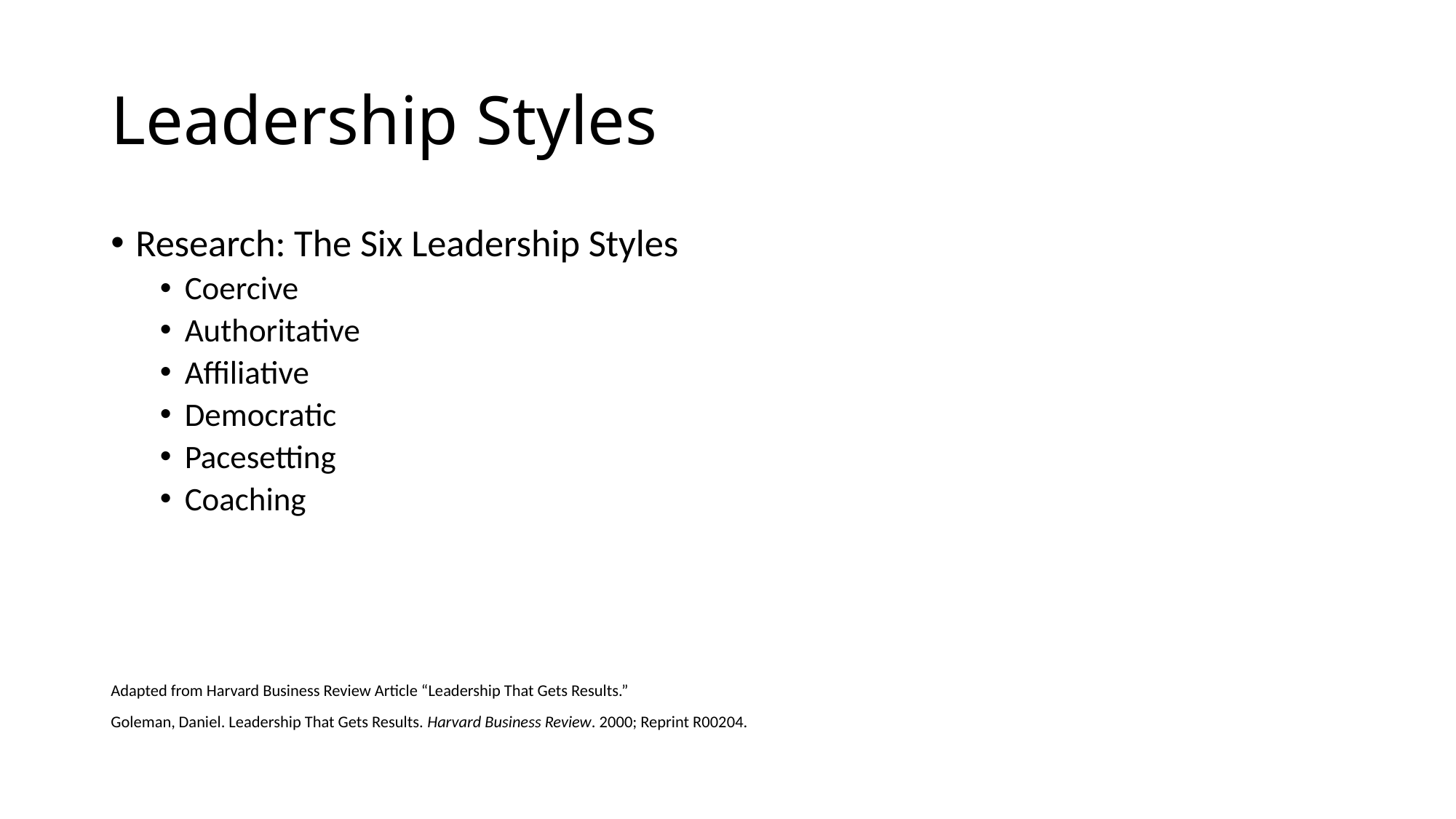

# Leadership Styles
Research: The Six Leadership Styles
Coercive
Authoritative
Affiliative
Democratic
Pacesetting
Coaching
Adapted from Harvard Business Review Article “Leadership That Gets Results.”
Goleman, Daniel. Leadership That Gets Results. Harvard Business Review. 2000; Reprint R00204.

## Slide 5
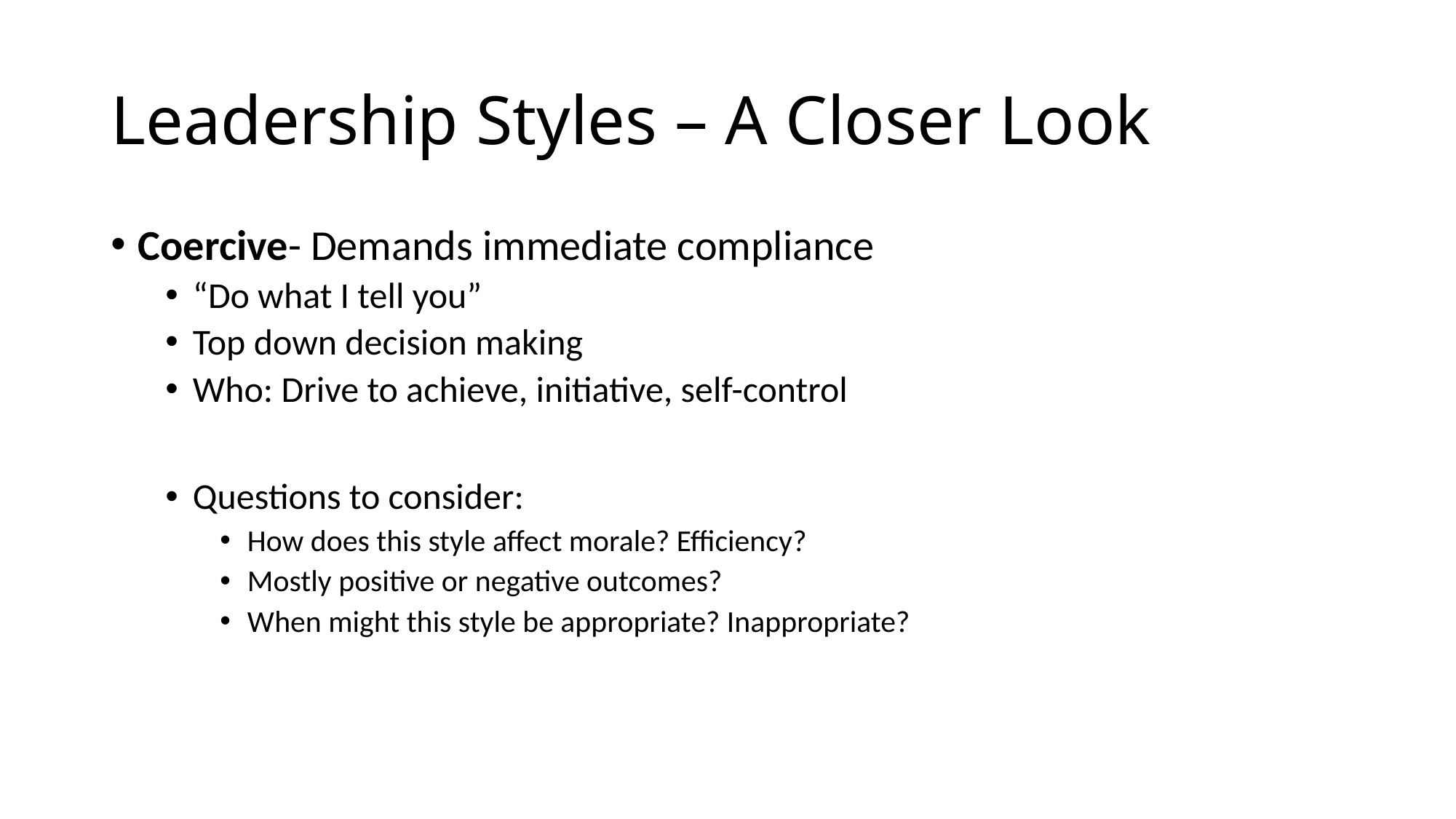

# Leadership Styles – A Closer Look
Coercive- Demands immediate compliance
“Do what I tell you”
Top down decision making
Who: Drive to achieve, initiative, self-control
Questions to consider:
How does this style affect morale? Efficiency?
Mostly positive or negative outcomes?
When might this style be appropriate? Inappropriate?

## Slide 6
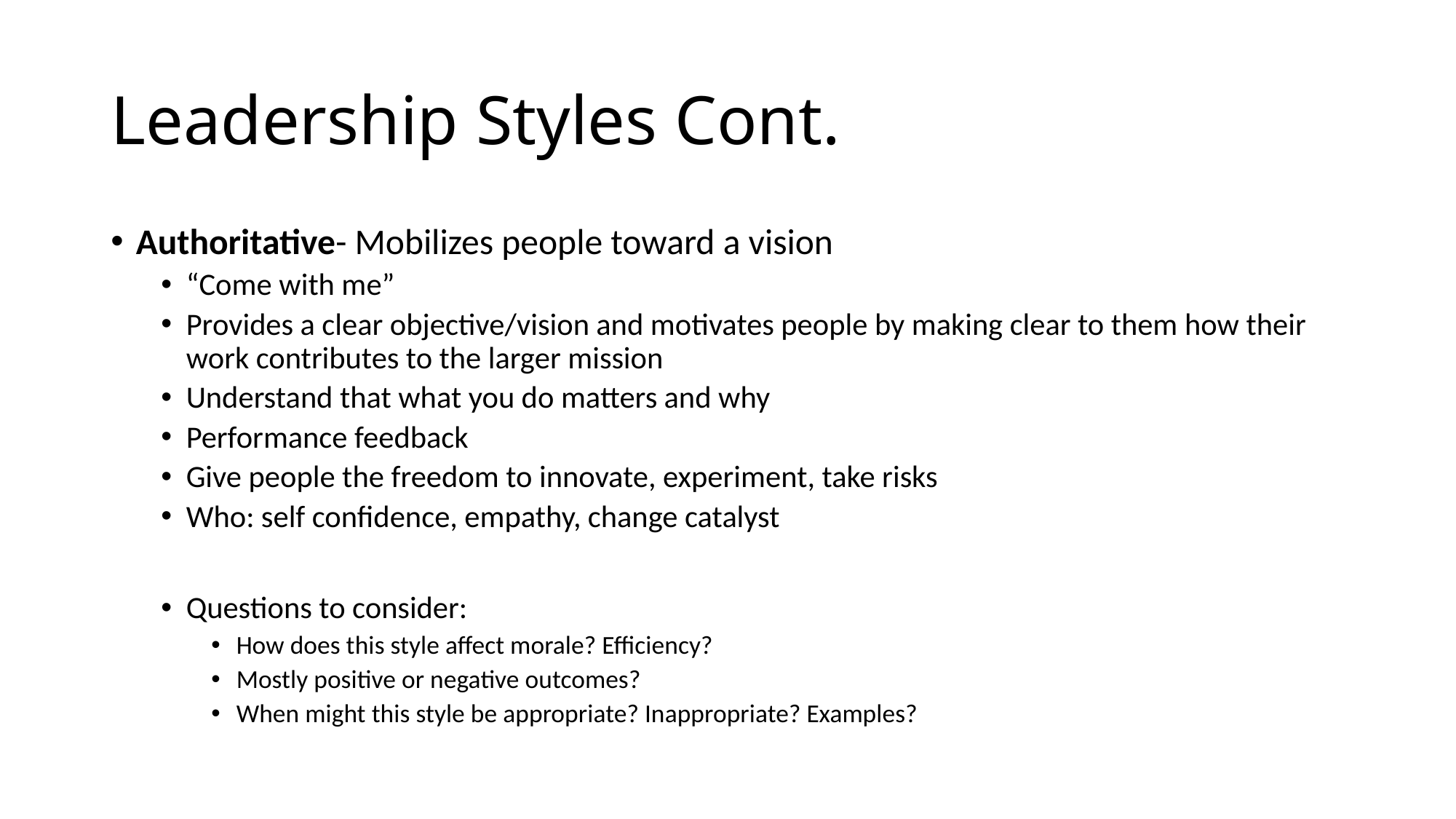

# Leadership Styles Cont.
Authoritative- Mobilizes people toward a vision
“Come with me”
Provides a clear objective/vision and motivates people by making clear to them how their work contributes to the larger mission
Understand that what you do matters and why
Performance feedback
Give people the freedom to innovate, experiment, take risks
Who: self confidence, empathy, change catalyst
Questions to consider:
How does this style affect morale? Efficiency?
Mostly positive or negative outcomes?
When might this style be appropriate? Inappropriate? Examples?

## Slide 7
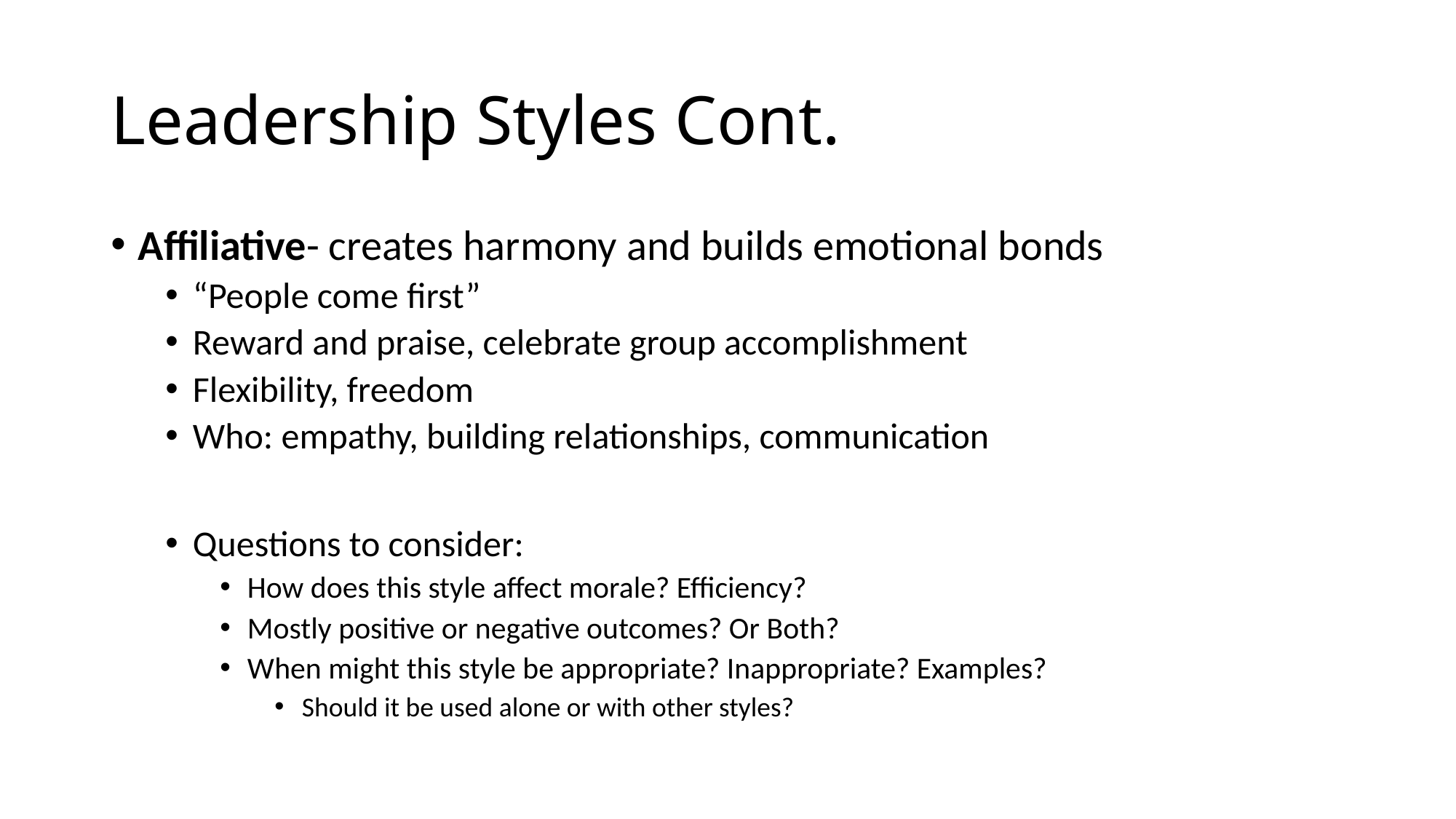

# Leadership Styles Cont.
Affiliative- creates harmony and builds emotional bonds
“People come first”
Reward and praise, celebrate group accomplishment
Flexibility, freedom
Who: empathy, building relationships, communication
Questions to consider:
How does this style affect morale? Efficiency?
Mostly positive or negative outcomes? Or Both?
When might this style be appropriate? Inappropriate? Examples?
Should it be used alone or with other styles?

## Slide 8
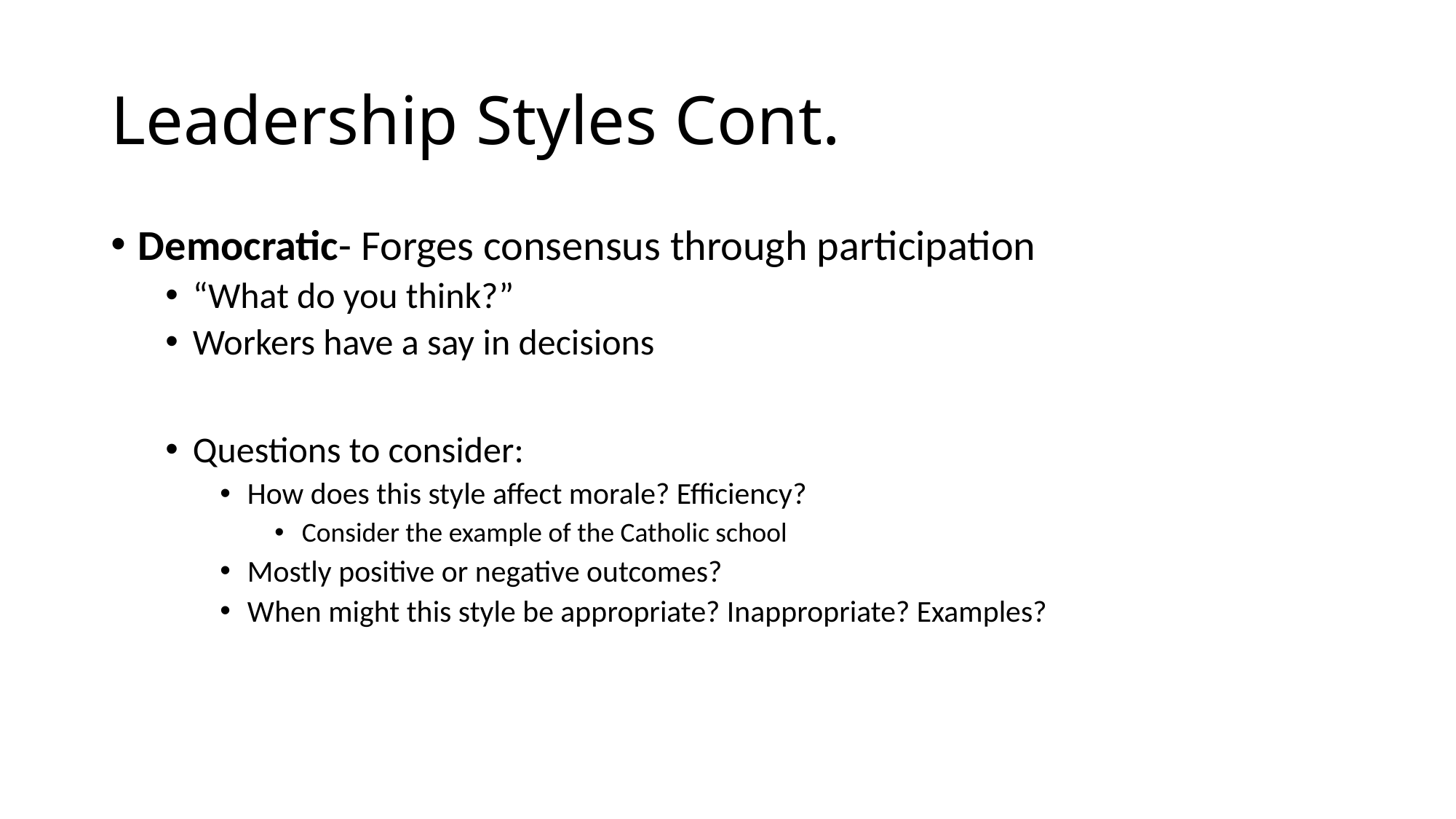

# Leadership Styles Cont.
Democratic- Forges consensus through participation
“What do you think?”
Workers have a say in decisions
Questions to consider:
How does this style affect morale? Efficiency?
Consider the example of the Catholic school
Mostly positive or negative outcomes?
When might this style be appropriate? Inappropriate? Examples?

## Slide 9
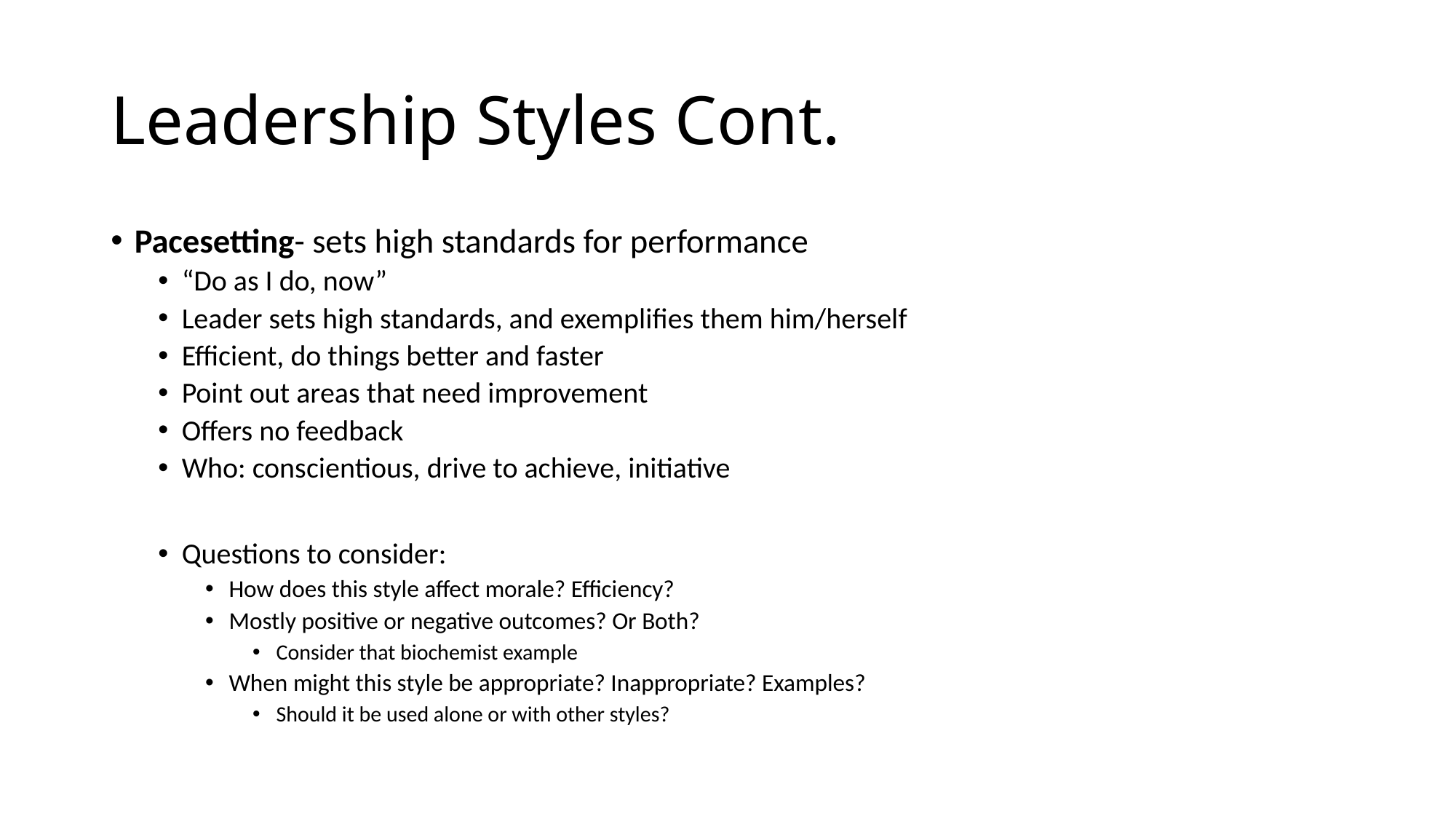

# Leadership Styles Cont.
Pacesetting- sets high standards for performance
“Do as I do, now”
Leader sets high standards, and exemplifies them him/herself
Efficient, do things better and faster
Point out areas that need improvement
Offers no feedback
Who: conscientious, drive to achieve, initiative
Questions to consider:
How does this style affect morale? Efficiency?
Mostly positive or negative outcomes? Or Both?
Consider that biochemist example
When might this style be appropriate? Inappropriate? Examples?
Should it be used alone or with other styles?

## Slide 10
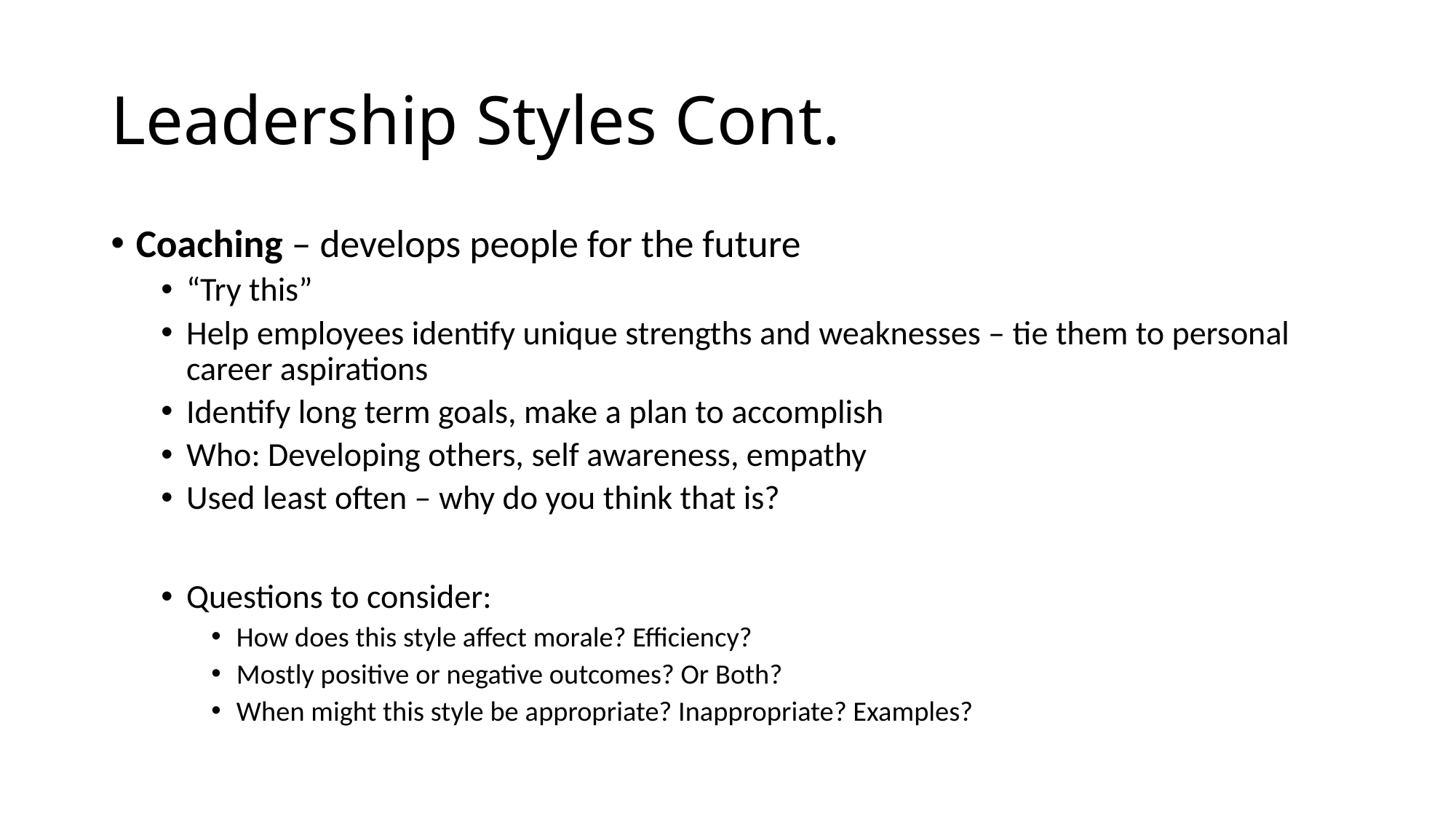

# Leadership Styles Cont.
Coaching – develops people for the future
“Try this”
Help employees identify unique strengths and weaknesses – tie them to personal career aspirations
Identify long term goals, make a plan to accomplish
Who: Developing others, self awareness, empathy
Used least often – why do you think that is?
Questions to consider:
How does this style affect morale? Efficiency?
Mostly positive or negative outcomes? Or Both?
When might this style be appropriate? Inappropriate? Examples?

## Slide 11
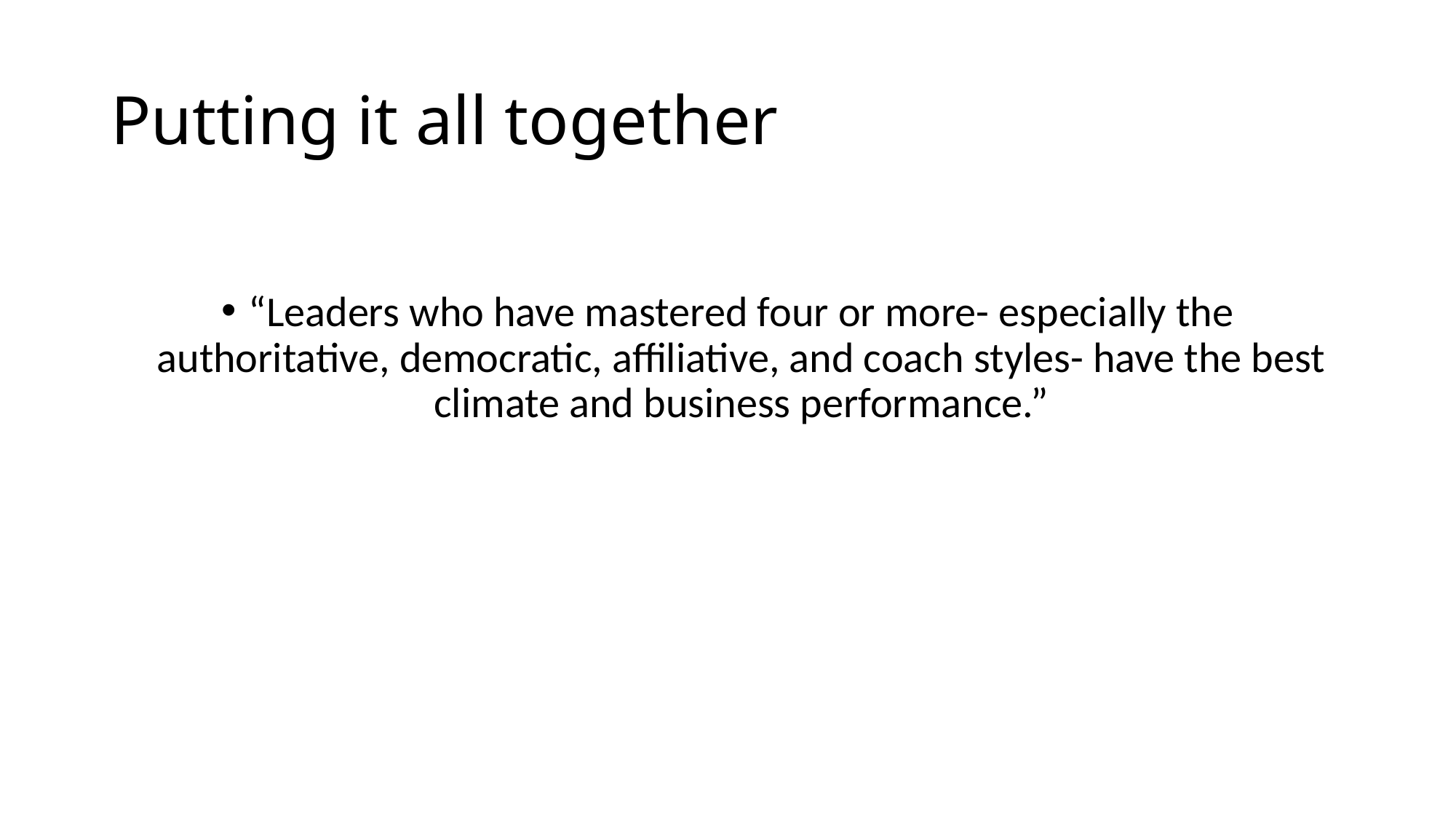

# Putting it all together
“Leaders who have mastered four or more- especially the authoritative, democratic, affiliative, and coach styles- have the best climate and business performance.”

## Slide 12
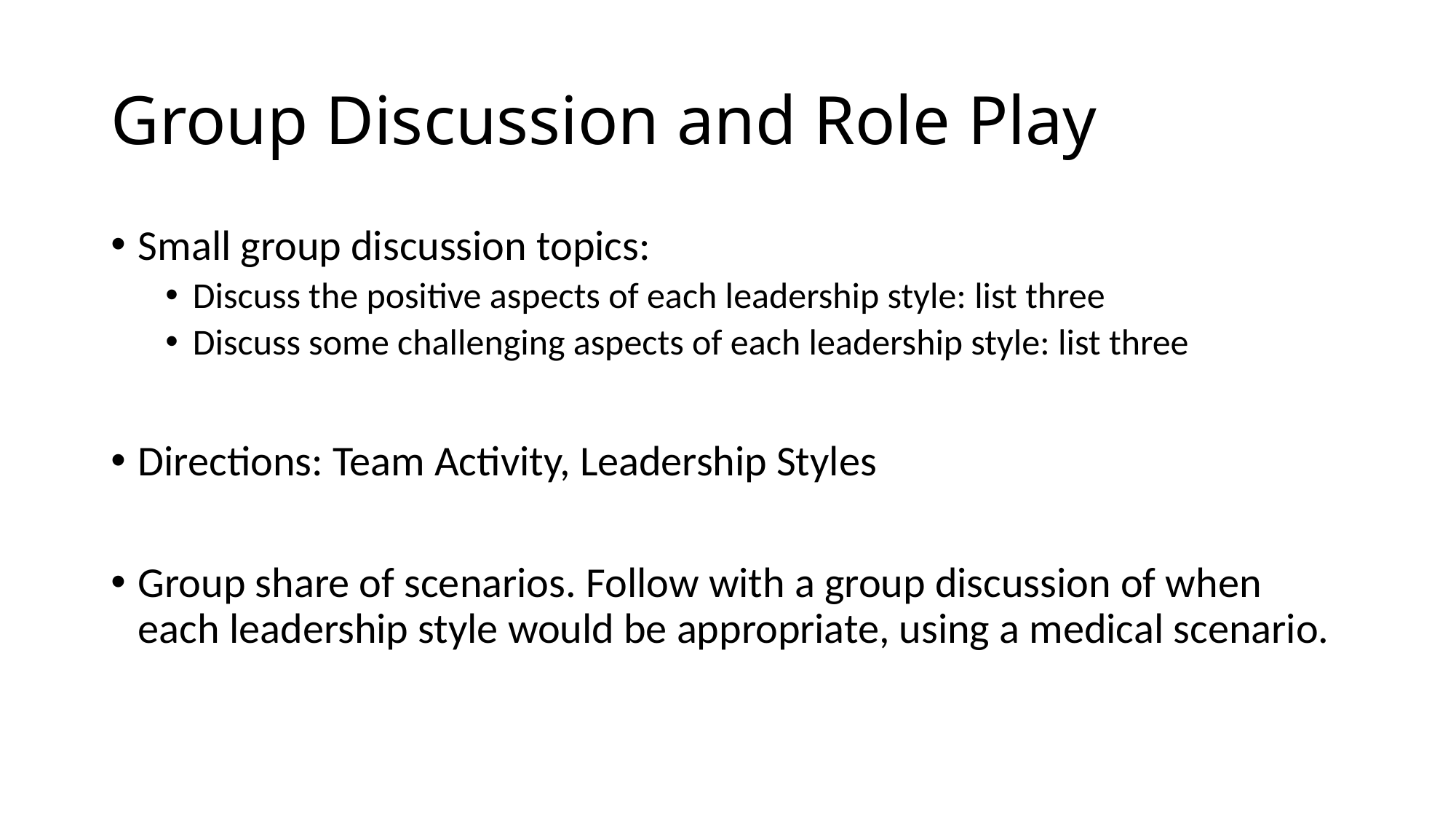

# Group Discussion and Role Play
Small group discussion topics:
Discuss the positive aspects of each leadership style: list three
Discuss some challenging aspects of each leadership style: list three
Directions: Team Activity, Leadership Styles
Group share of scenarios. Follow with a group discussion of when each leadership style would be appropriate, using a medical scenario.
