# Supplementary material for: Preparing Medical Students to Be Physician Leaders: A Leadership Training Program for Students Designed and Led by Students
Source: MedEdPORTAL. 2019 Dec 13;15:10863. doi: 10.15766/mep_2374-8265.10863 (PMC7012310; doi:10.15766/mep_2374-8265.10863)
Supplement: Supplementary file 1 — A. Session 1 PPT Leadership Styles.pptx B. Session 2 PPT Teamwork.pptx C. Session 3 PPT Delegation.pptx D. Session 4 PPT Feedback.pptx E. Session 5 PPT Direction.pptx F. Session 6 Optional Review PPT Consolidation.pptx G. Session 1 Activity Instructions.docx H. Session 2 Activity Instructions.docx I. Session 3 Activity Instructions.docx J. Session 4 Activity Instructions and Figure.docx K. Session 5 Activity Instructions.docx L. Session 6 Activity Instructions.docx M. Precourse and Postcourse Evaluation.docx N. Session 1 Evaluation.docx O. Session 2 Evaluation.docx P. Session 3 Evaluation.docx Q. Session 4 Evaluation.docx R. Session 5 Evaluation.docx S. Posttraining Evaluation.docx T. Supplemental Alternative Activity - PACE Palette.docx U. Supplemental Alternative Activity - ACLS Video.docx V. Supplemental Alternative Activity - Feedback Video.docx [file mep-15-10863-s001.zip › B. Session 2 PPT Teamwork.pptx]

## Slide 1
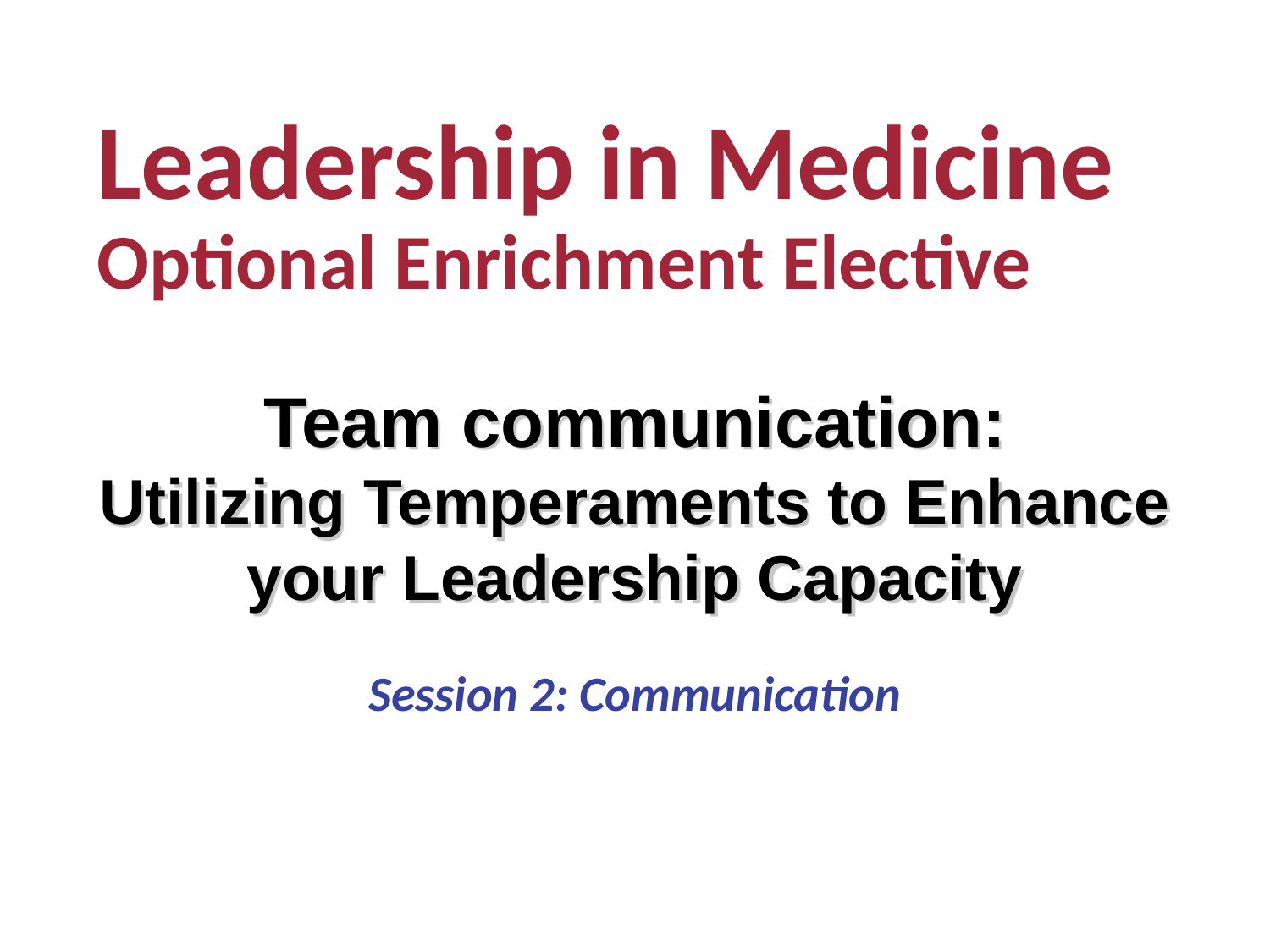

# Leadership in Medicine Optional Enrichment Elective
Team communication:
Utilizing Temperaments to Enhance your Leadership Capacity
Session 2: Communication

## Slide 2
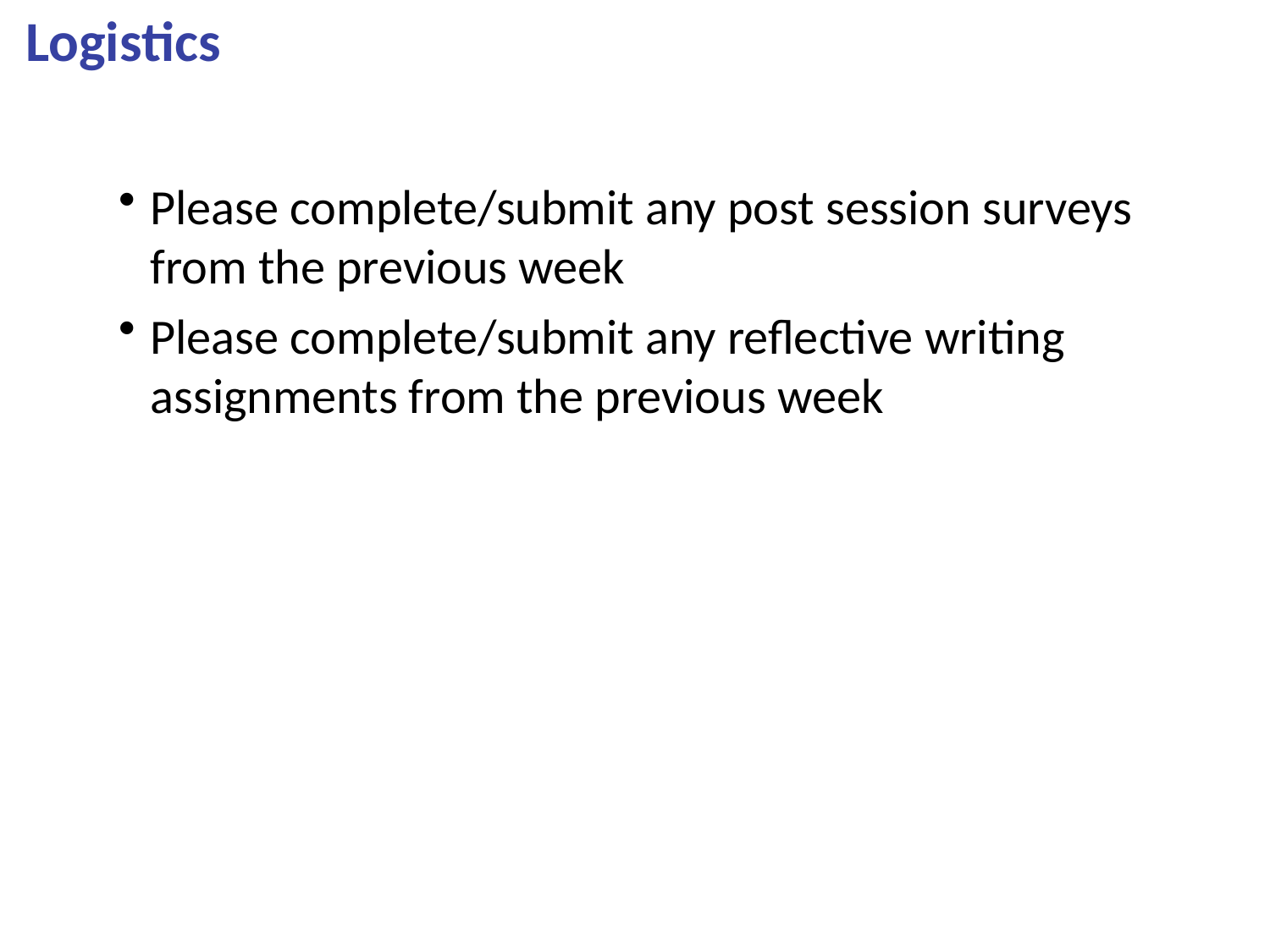

# Logistics
Please complete/submit any post session surveys from the previous week
Please complete/submit any reflective writing assignments from the previous week

## Slide 3
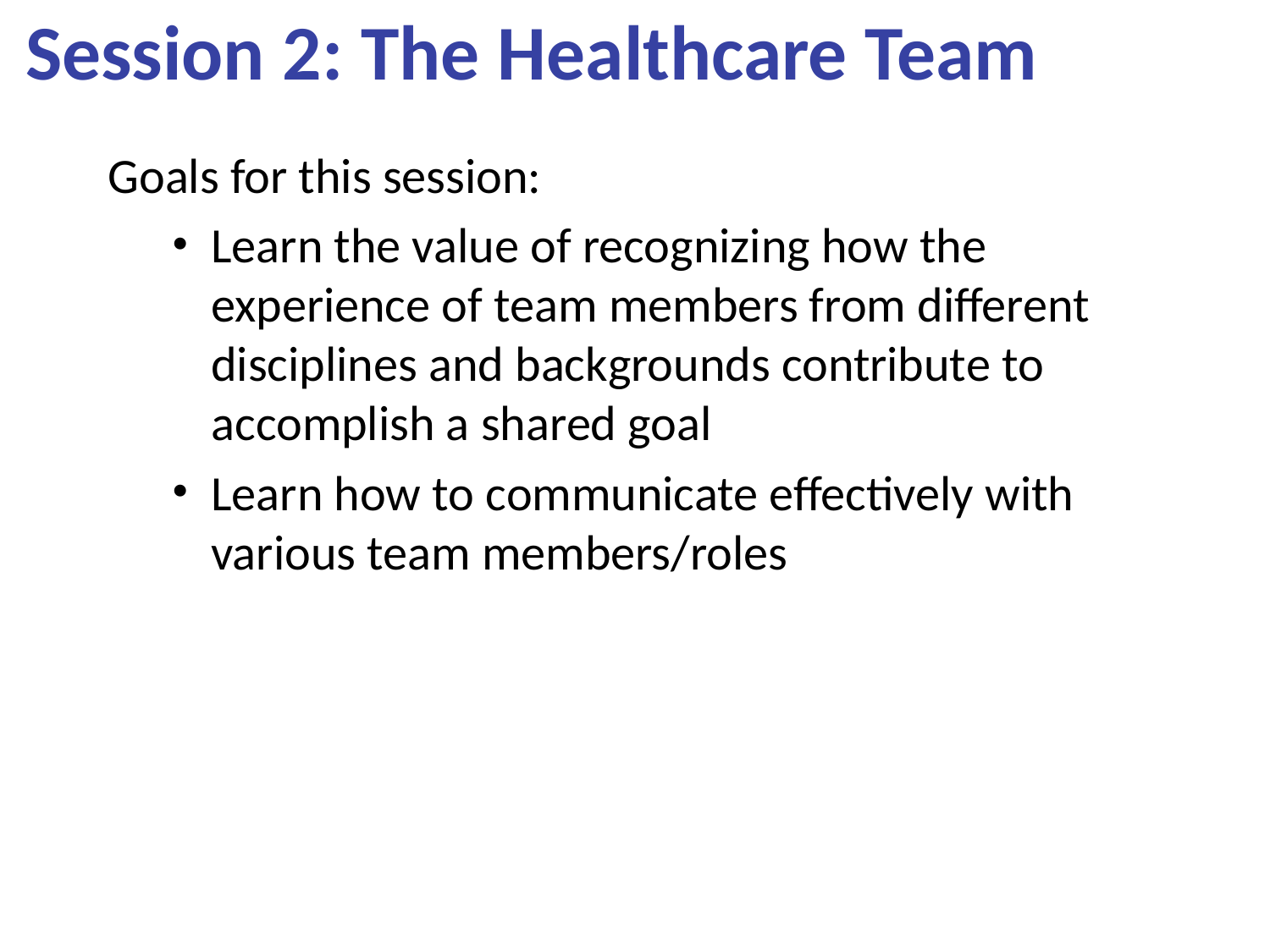

# Session 2: The Healthcare Team
Goals for this session:
Learn the value of recognizing how the experience of team members from different disciplines and backgrounds contribute to accomplish a shared goal
Learn how to communicate effectively with various team members/roles

## Slide 4
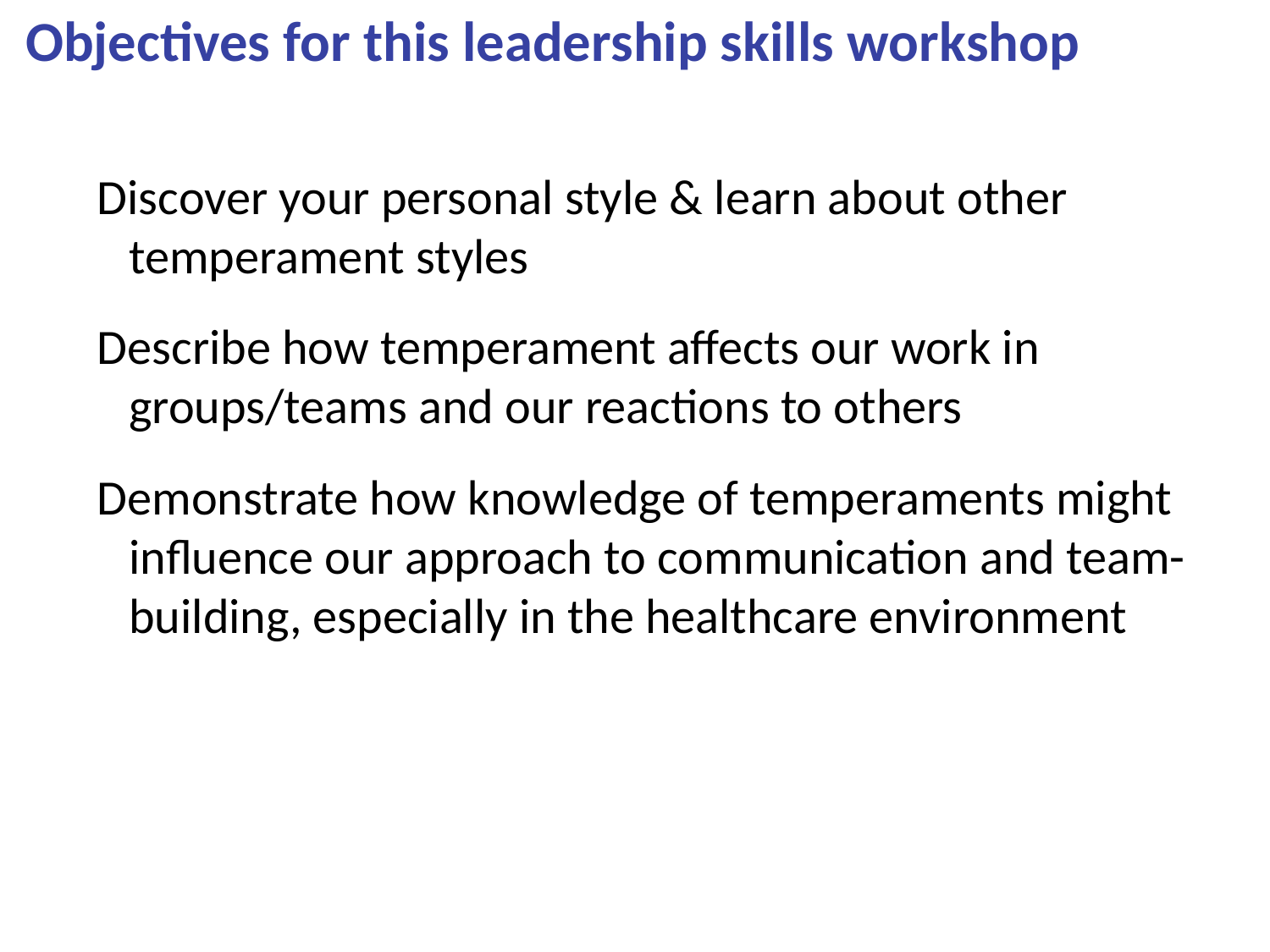

# Objectives for this leadership skills workshop
Discover your personal style & learn about other temperament styles
Describe how temperament affects our work in groups/teams and our reactions to others
Demonstrate how knowledge of temperaments might influence our approach to communication and team-building, especially in the healthcare environment

## Slide 5
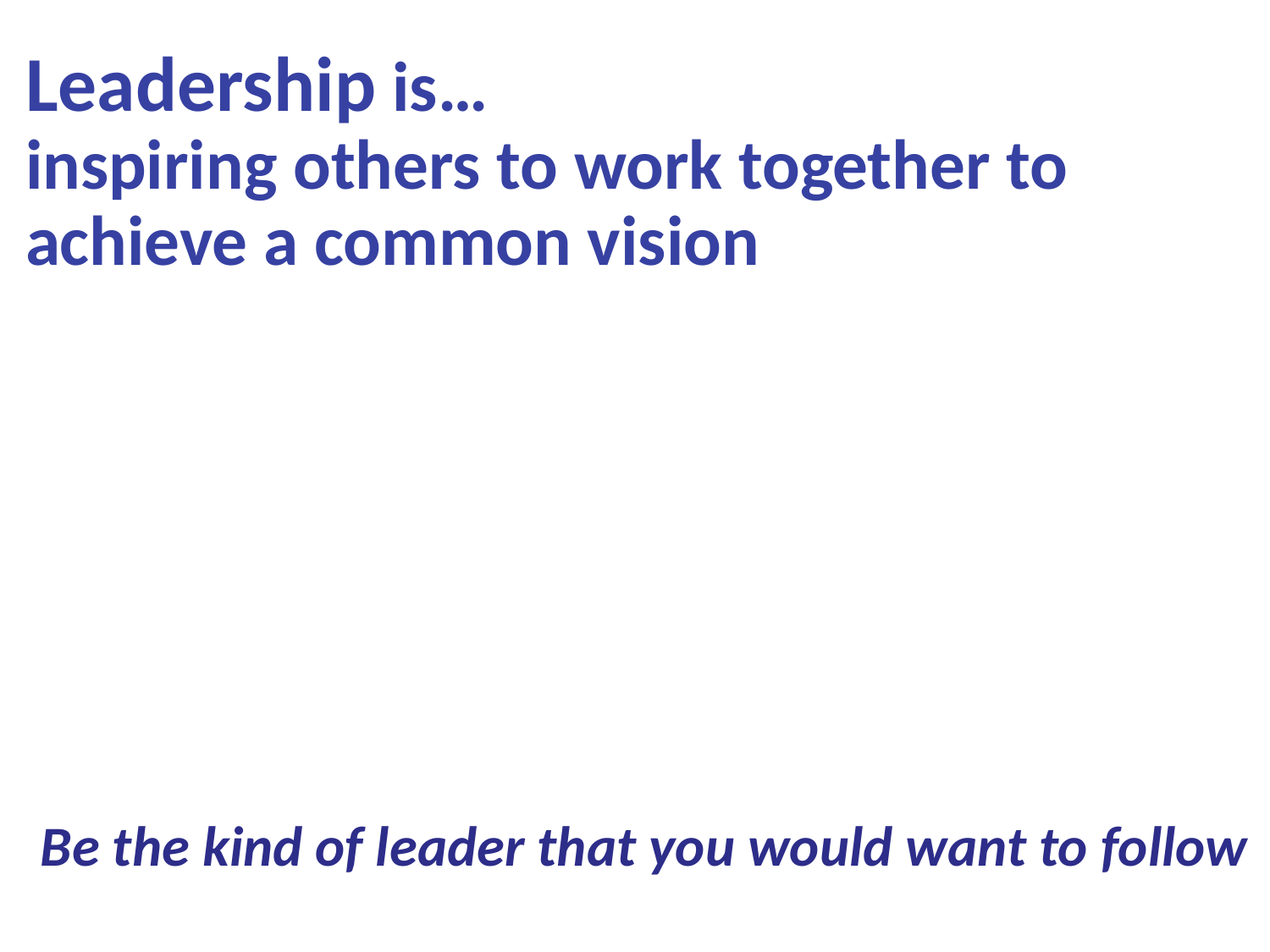

Leadership is…
inspiring others to work together to achieve a common vision
Be the kind of leader that you would want to follow

## Slide 6
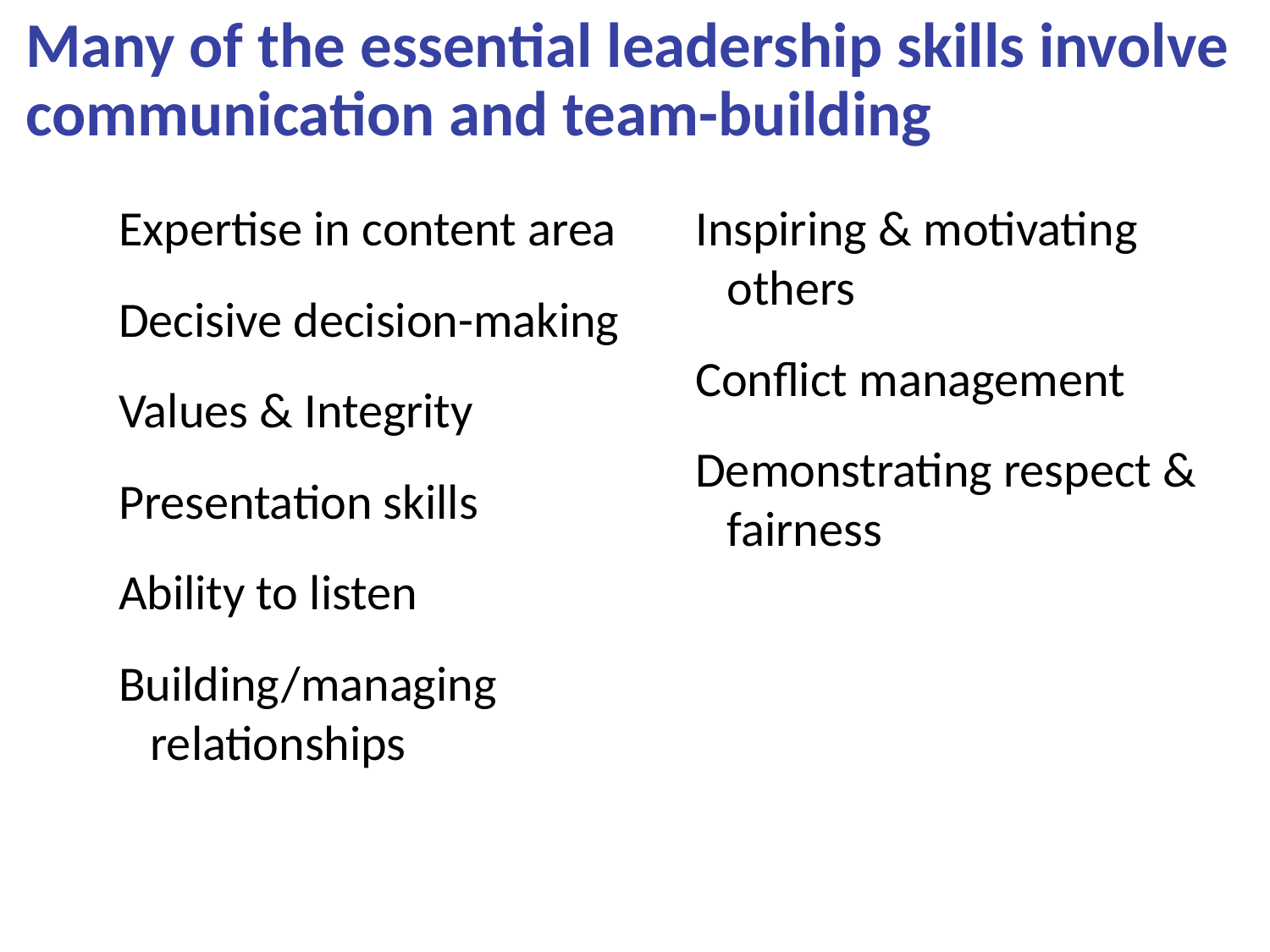

# Many of the essential leadership skills involve communication and team-building
Expertise in content area
Decisive decision-making
Values & Integrity
Presentation skills
Ability to listen
Building/managing relationships
Inspiring & motivating others
Conflict management
Demonstrating respect & fairness

## Slide 7
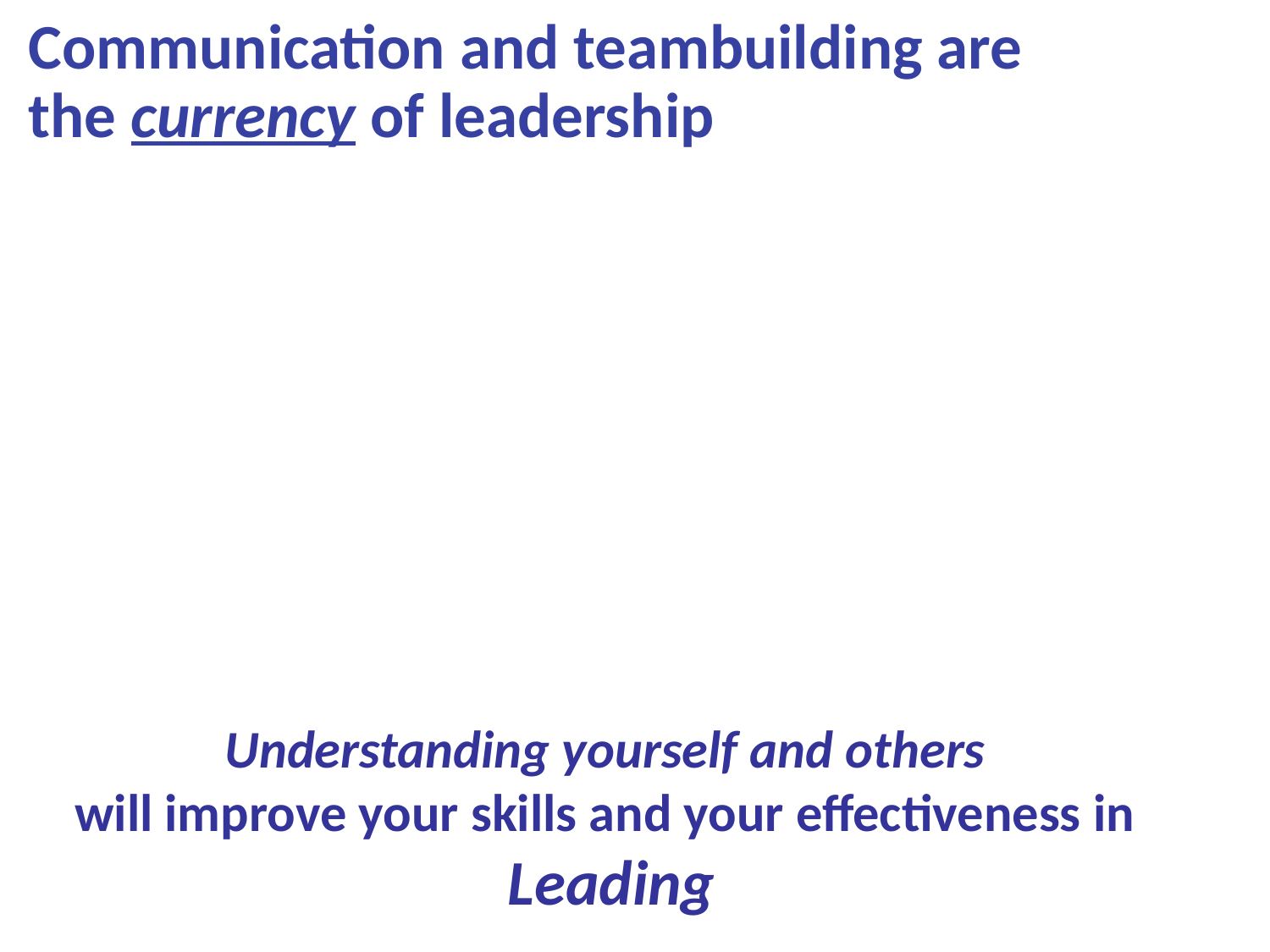

Communication and teambuilding are
the currency of leadership
Understanding yourself and others
will improve your skills and your effectiveness in
Leading

## Slide 8
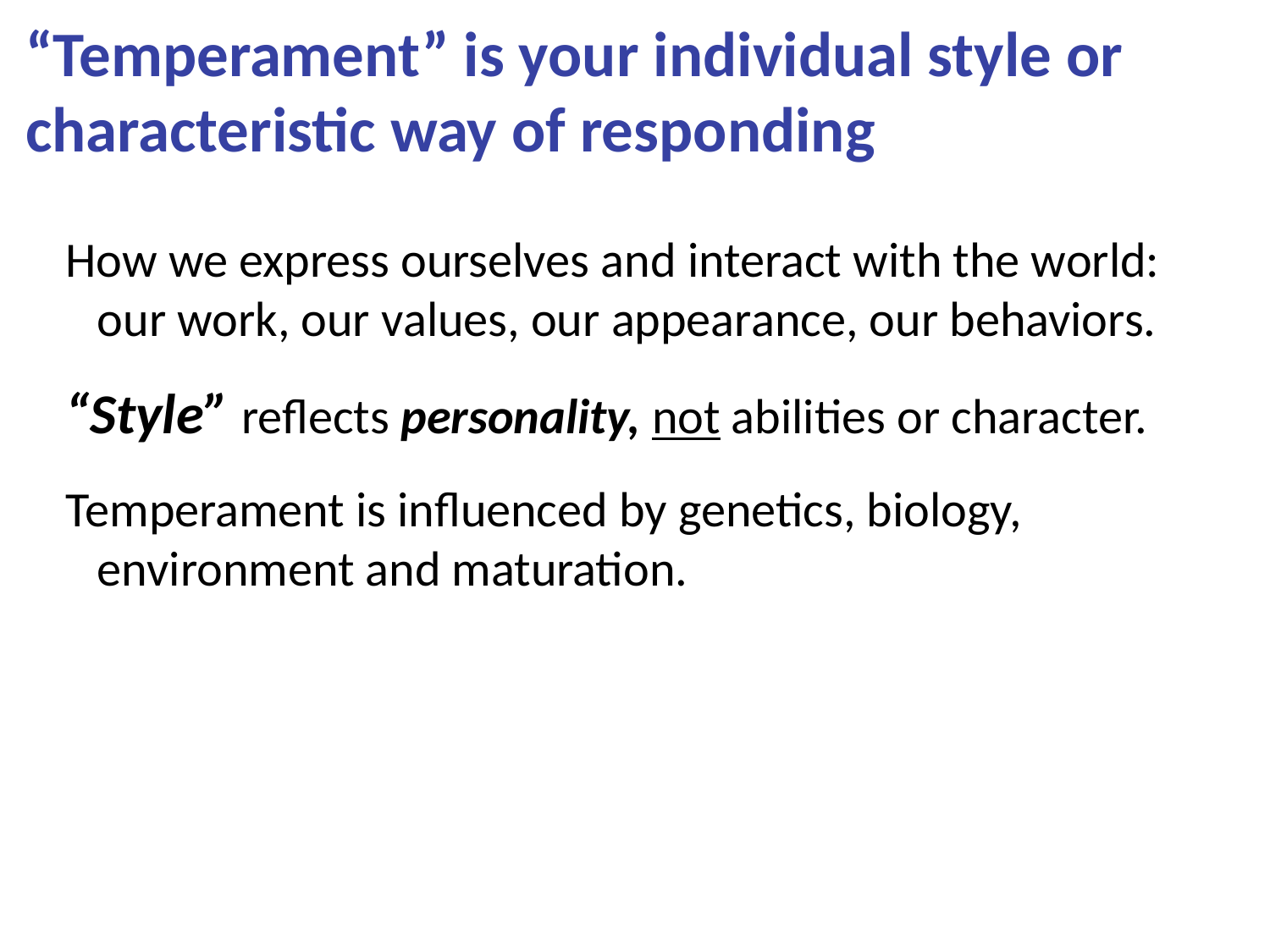

# “Temperament” is your individual style or characteristic way of responding
How we express ourselves and interact with the world: our work, our values, our appearance, our behaviors.
“Style” reflects personality, not abilities or character.
Temperament is influenced by genetics, biology, environment and maturation.

## Slide 9
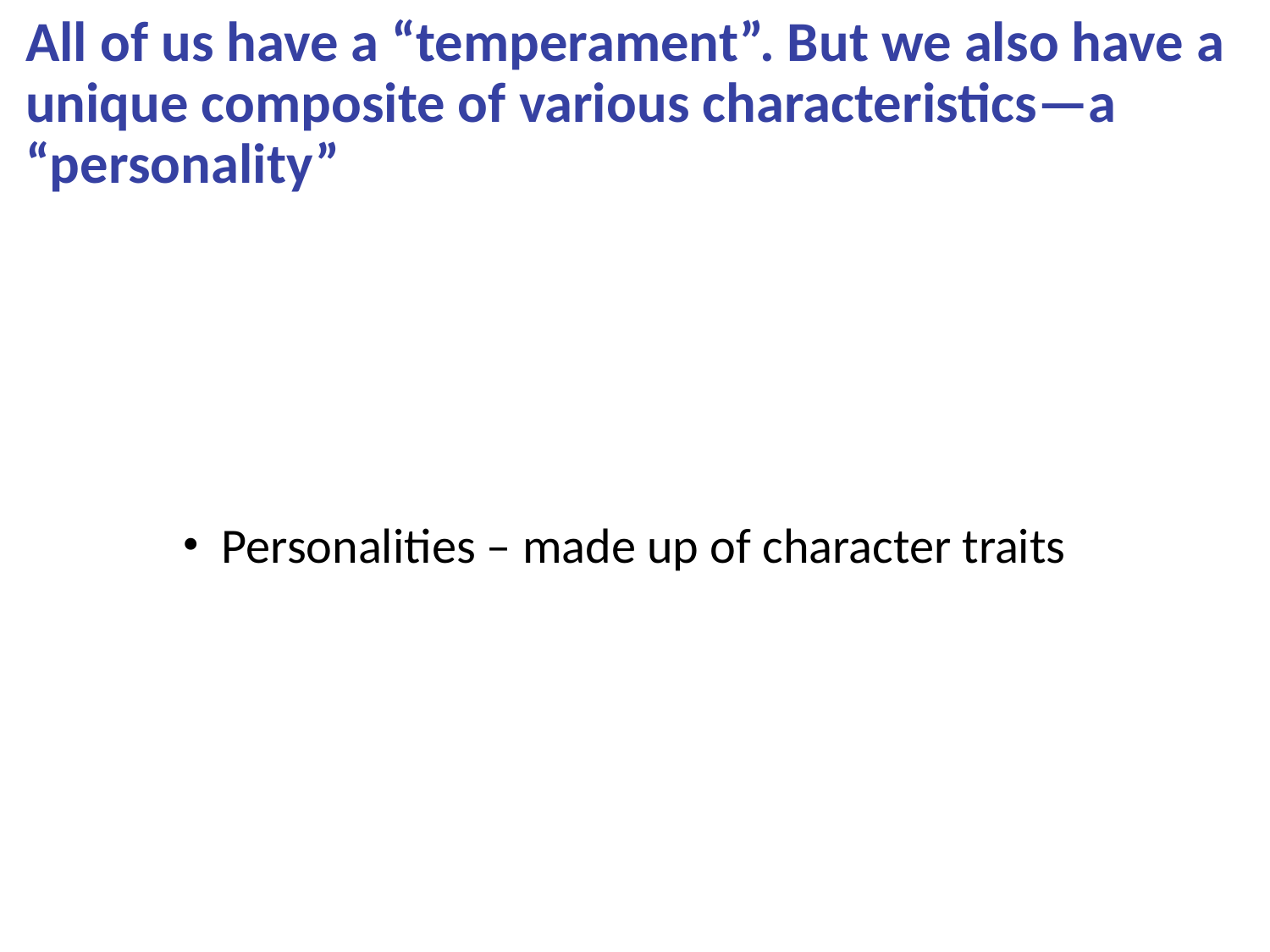

# All of us have a “temperament”. But we also have a unique composite of various characteristics—a “personality”
Personalities – made up of character traits

## Slide 10
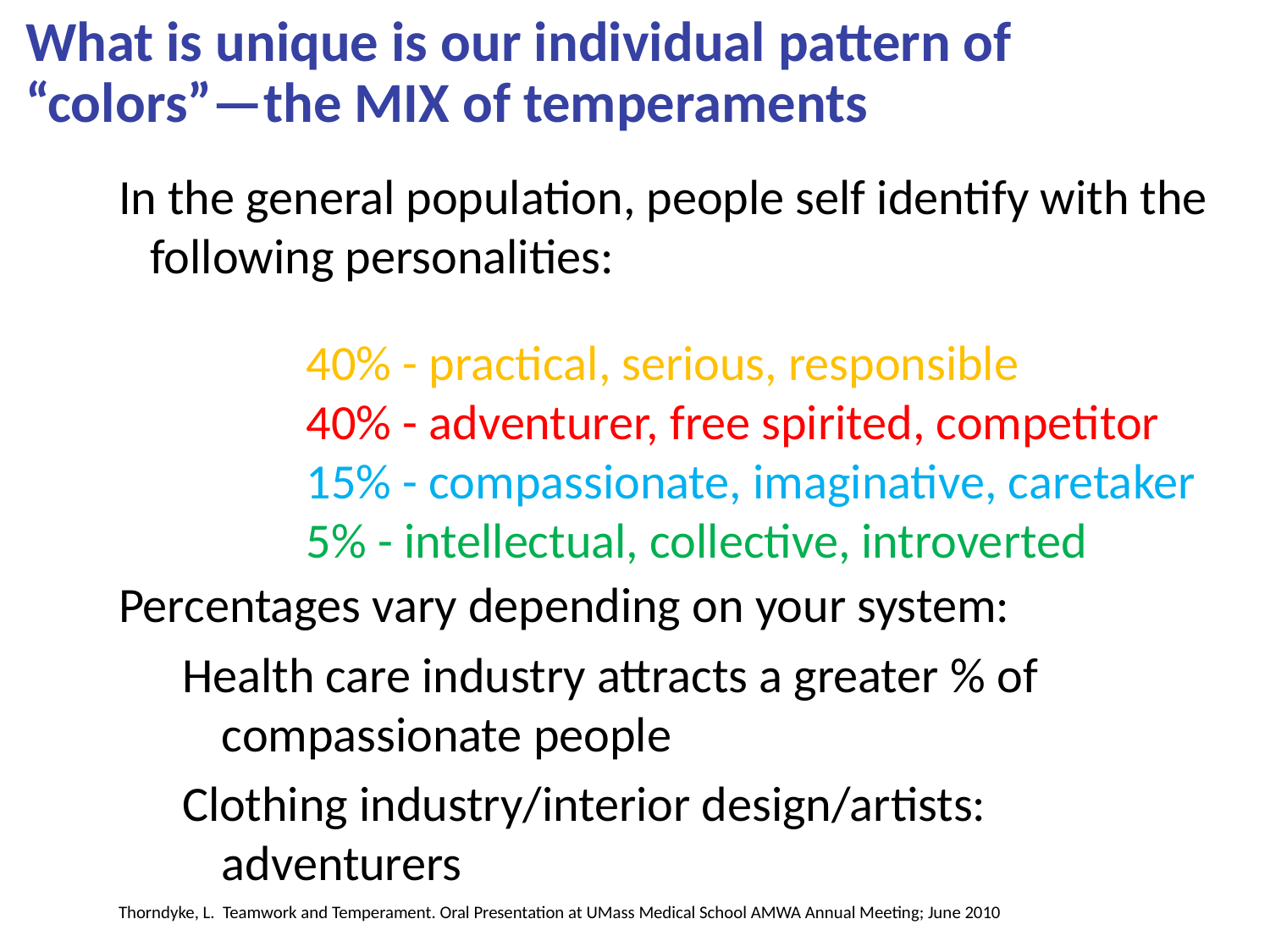

# What is unique is our individual pattern of “colors”—the MIX of temperaments
In the general population, people self identify with the following personalities:
Percentages vary depending on your system:
Health care industry attracts a greater % of compassionate people
Clothing industry/interior design/artists: adventurers
Thorndyke, L. Teamwork and Temperament. Oral Presentation at UMass Medical School AMWA Annual Meeting; June 2010
40% - practical, serious, responsible
40% - adventurer, free spirited, competitor
15% - compassionate, imaginative, caretaker
5% - intellectual, collective, introverted

## Slide 11
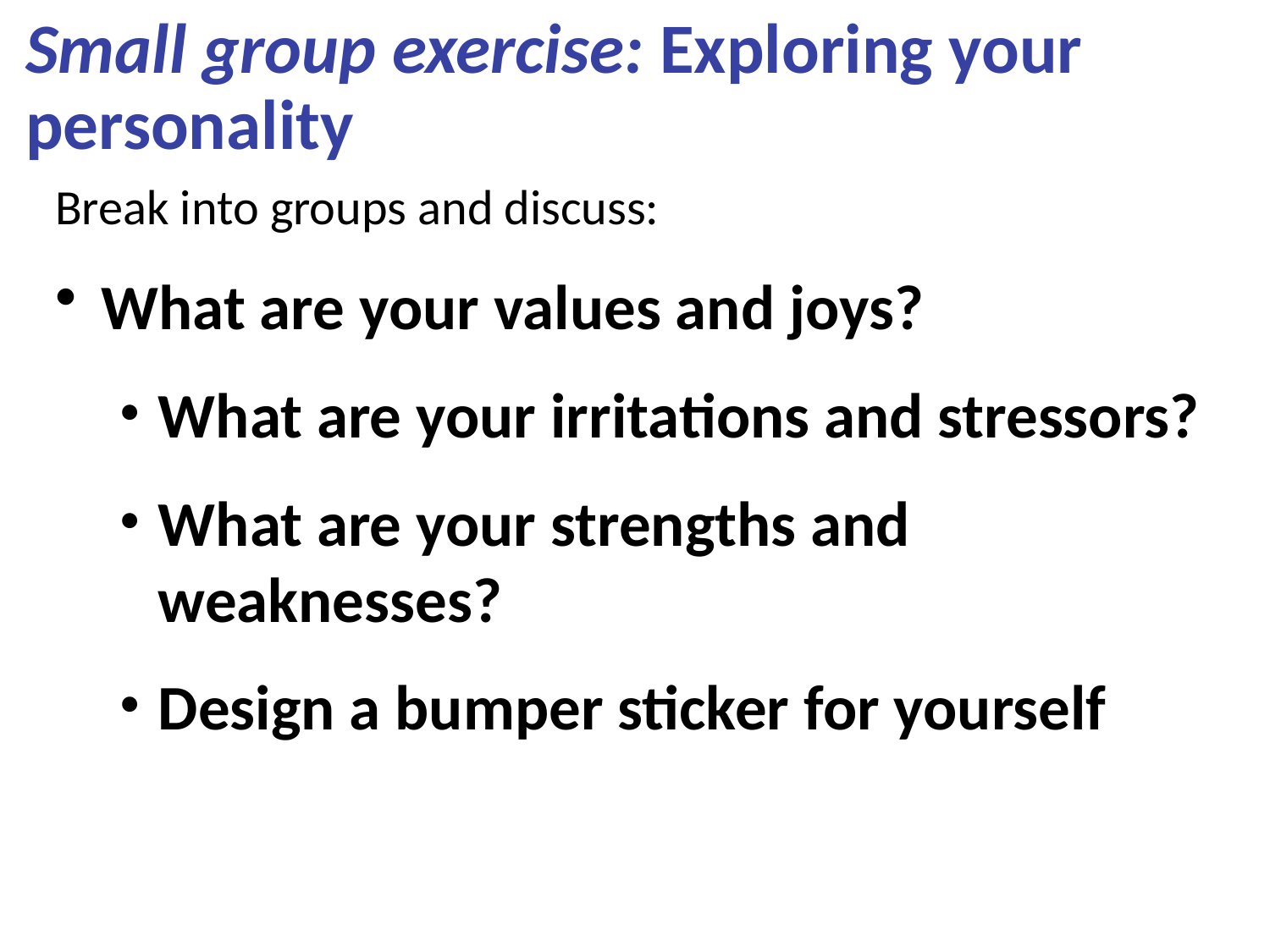

# Small group exercise: Exploring your personality
Break into groups and discuss:
 What are your values and joys?
What are your irritations and stressors?
What are your strengths and weaknesses?
Design a bumper sticker for yourself

## Slide 12
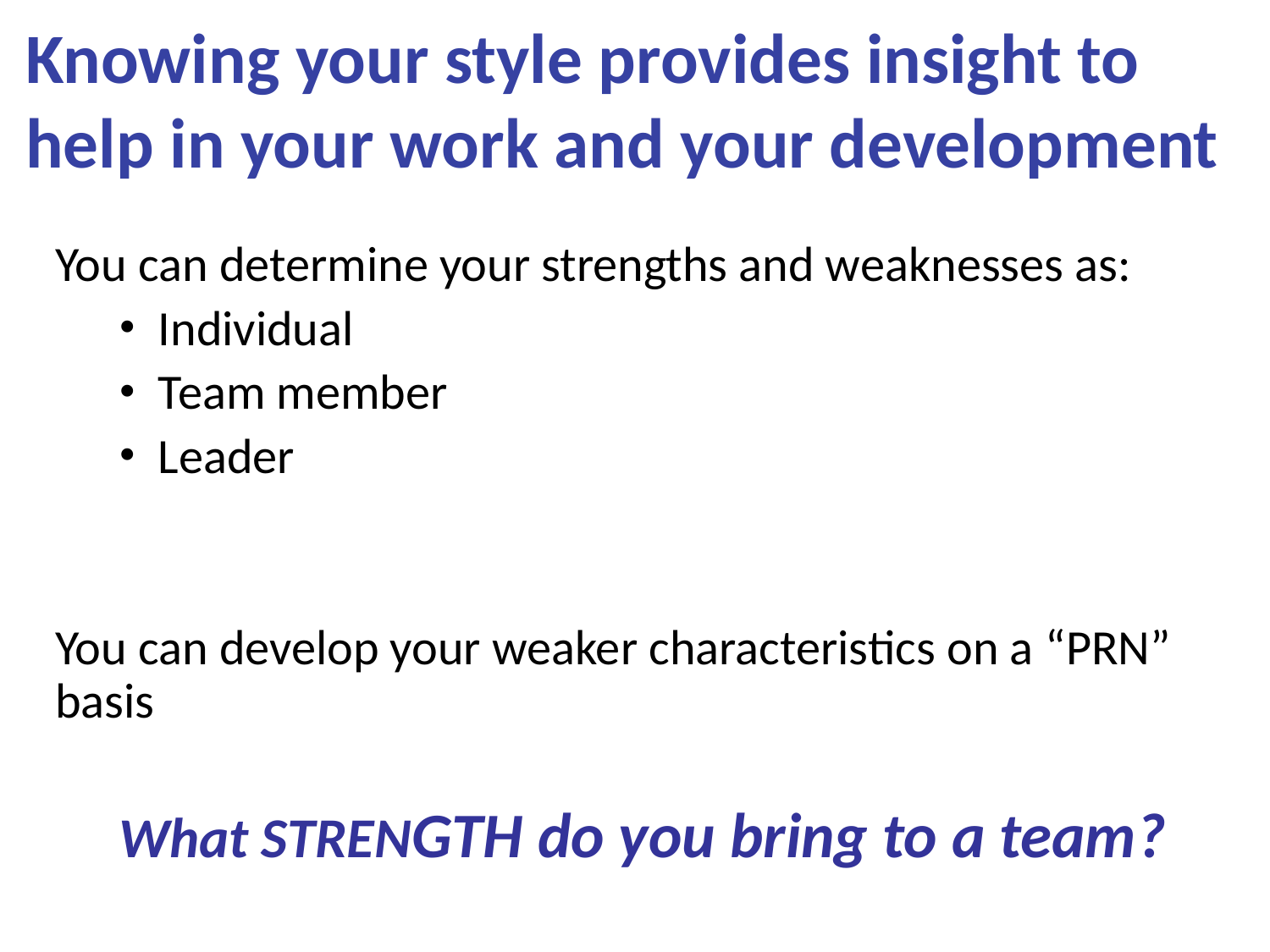

# Knowing your style provides insight to help in your work and your development
You can determine your strengths and weaknesses as:
Individual
Team member
Leader
You can develop your weaker characteristics on a “PRN” basis
What STRENGTH do you bring to a team?

## Slide 13
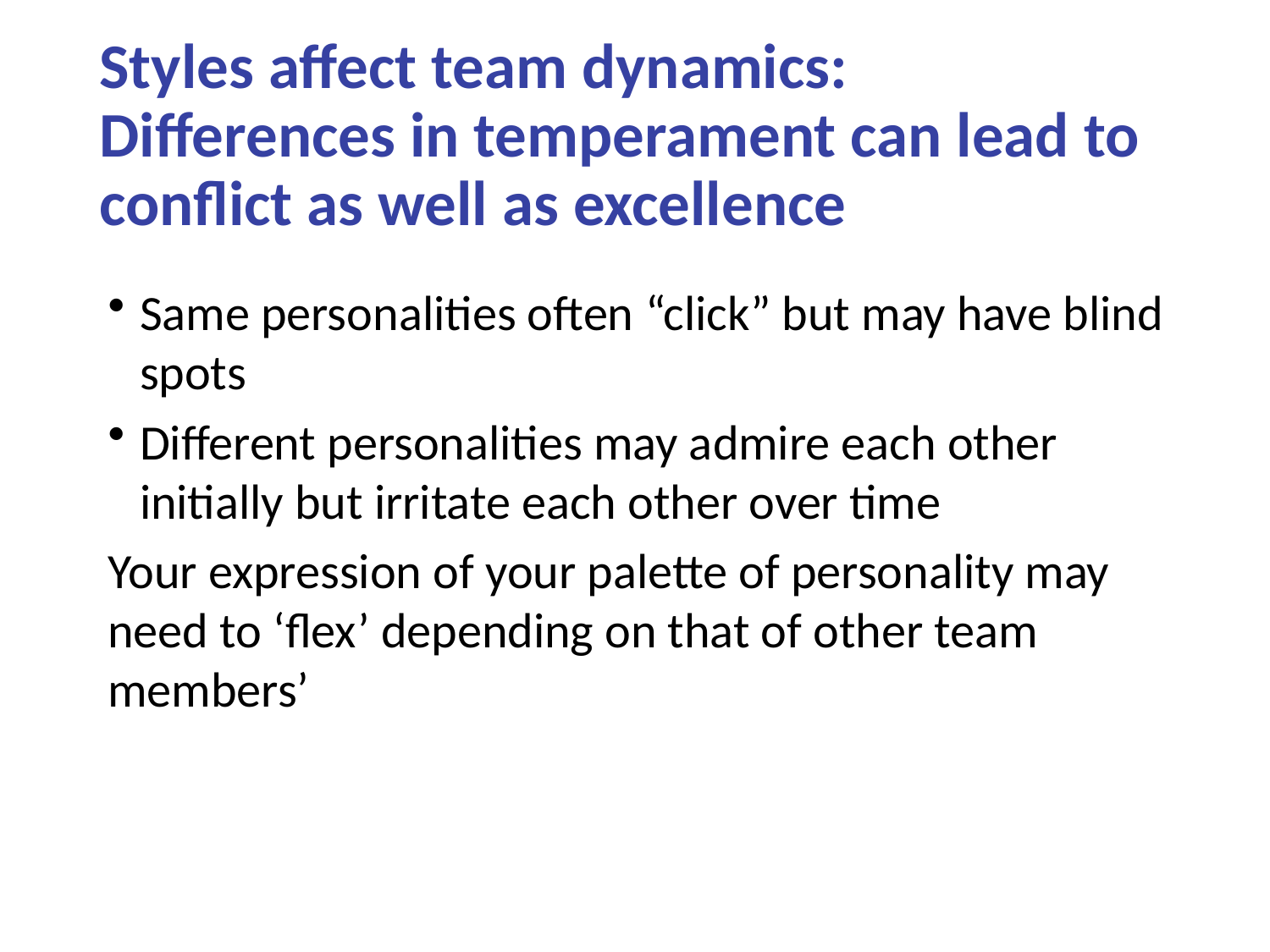

# Styles affect team dynamics: Differences in temperament can lead to conflict as well as excellence
Same personalities often “click” but may have blind spots
Different personalities may admire each other initially but irritate each other over time
Your expression of your palette of personality may need to ‘flex’ depending on that of other team members’

## Slide 14
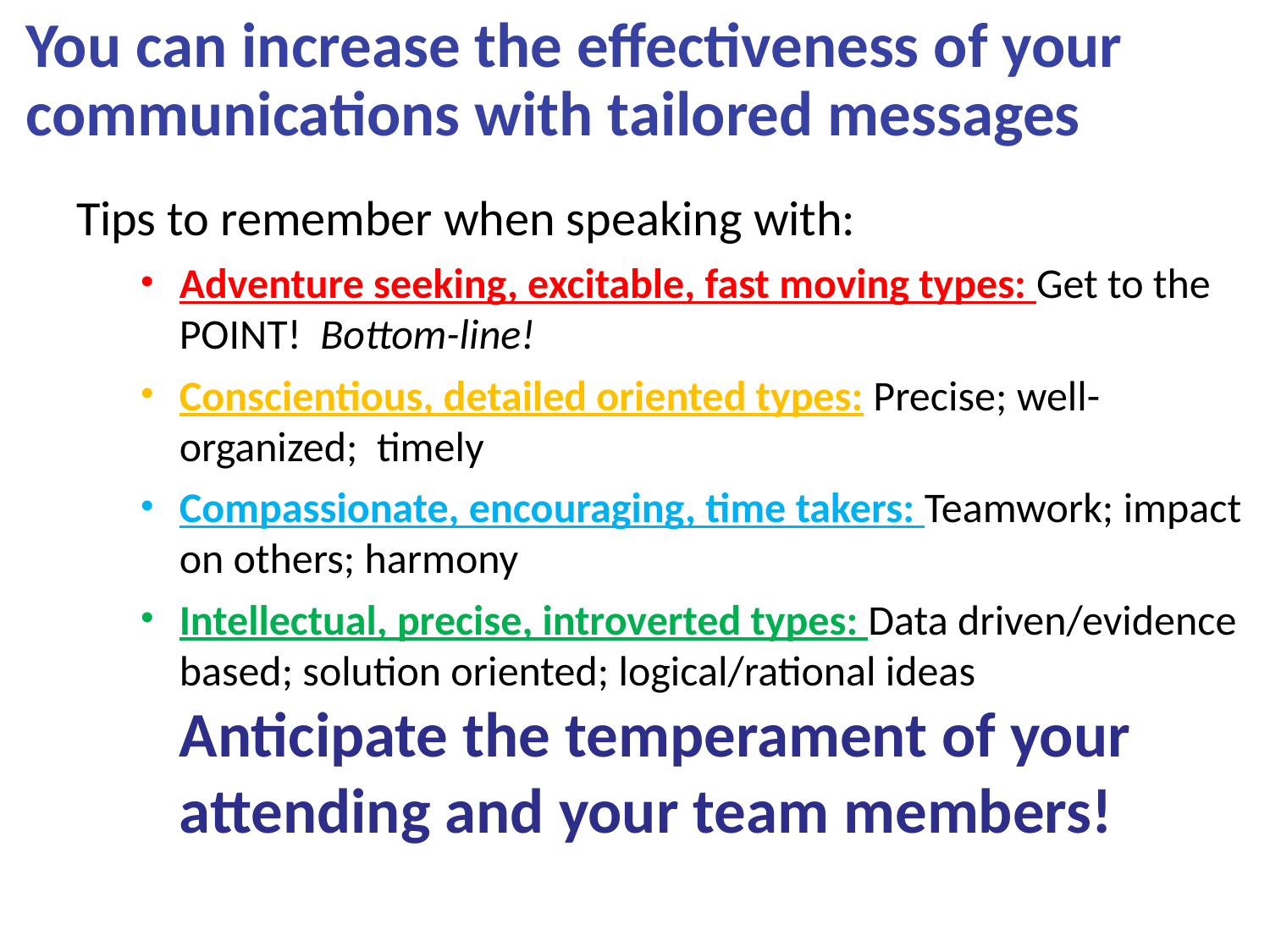

# You can increase the effectiveness of your communications with tailored messages
Tips to remember when speaking with:
Adventure seeking, excitable, fast moving types: Get to the POINT! Bottom-line!
Conscientious, detailed oriented types: Precise; well-organized; timely
Compassionate, encouraging, time takers: Teamwork; impact on others; harmony
Intellectual, precise, introverted types: Data driven/evidence based; solution oriented; logical/rational ideas
Anticipate the temperament of your attending and your team members!

## Slide 15
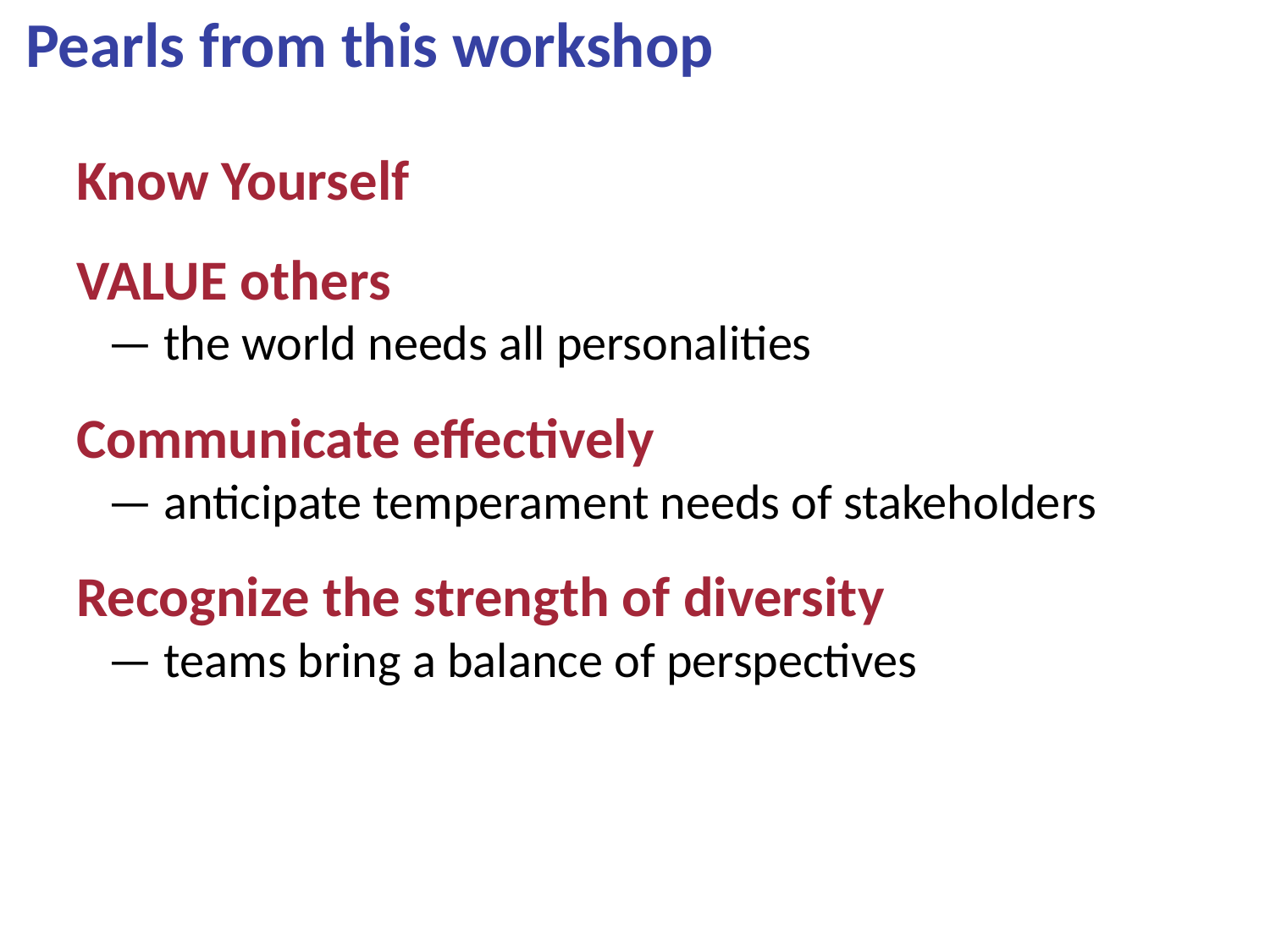

# Pearls from this workshop
Know Yourself
VALUE others
— the world needs all personalities
Communicate effectively
— anticipate temperament needs of stakeholders
Recognize the strength of diversity
— teams bring a balance of perspectives

## Slide 16
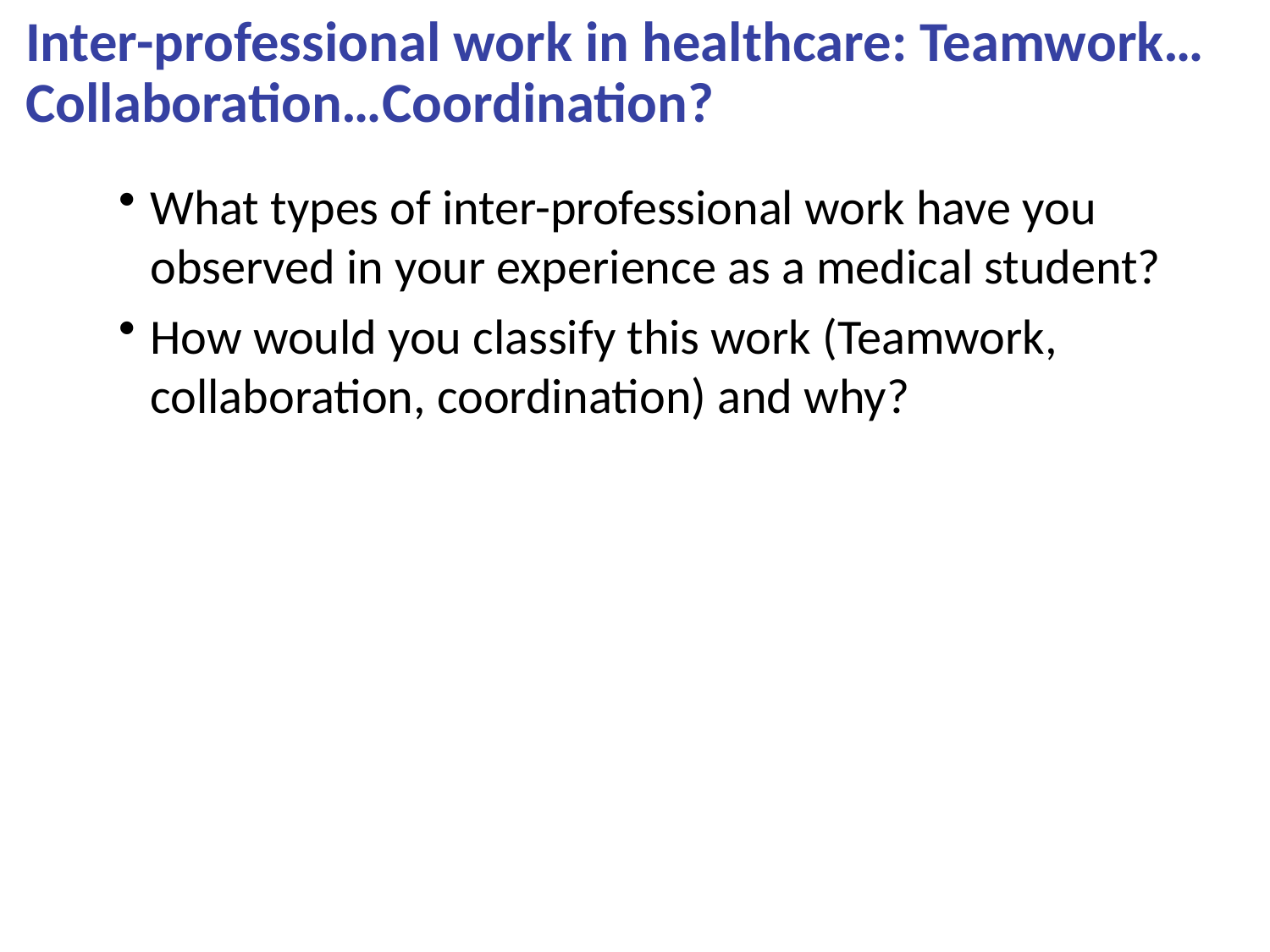

# Inter-professional work in healthcare: Teamwork…Collaboration…Coordination?
What types of inter-professional work have you observed in your experience as a medical student?
How would you classify this work (Teamwork, collaboration, coordination) and why?

## Slide 17
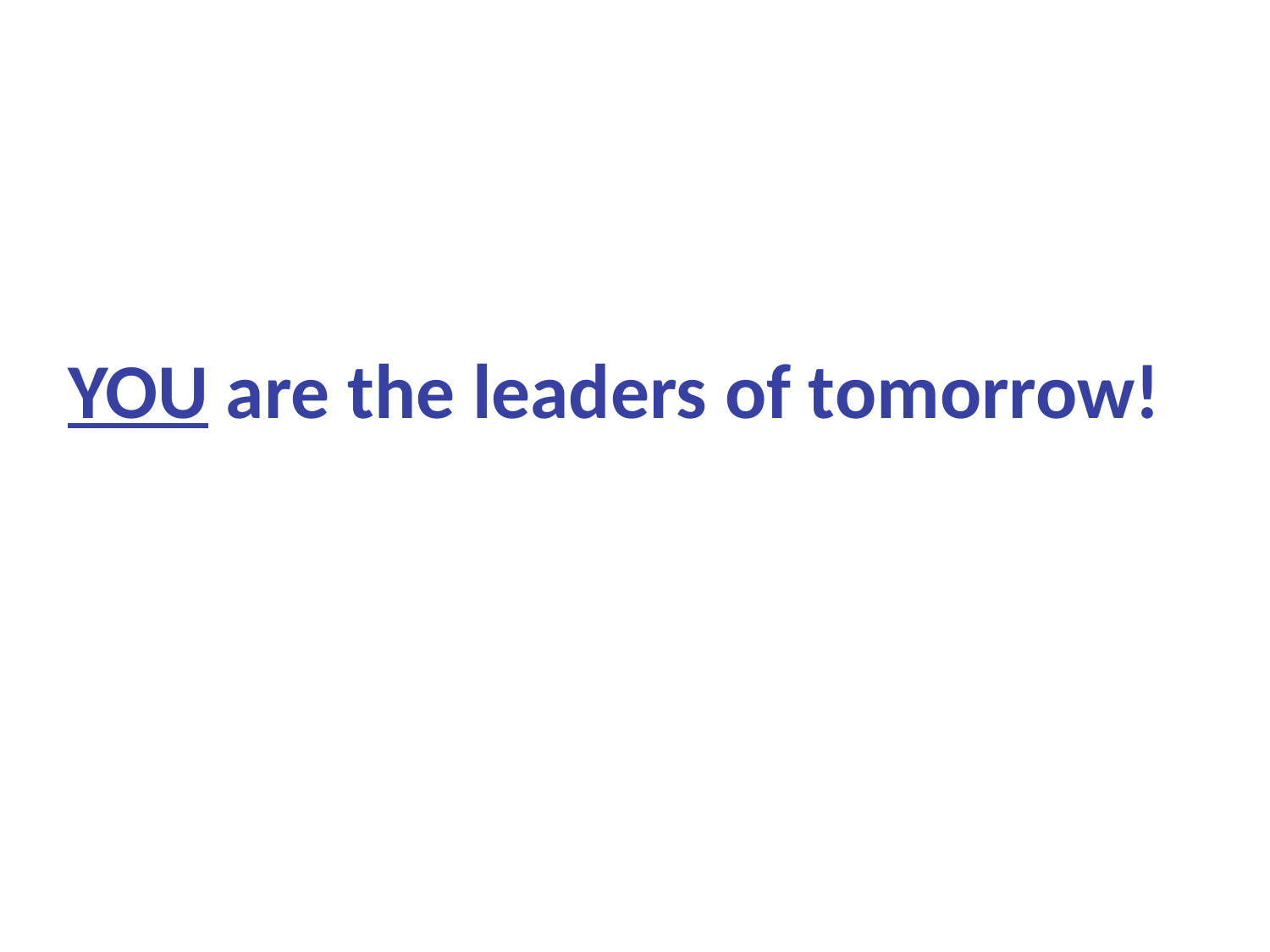

# YOU are the leaders of tomorrow!

## Slide 18
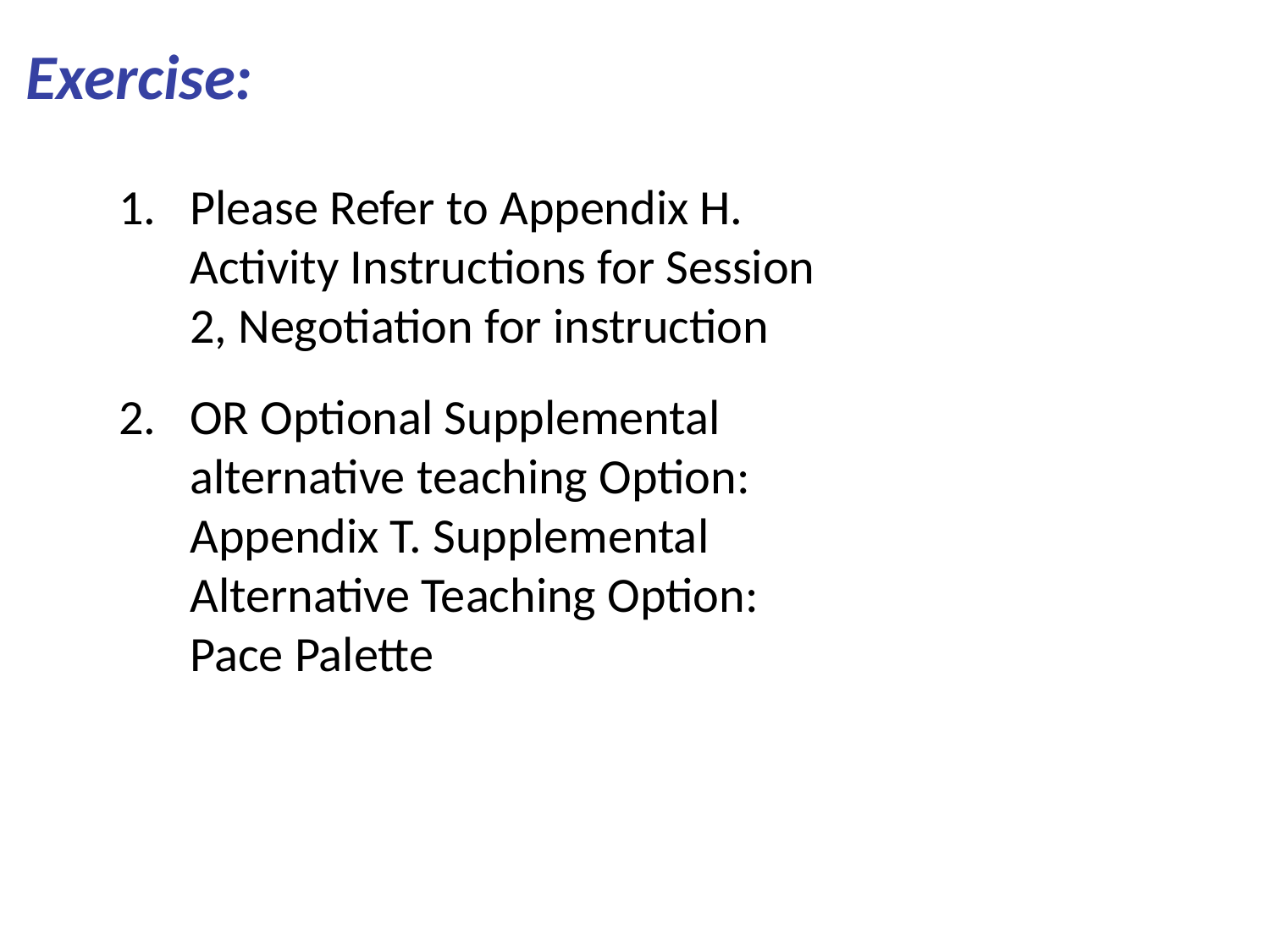

# Exercise:
Please Refer to Appendix H. Activity Instructions for Session 2, Negotiation for instruction
OR Optional Supplemental alternative teaching Option: Appendix T. Supplemental Alternative Teaching Option: Pace Palette
