# Supplementary material for: Preparing Medical Students to Be Physician Leaders: A Leadership Training Program for Students Designed and Led by Students
Source: MedEdPORTAL. 2019 Dec 13;15:10863. doi: 10.15766/mep_2374-8265.10863 (PMC7012310; doi:10.15766/mep_2374-8265.10863)
Supplement: Supplementary file 1 — A. Session 1 PPT Leadership Styles.pptx B. Session 2 PPT Teamwork.pptx C. Session 3 PPT Delegation.pptx D. Session 4 PPT Feedback.pptx E. Session 5 PPT Direction.pptx F. Session 6 Optional Review PPT Consolidation.pptx G. Session 1 Activity Instructions.docx H. Session 2 Activity Instructions.docx I. Session 3 Activity Instructions.docx J. Session 4 Activity Instructions and Figure.docx K. Session 5 Activity Instructions.docx L. Session 6 Activity Instructions.docx M. Precourse and Postcourse Evaluation.docx N. Session 1 Evaluation.docx O. Session 2 Evaluation.docx P. Session 3 Evaluation.docx Q. Session 4 Evaluation.docx R. Session 5 Evaluation.docx S. Posttraining Evaluation.docx T. Supplemental Alternative Activity - PACE Palette.docx U. Supplemental Alternative Activity - ACLS Video.docx V. Supplemental Alternative Activity - Feedback Video.docx [file mep-15-10863-s001.zip › C. Session 3 PPT Delegation.pptx]

## Slide 1
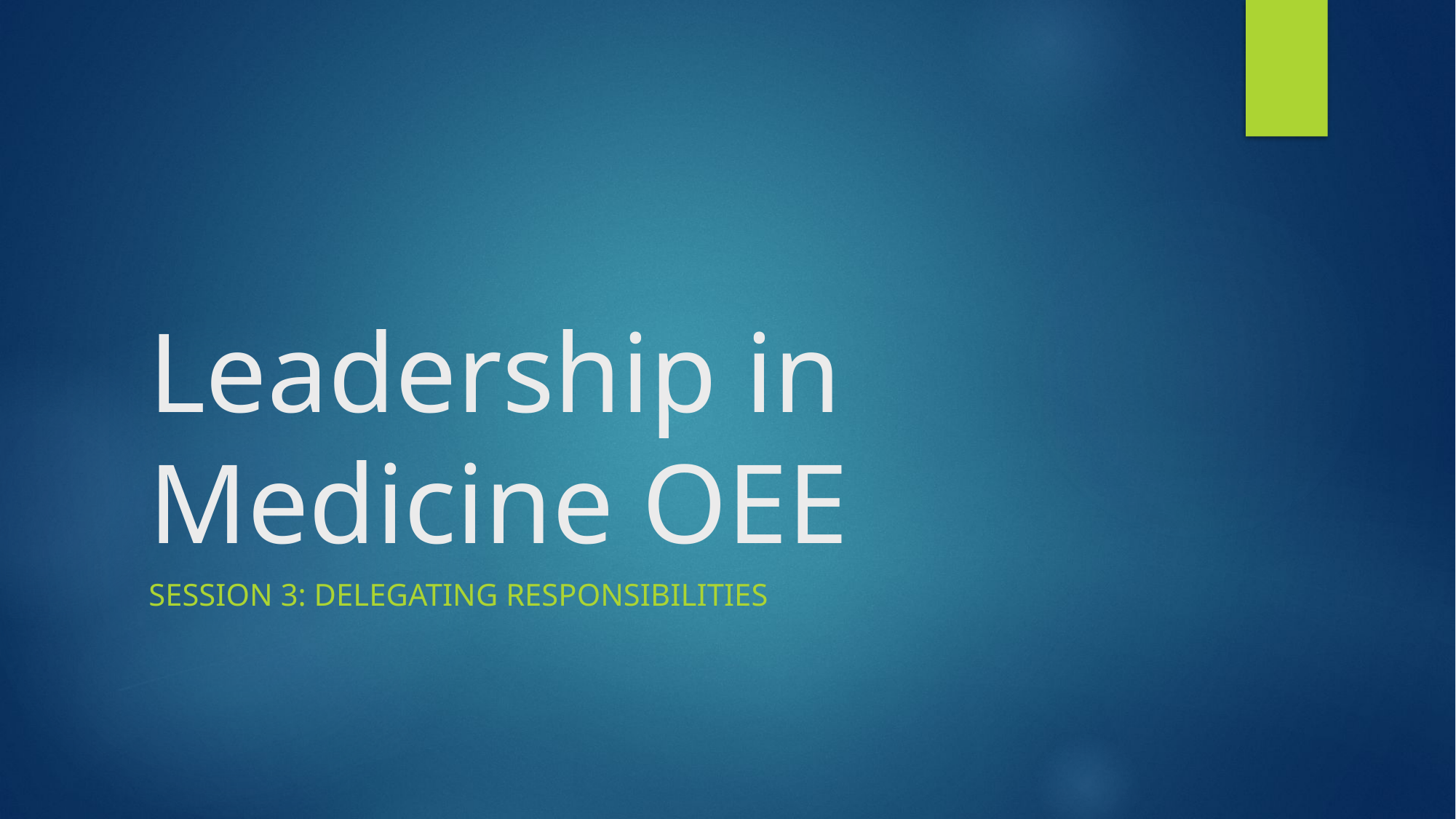

# Leadership in Medicine OEE
Session 3: Delegating Responsibilities

## Slide 2
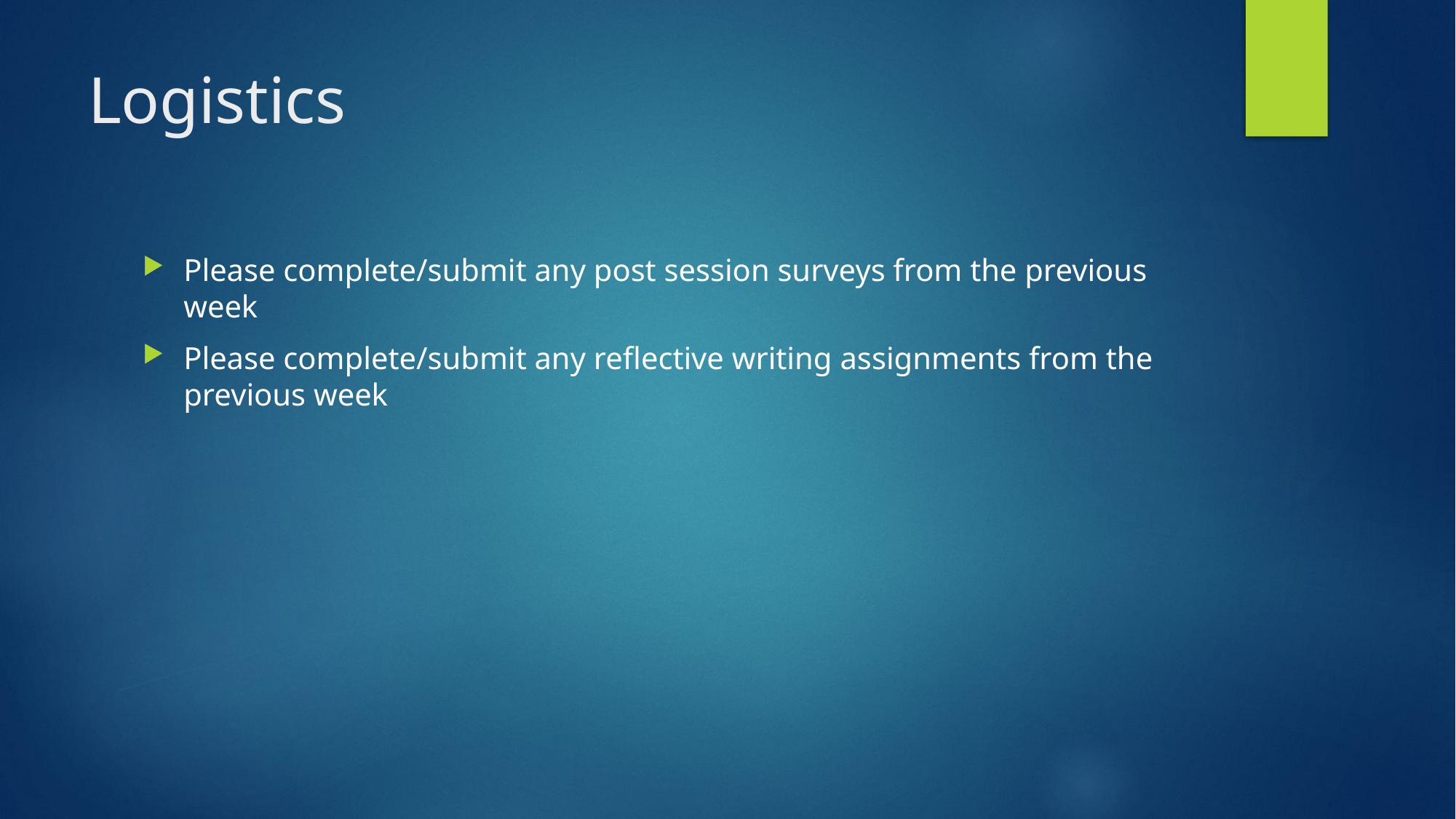

# Logistics
Please complete/submit any post session surveys from the previous week
Please complete/submit any reflective writing assignments from the previous week

## Slide 3
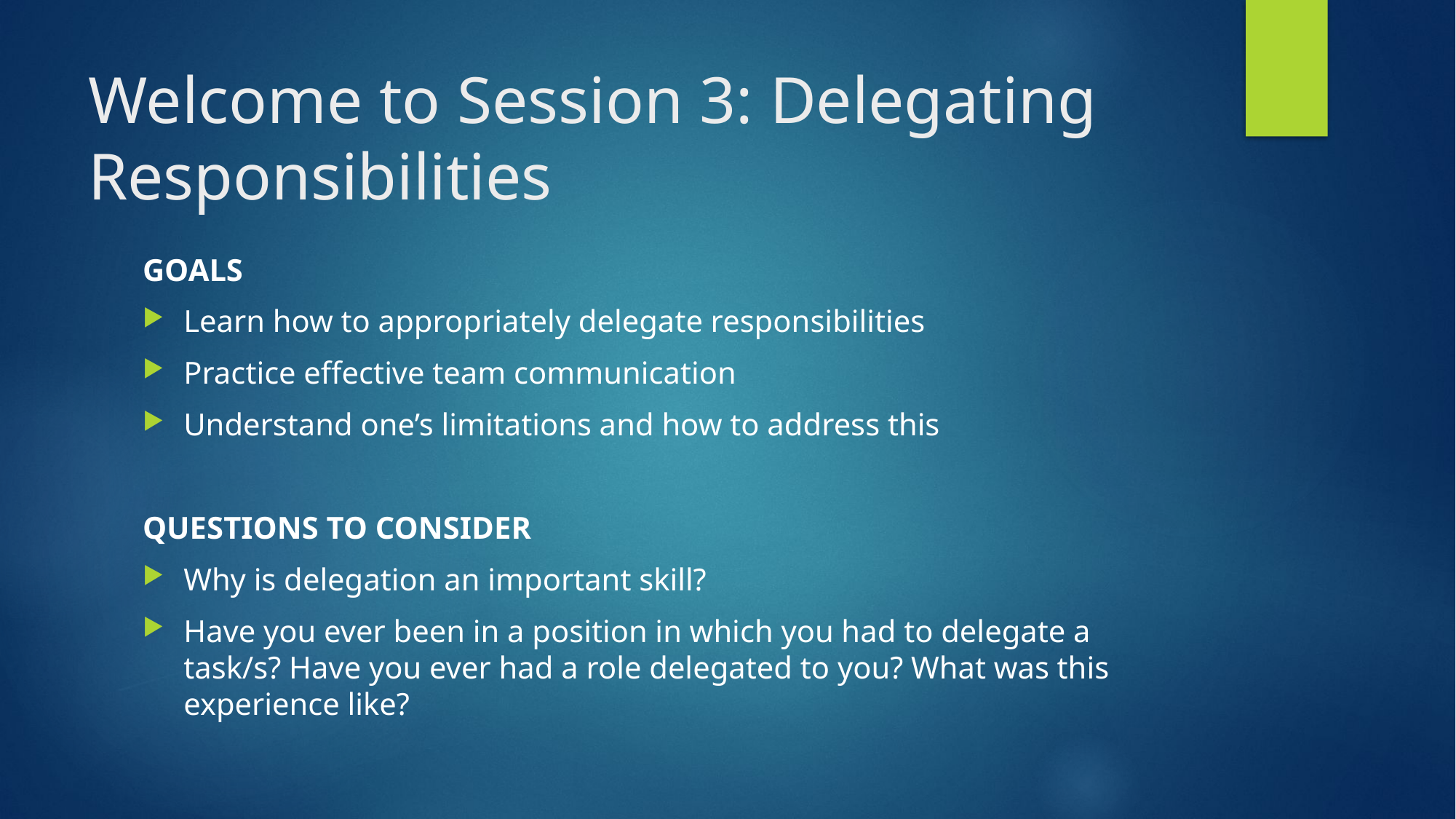

# Welcome to Session 3: Delegating Responsibilities
GOALS
Learn how to appropriately delegate responsibilities
Practice effective team communication
Understand one’s limitations and how to address this
QUESTIONS TO CONSIDER
Why is delegation an important skill?
Have you ever been in a position in which you had to delegate a task/s? Have you ever had a role delegated to you? What was this experience like?

## Slide 4
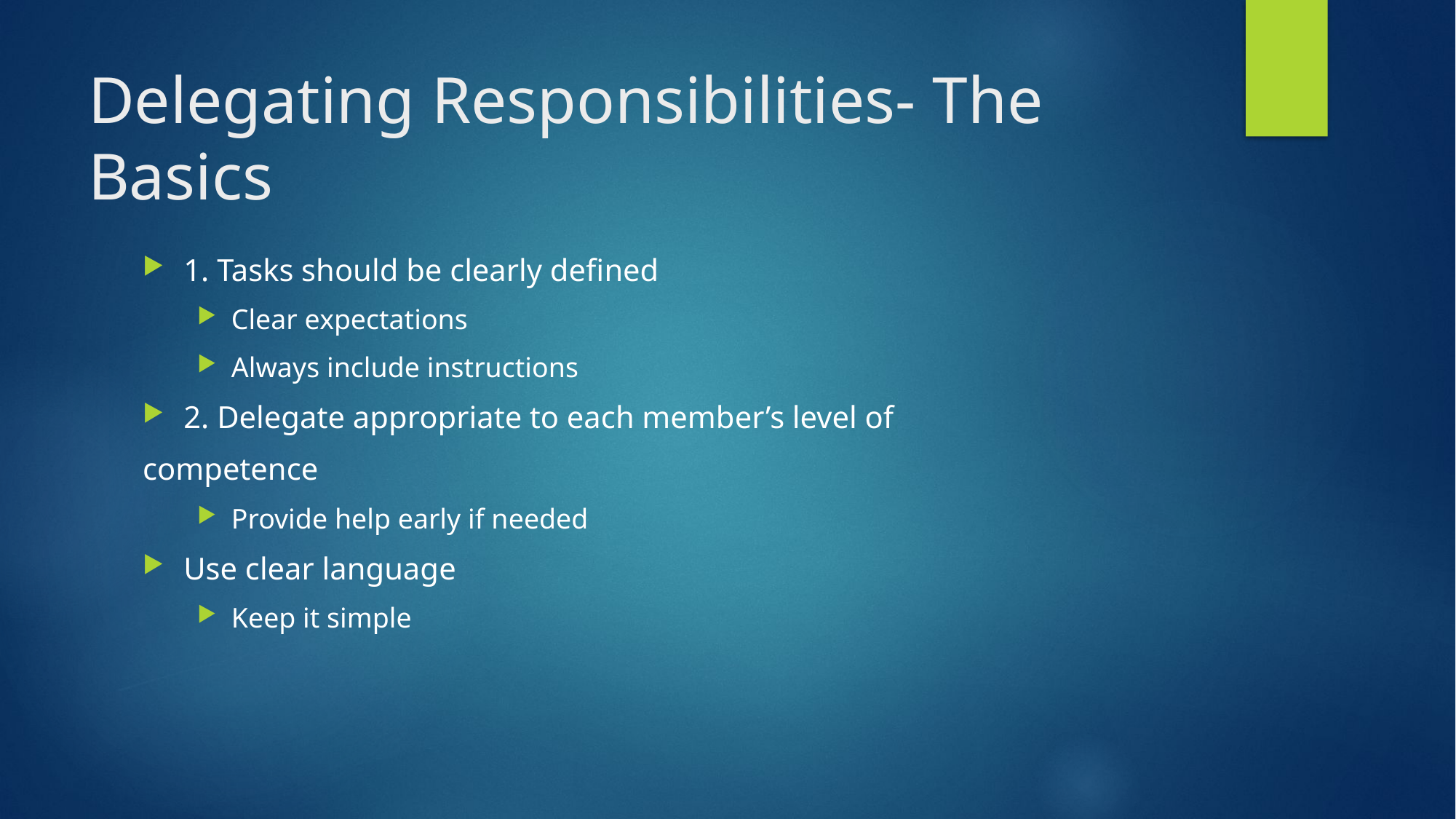

# Delegating Responsibilities- The Basics
1. Tasks should be clearly defined
Clear expectations
Always include instructions
2. Delegate appropriate to each member’s level of
competence
Provide help early if needed
Use clear language
Keep it simple

## Slide 5
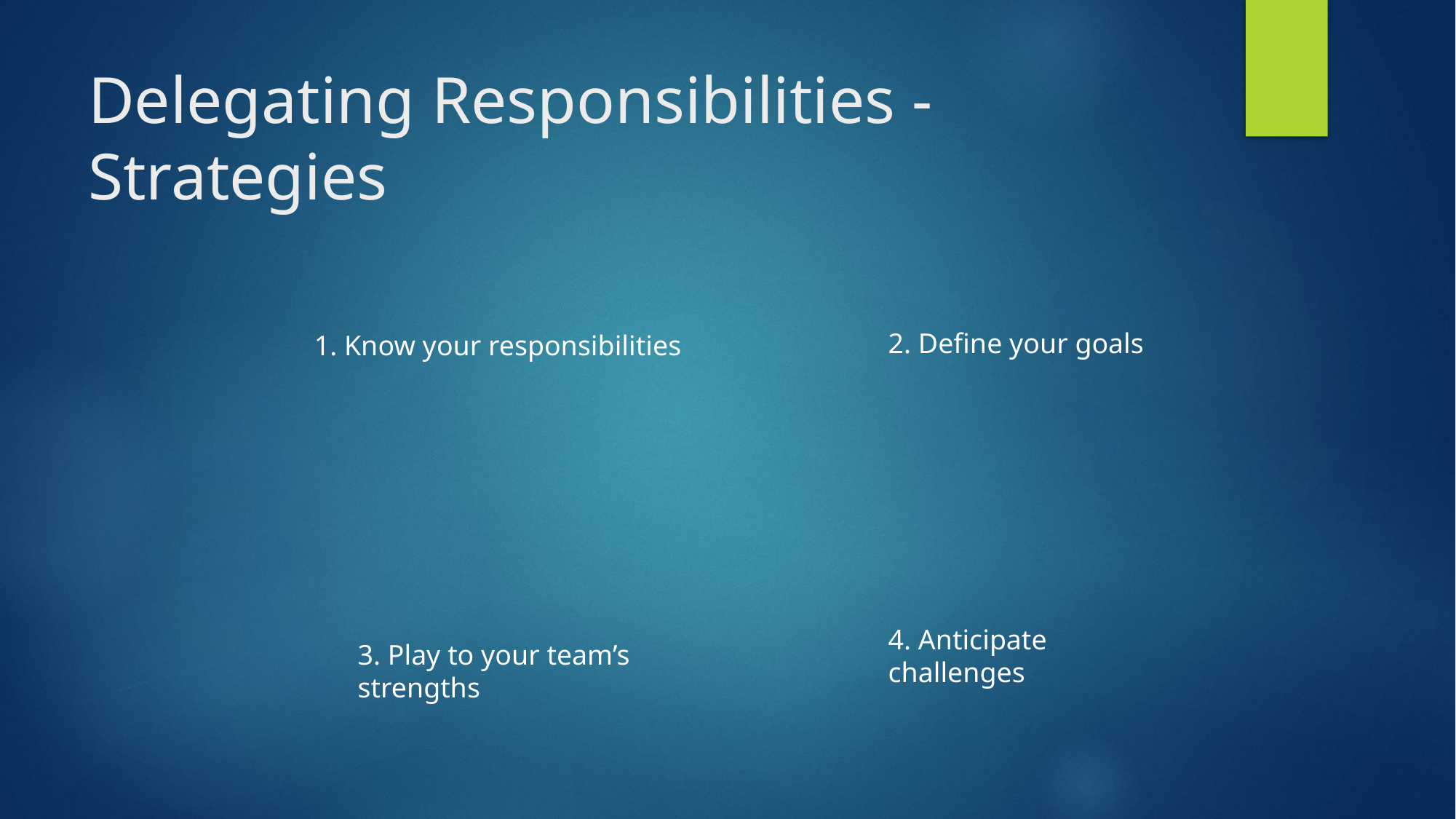

# Delegating Responsibilities - Strategies
2. Define your goals
1. Know your responsibilities
4. Anticipate challenges
3. Play to your team’s strengths

## Slide 6
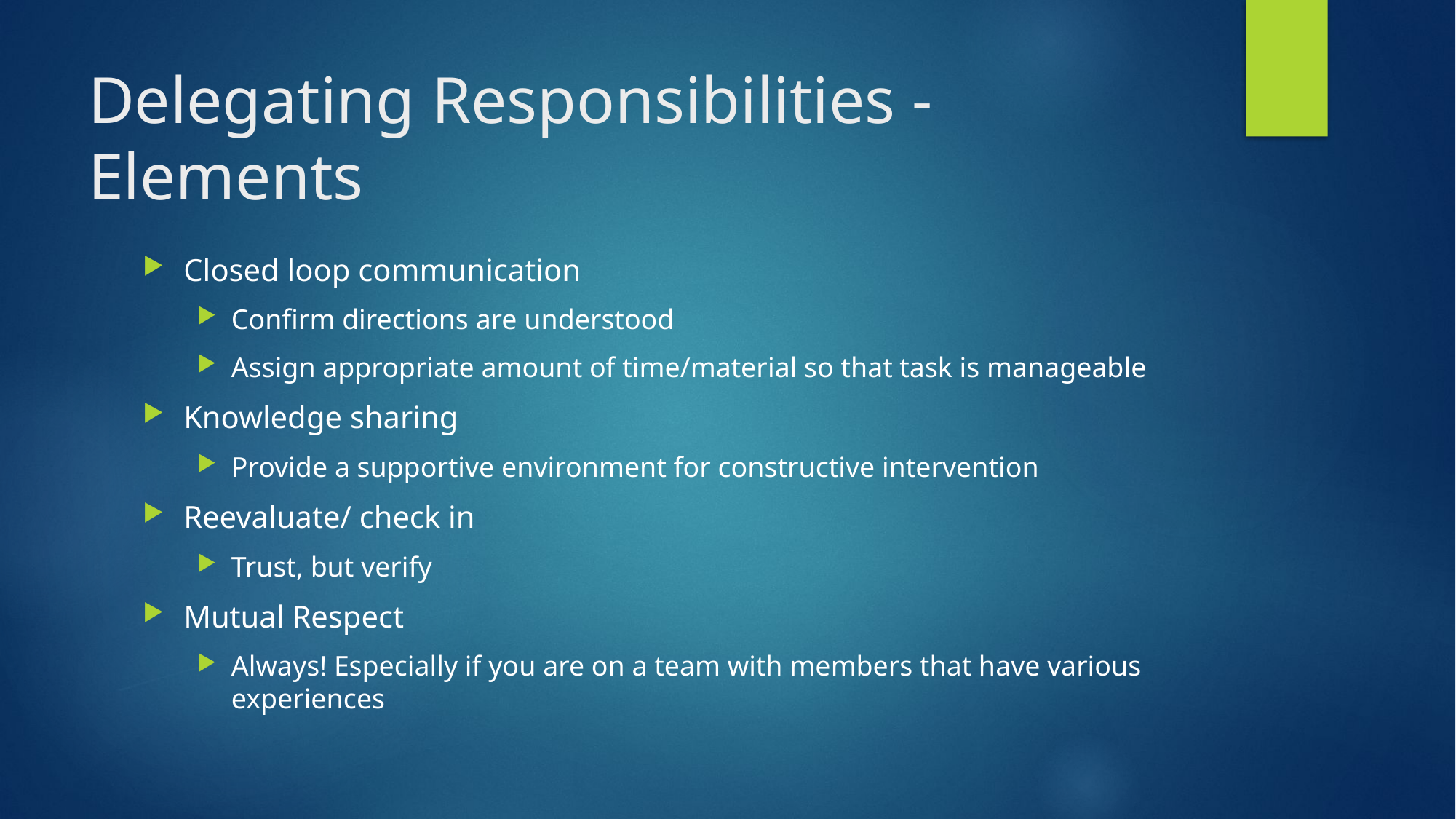

# Delegating Responsibilities - Elements
Closed loop communication
Confirm directions are understood
Assign appropriate amount of time/material so that task is manageable
Knowledge sharing
Provide a supportive environment for constructive intervention
Reevaluate/ check in
Trust, but verify
Mutual Respect
Always! Especially if you are on a team with members that have various experiences

## Slide 7
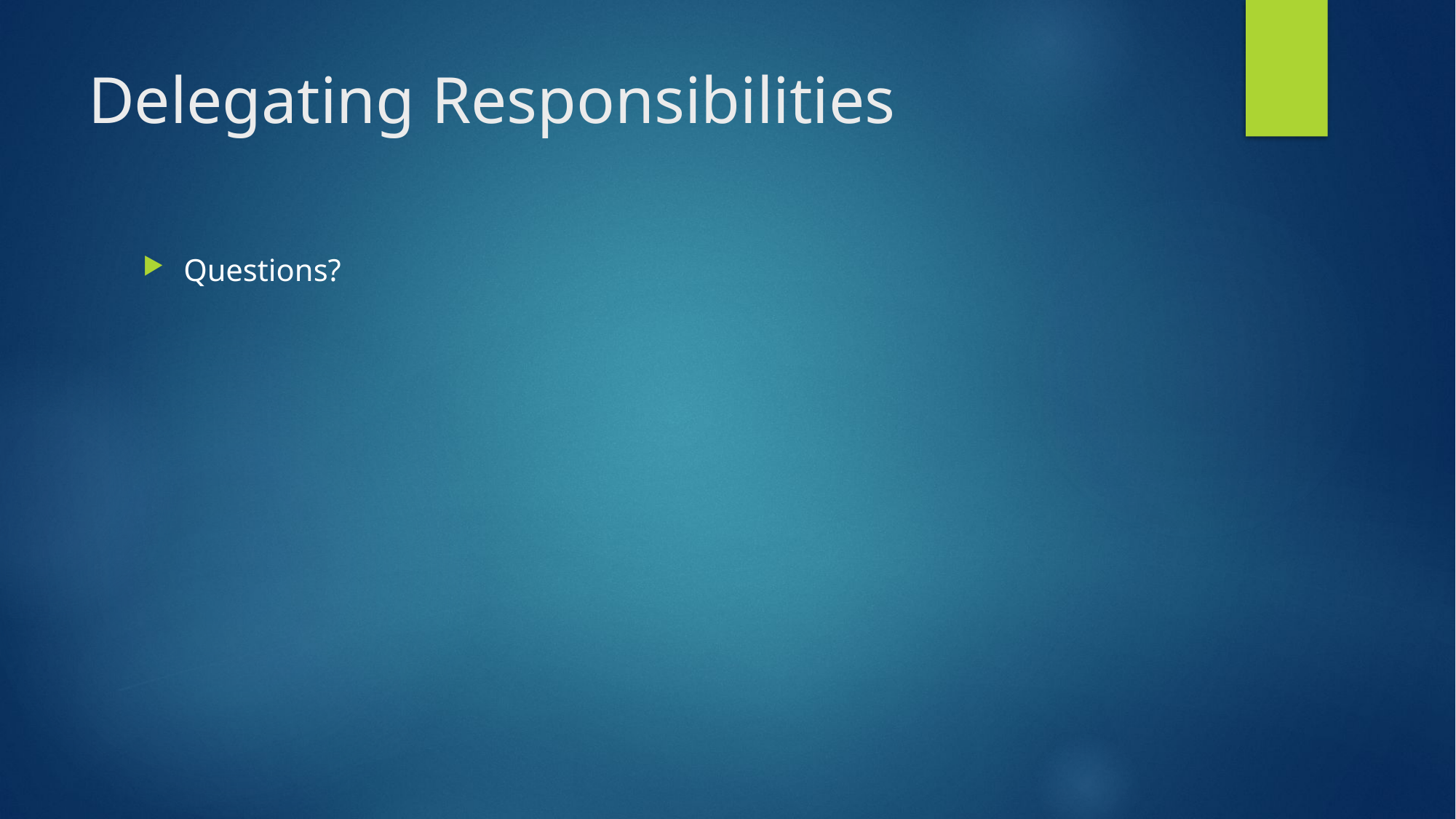

# Delegating Responsibilities
Questions?

## Slide 8
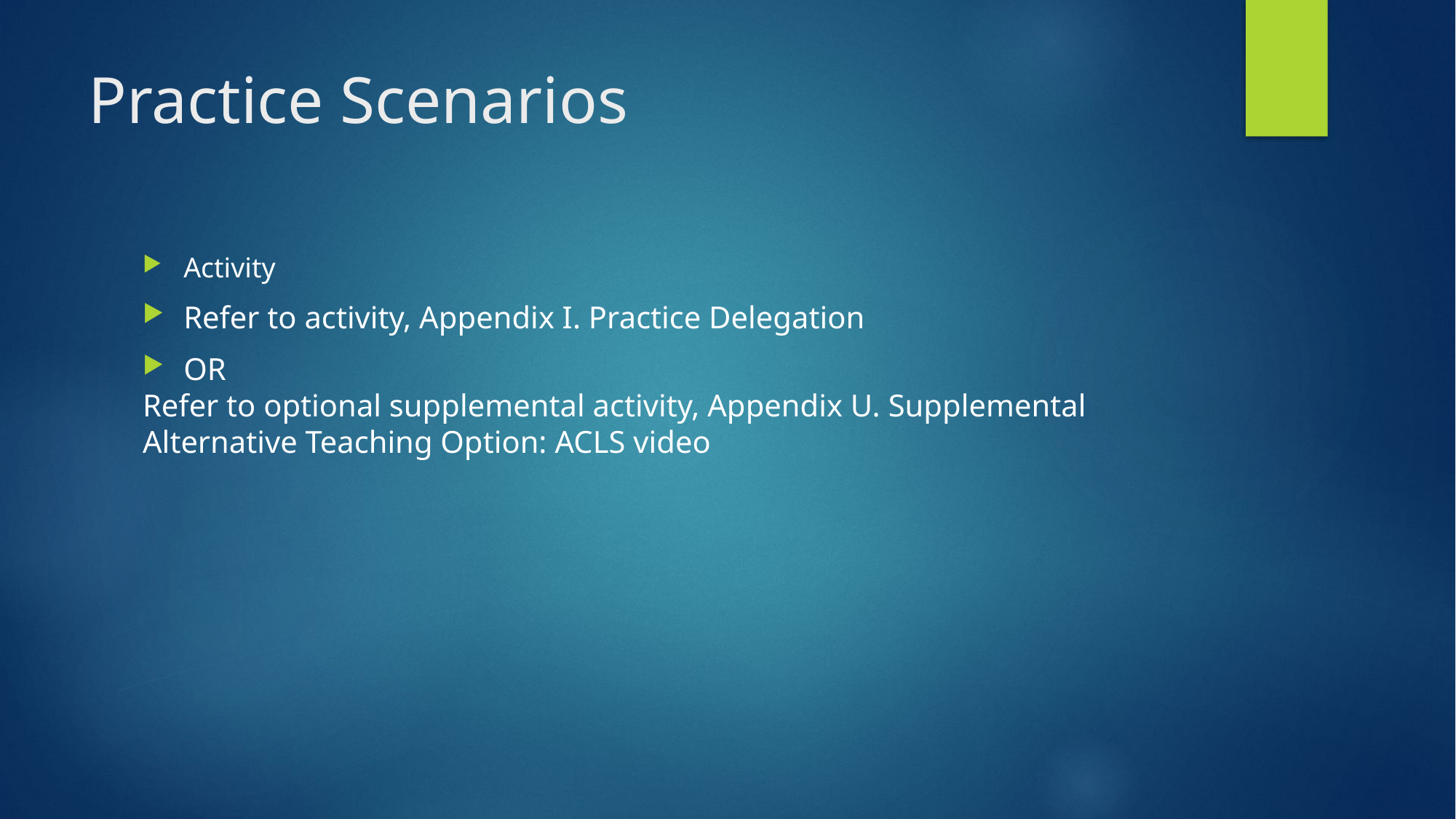

# Practice Scenarios
Activity
Refer to activity, Appendix I. Practice Delegation
OR
Refer to optional supplemental activity, Appendix U. Supplemental Alternative Teaching Option: ACLS video

## Slide 9
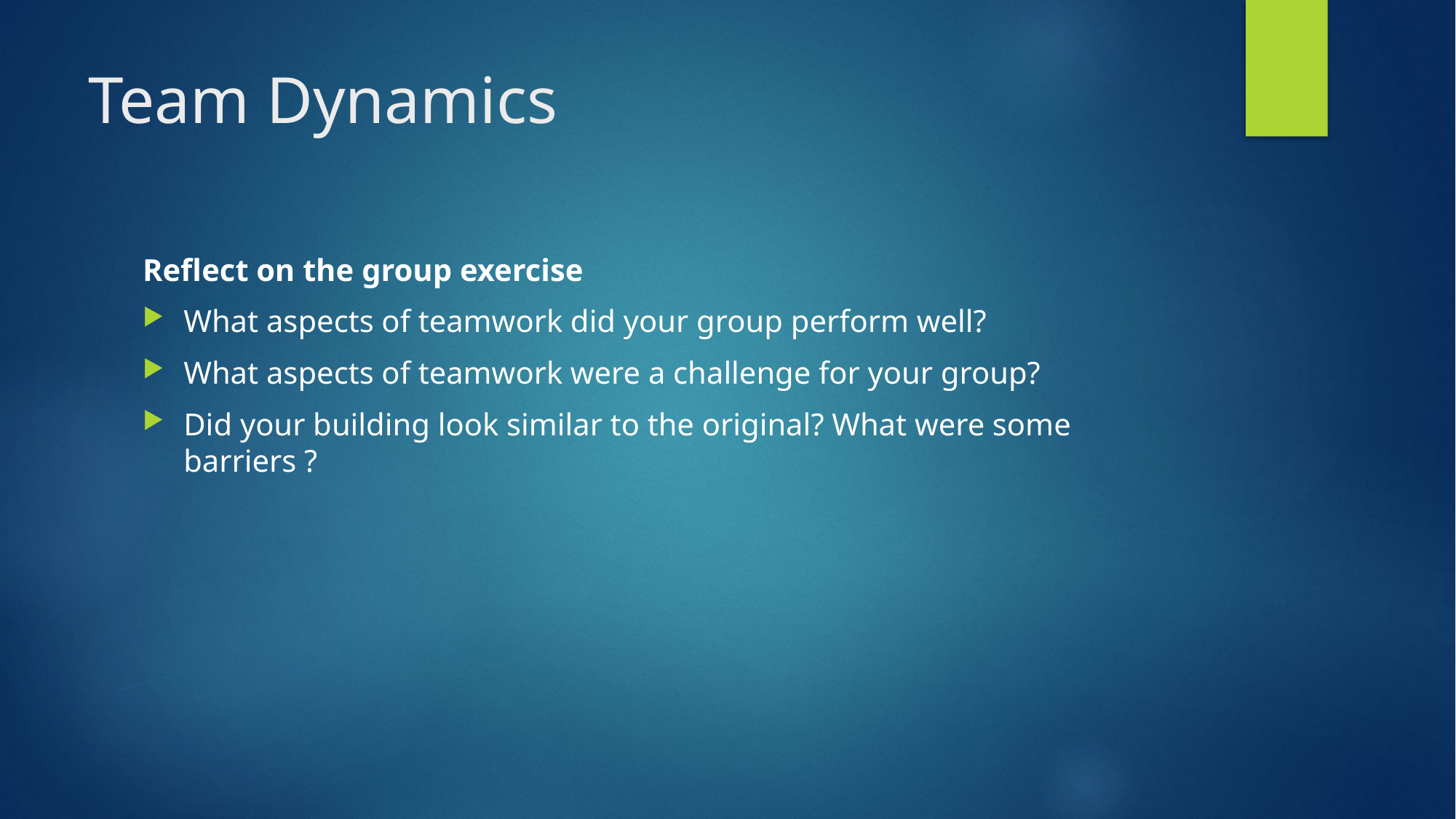

# Team Dynamics
Reflect on the group exercise
What aspects of teamwork did your group perform well?
What aspects of teamwork were a challenge for your group?
Did your building look similar to the original? What were some barriers ?
