# Supplementary material for: Preparing Medical Students to Be Physician Leaders: A Leadership Training Program for Students Designed and Led by Students
Source: MedEdPORTAL. 2019 Dec 13;15:10863. doi: 10.15766/mep_2374-8265.10863 (PMC7012310; doi:10.15766/mep_2374-8265.10863)
Supplement: Supplementary file 1 — A. Session 1 PPT Leadership Styles.pptx B. Session 2 PPT Teamwork.pptx C. Session 3 PPT Delegation.pptx D. Session 4 PPT Feedback.pptx E. Session 5 PPT Direction.pptx F. Session 6 Optional Review PPT Consolidation.pptx G. Session 1 Activity Instructions.docx H. Session 2 Activity Instructions.docx I. Session 3 Activity Instructions.docx J. Session 4 Activity Instructions and Figure.docx K. Session 5 Activity Instructions.docx L. Session 6 Activity Instructions.docx M. Precourse and Postcourse Evaluation.docx N. Session 1 Evaluation.docx O. Session 2 Evaluation.docx P. Session 3 Evaluation.docx Q. Session 4 Evaluation.docx R. Session 5 Evaluation.docx S. Posttraining Evaluation.docx T. Supplemental Alternative Activity - PACE Palette.docx U. Supplemental Alternative Activity - ACLS Video.docx V. Supplemental Alternative Activity - Feedback Video.docx [file mep-15-10863-s001.zip › D. Session 4 PPT Feedback.pptx]

## Slide 1
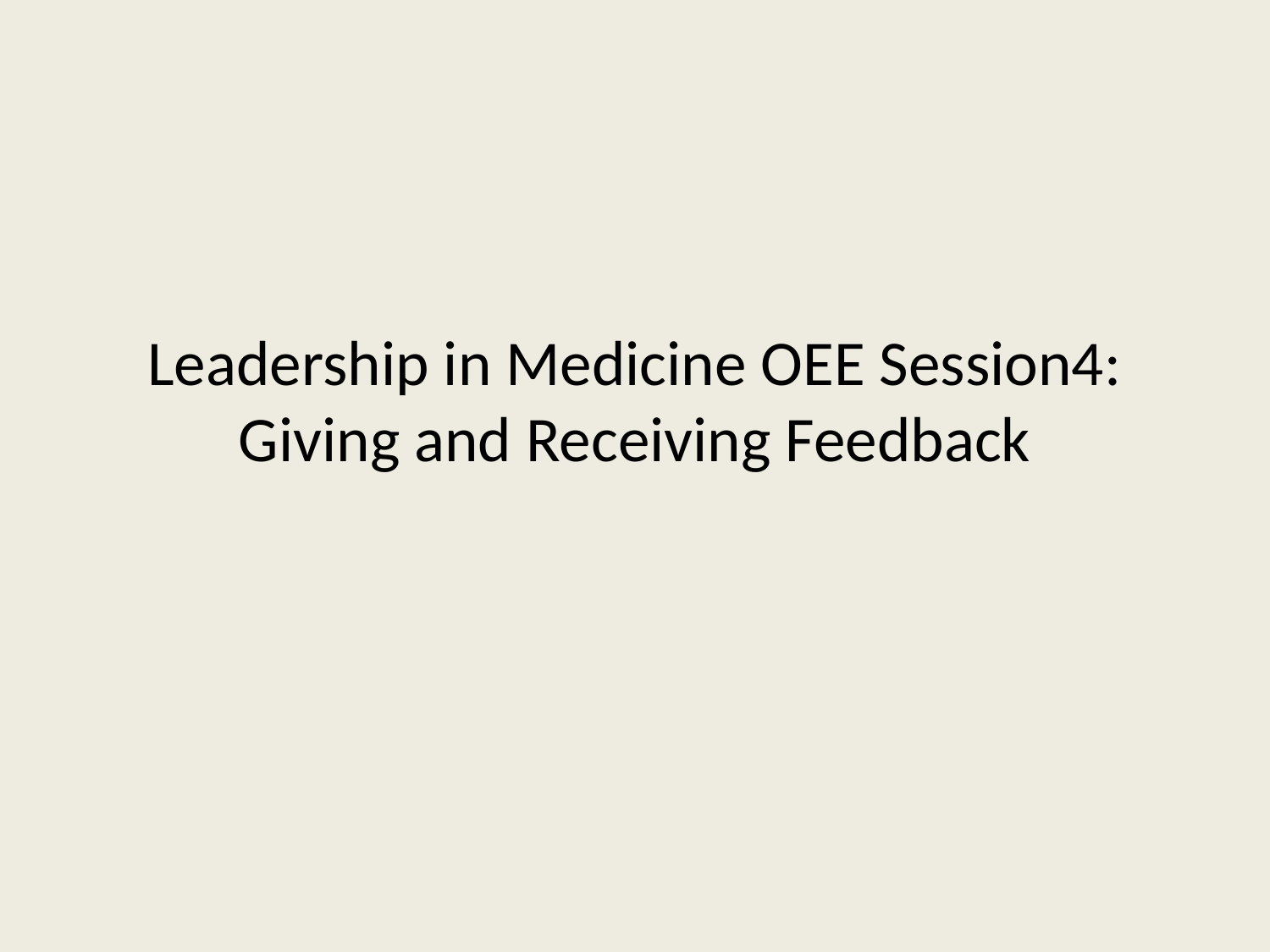

# Leadership in Medicine OEE Session4: Giving and Receiving Feedback

## Slide 2
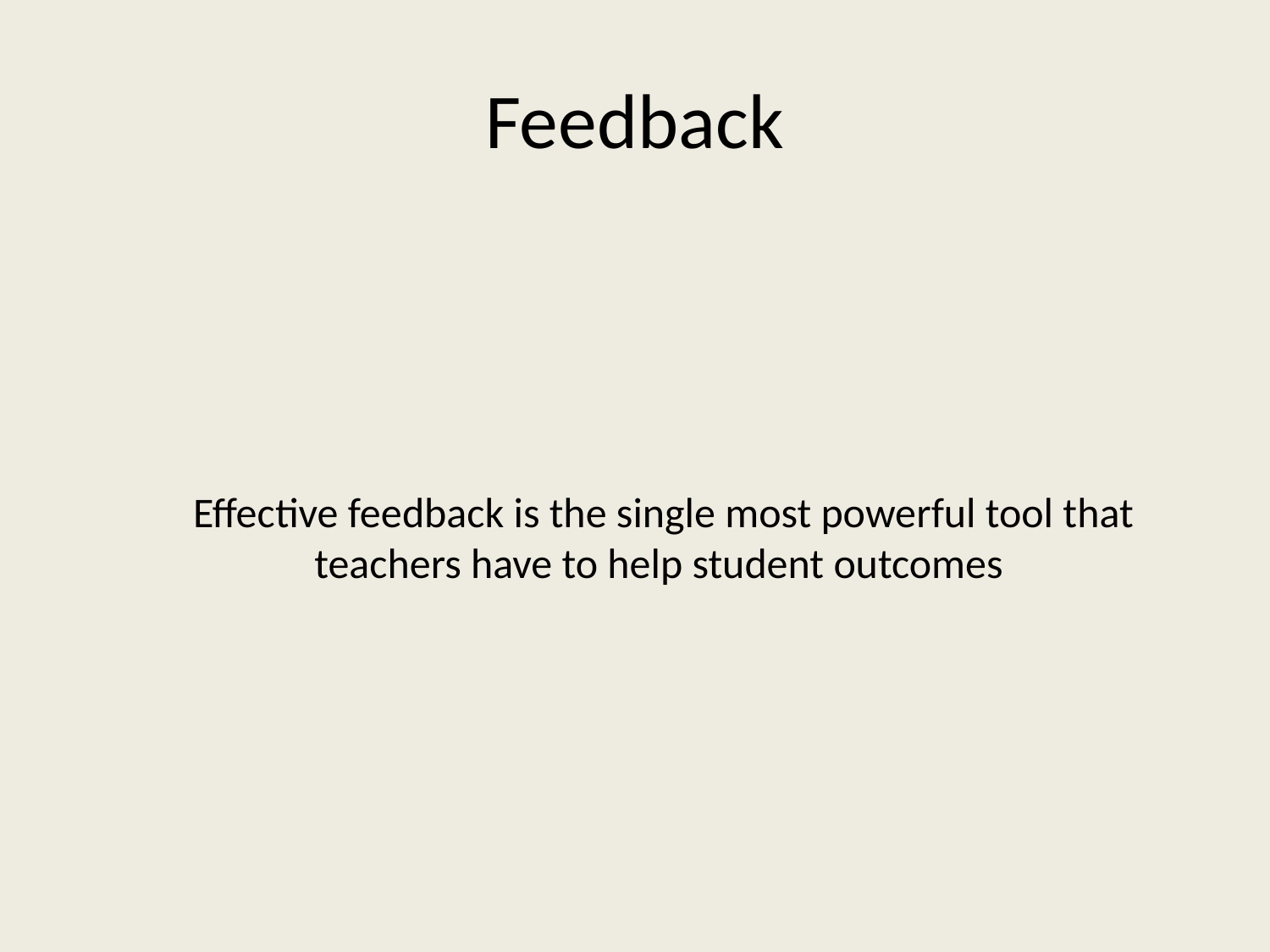

# Feedback
 Effective feedback is the single most powerful tool that teachers have to help student outcomes

## Slide 3
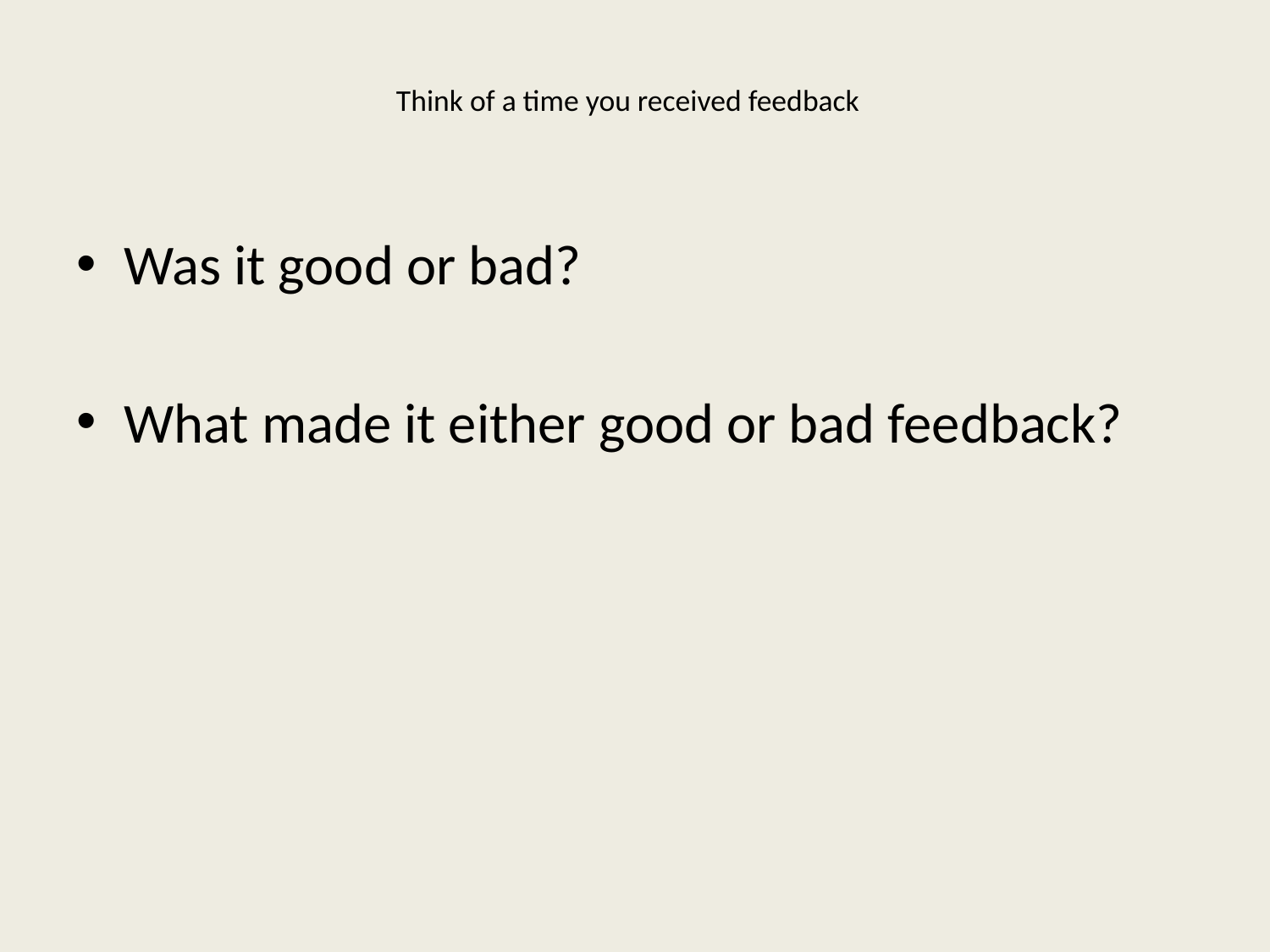

# Think of a time you received feedback
Was it good or bad?
What made it either good or bad feedback?

## Slide 4
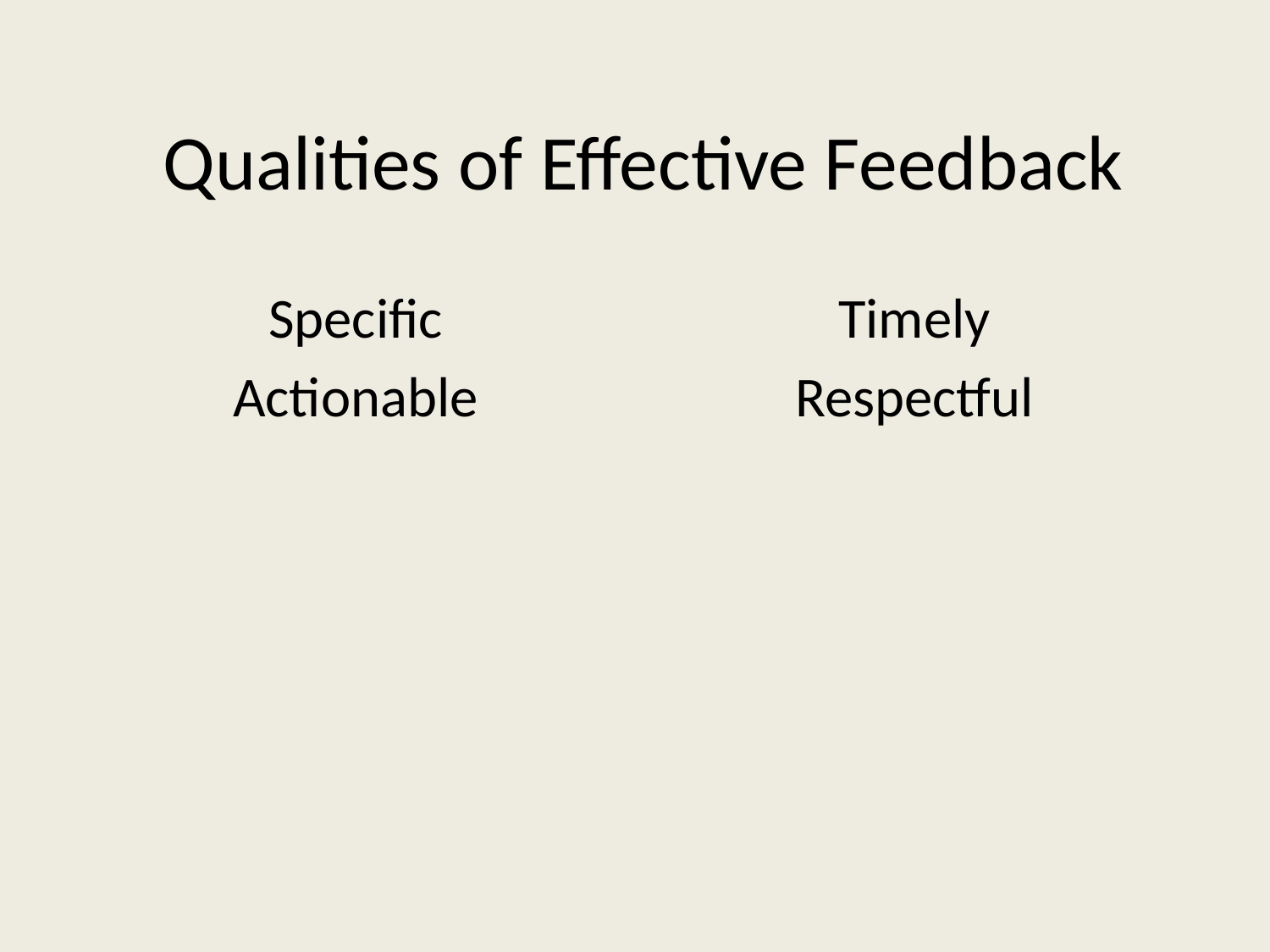

# Qualities of Effective Feedback
Specific
Actionable
Timely
Respectful

## Slide 5
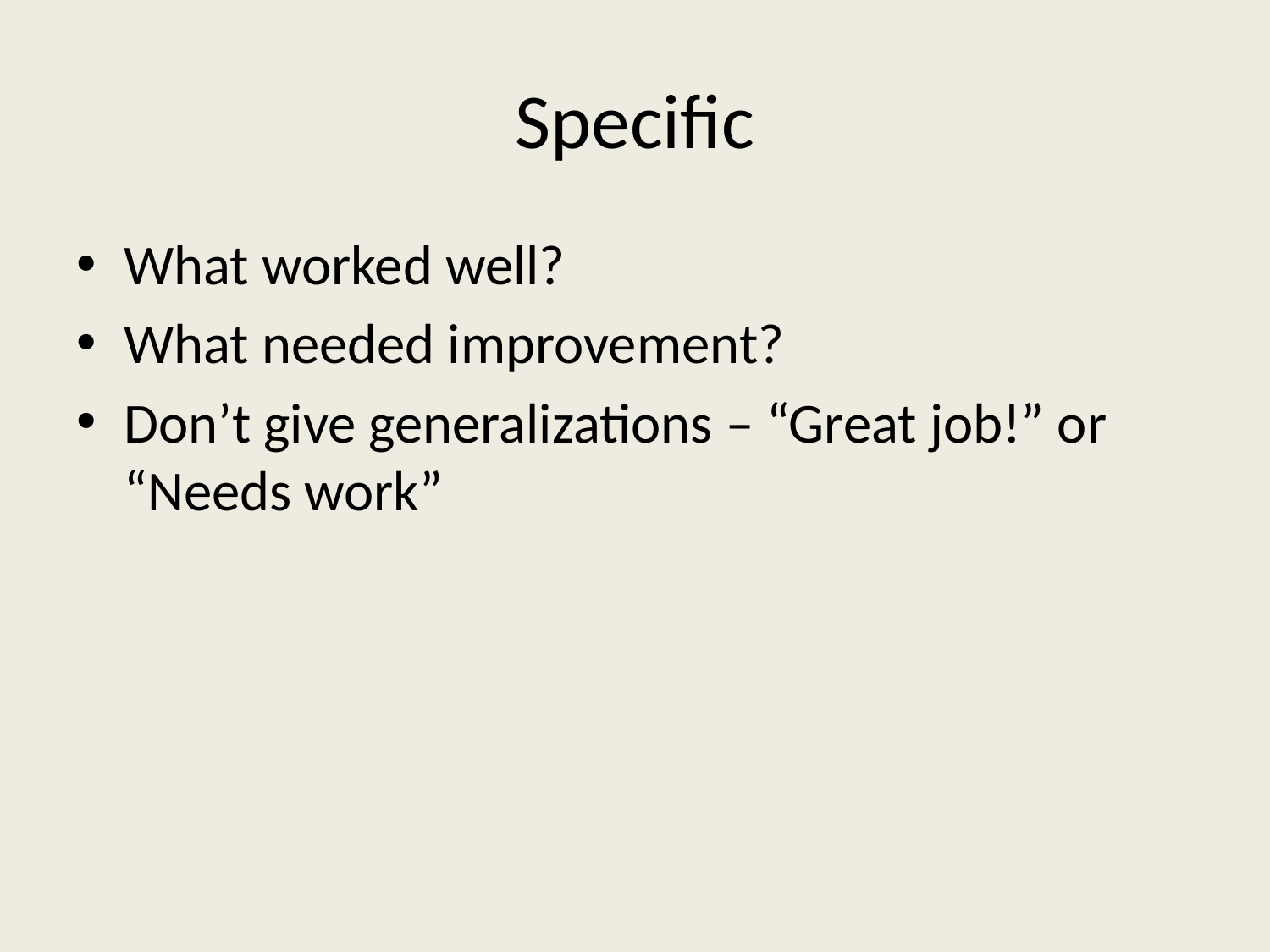

# Specific
What worked well?
What needed improvement?
Don’t give generalizations – “Great job!” or “Needs work”

## Slide 6
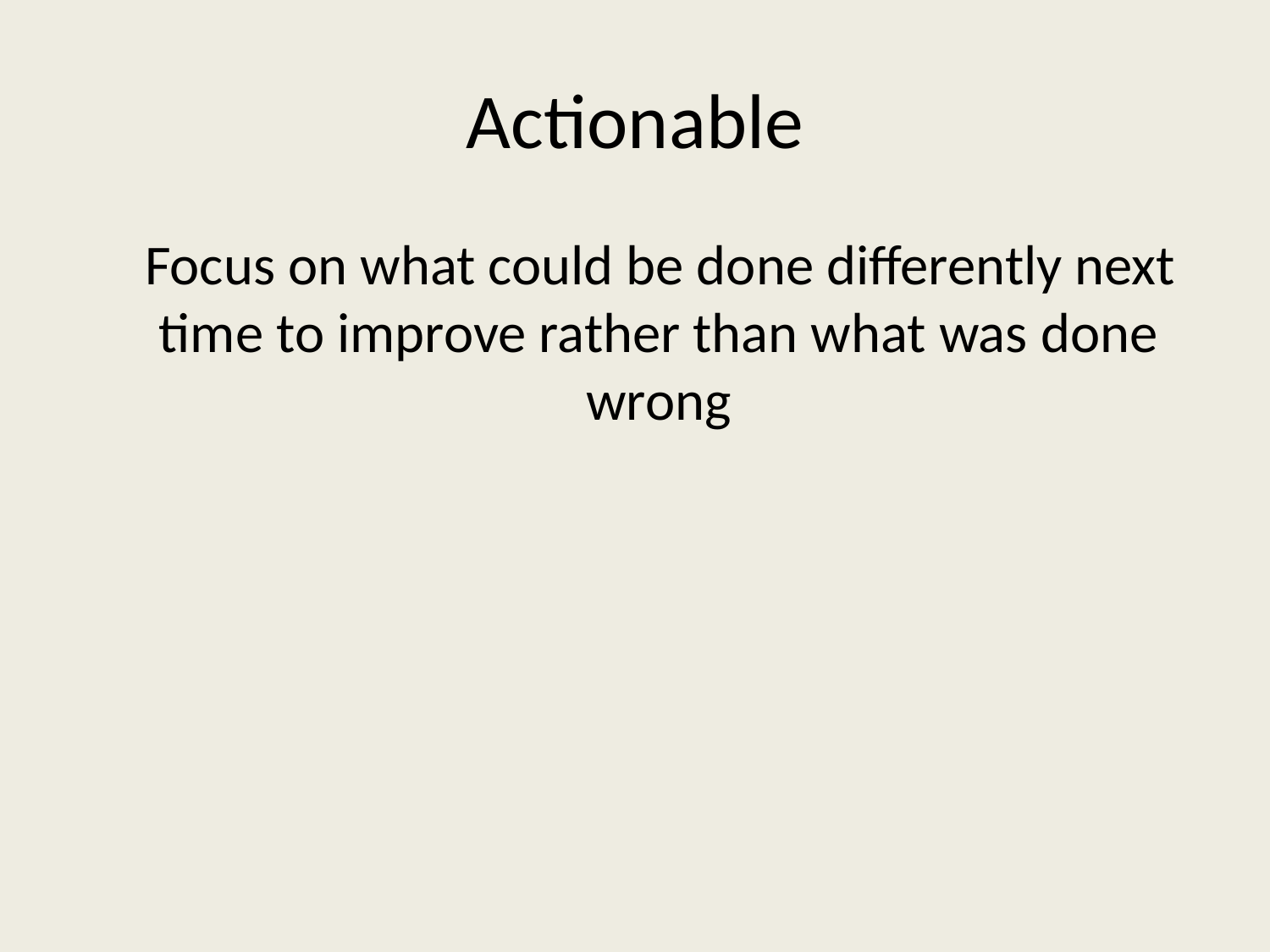

# Actionable
 Focus on what could be done differently next time to improve rather than what was done wrong

## Slide 7
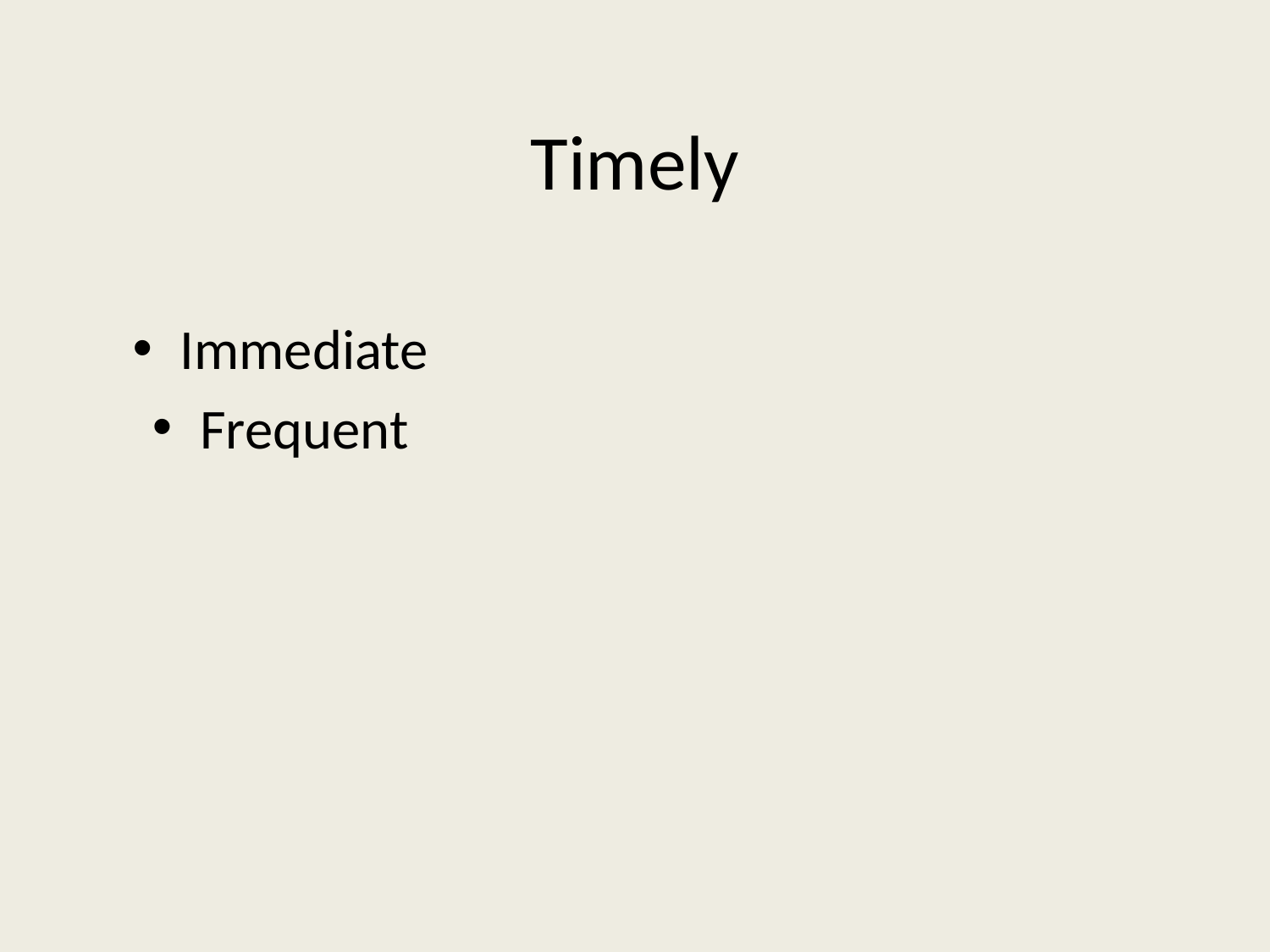

# Timely
Immediate
Frequent

## Slide 8
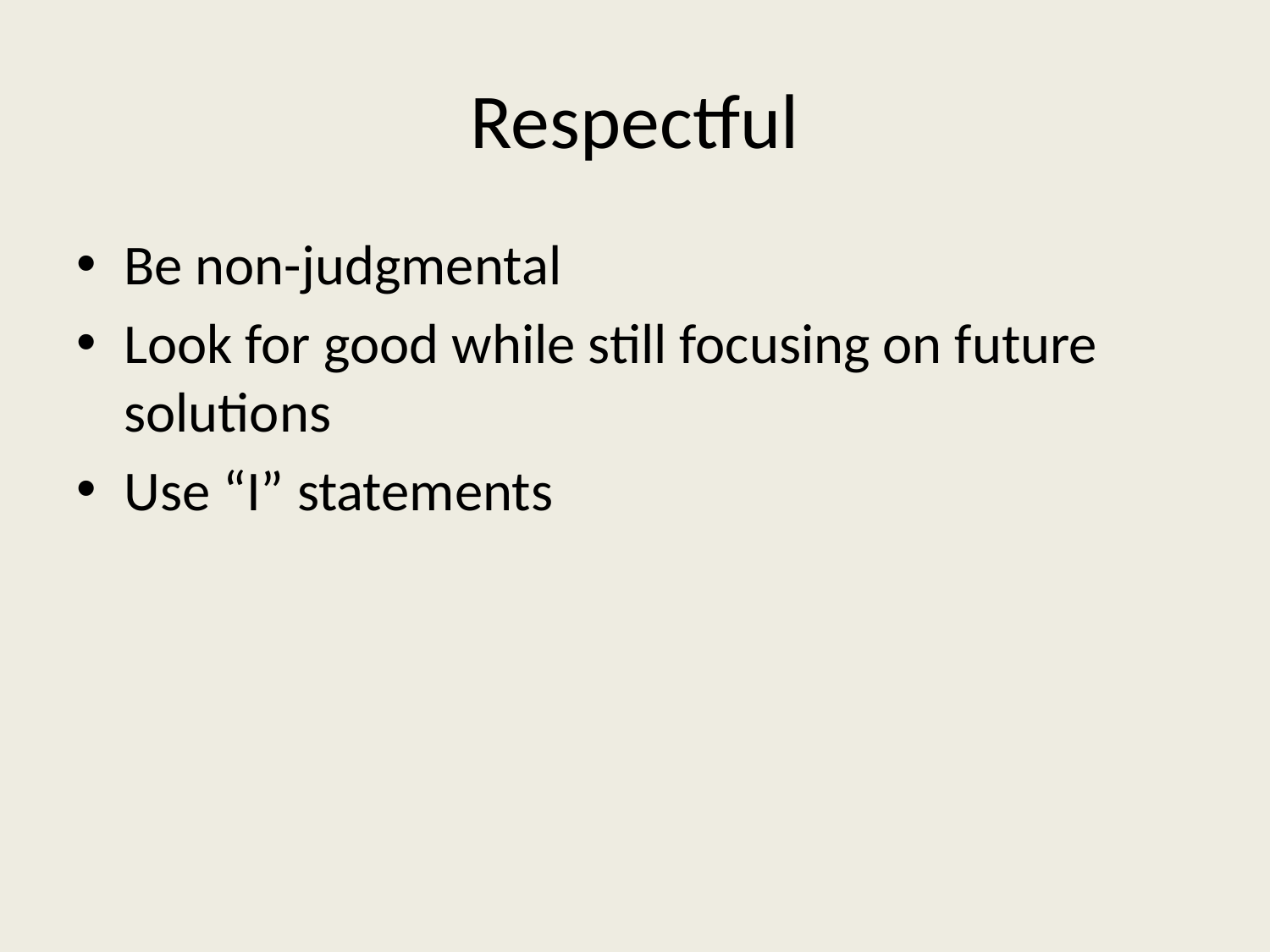

# Respectful
Be non-judgmental
Look for good while still focusing on future solutions
Use “I” statements

## Slide 9
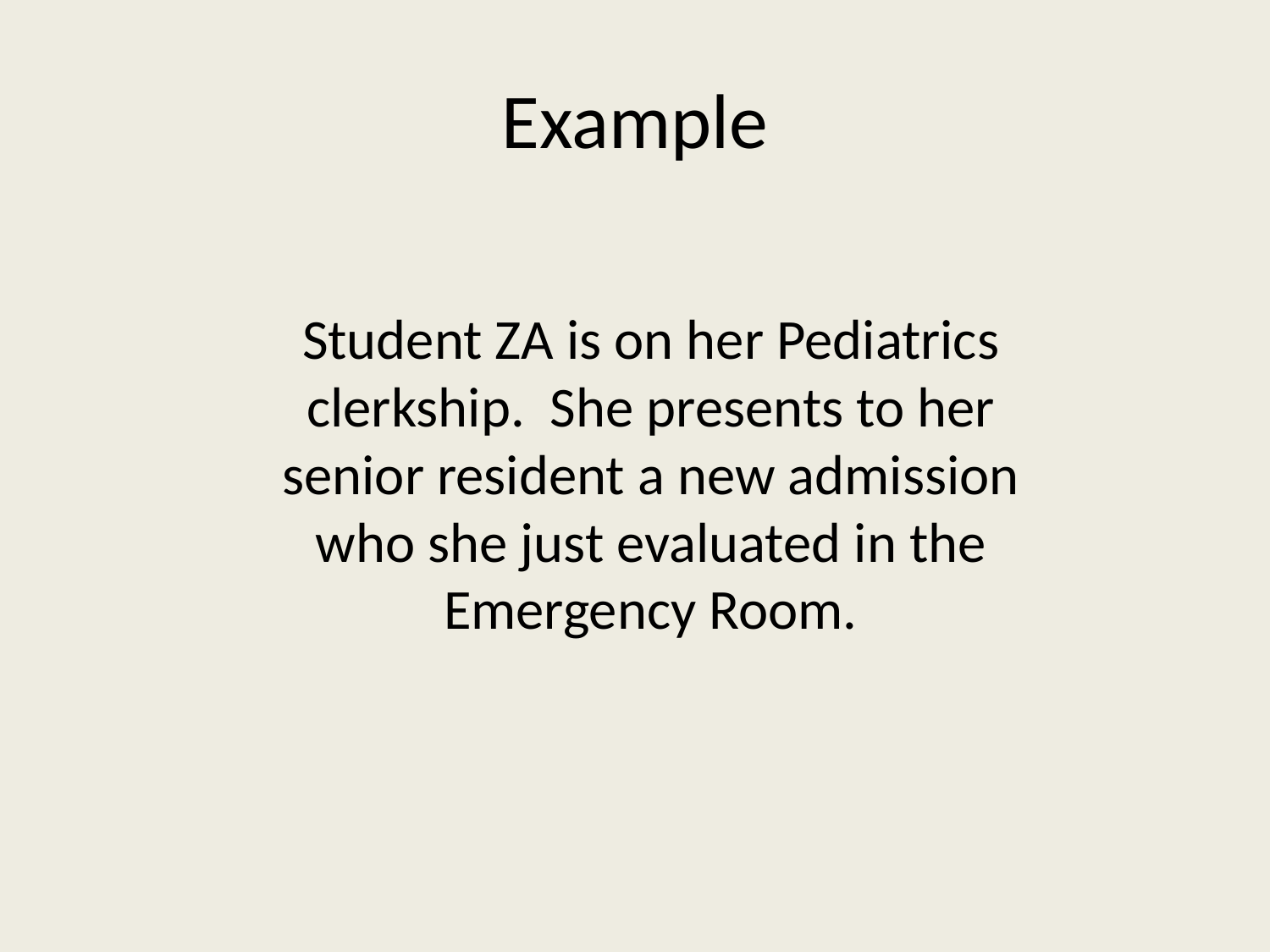

# Example
Student ZA is on her Pediatrics clerkship. She presents to her senior resident a new admission who she just evaluated in the Emergency Room.

## Slide 10
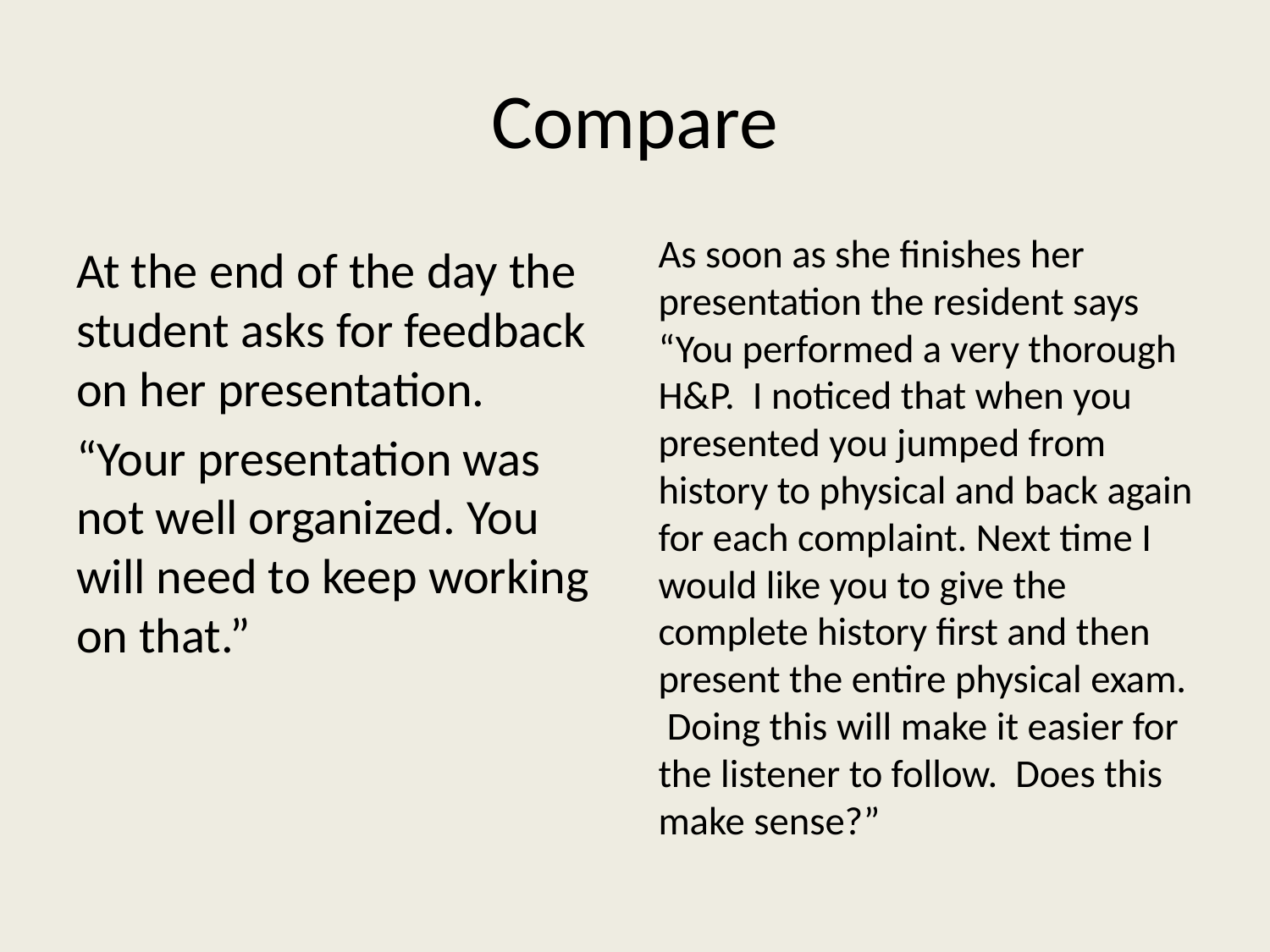

# Compare
As soon as she finishes her presentation the resident says “You performed a very thorough H&P. I noticed that when you presented you jumped from history to physical and back again for each complaint. Next time I would like you to give the complete history first and then present the entire physical exam. Doing this will make it easier for the listener to follow. Does this make sense?”
At the end of the day the student asks for feedback on her presentation.
“Your presentation was not well organized. You will need to keep working on that.”

## Slide 11
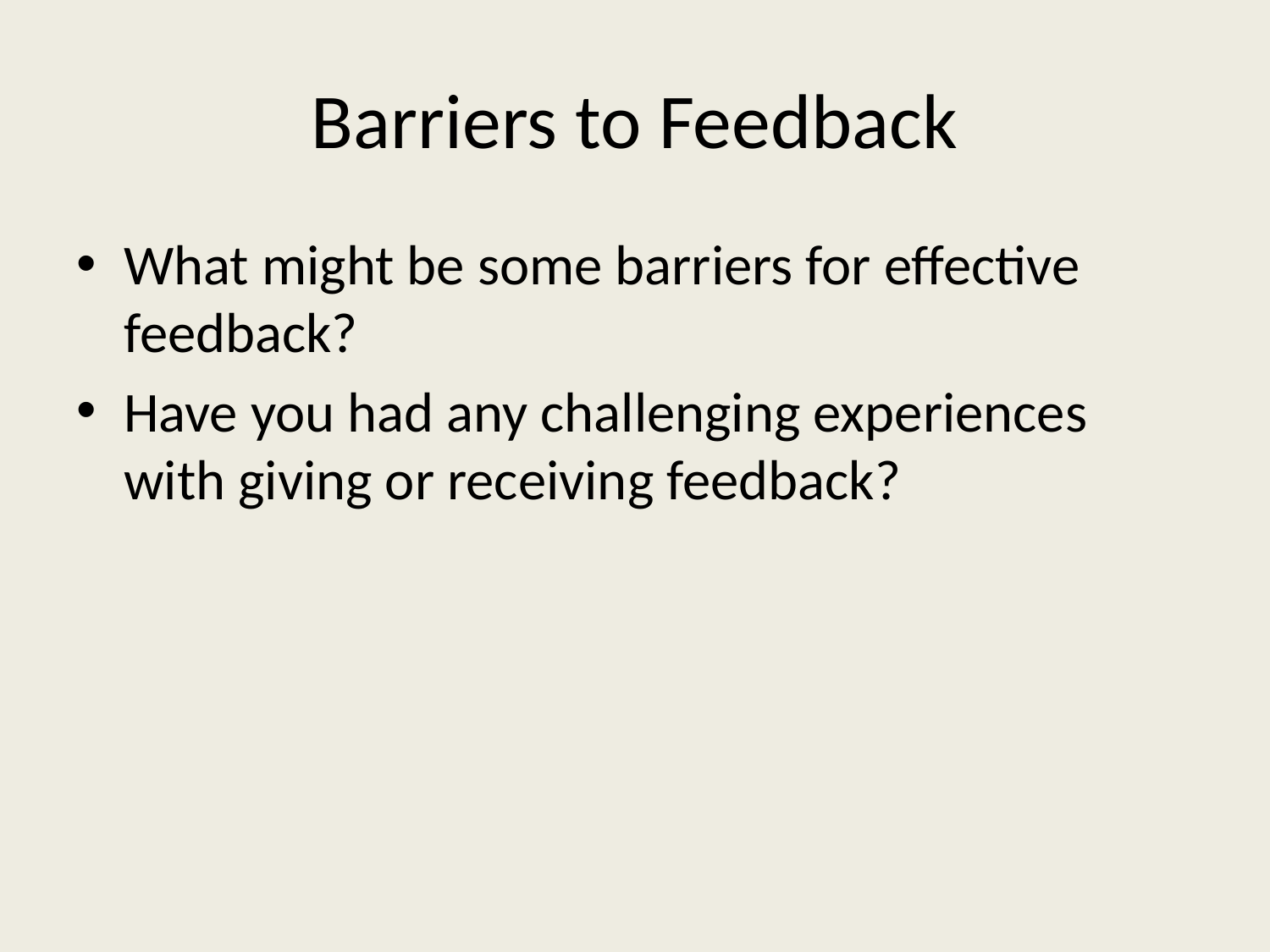

# Barriers to Feedback
What might be some barriers for effective feedback?
Have you had any challenging experiences with giving or receiving feedback?

## Slide 12
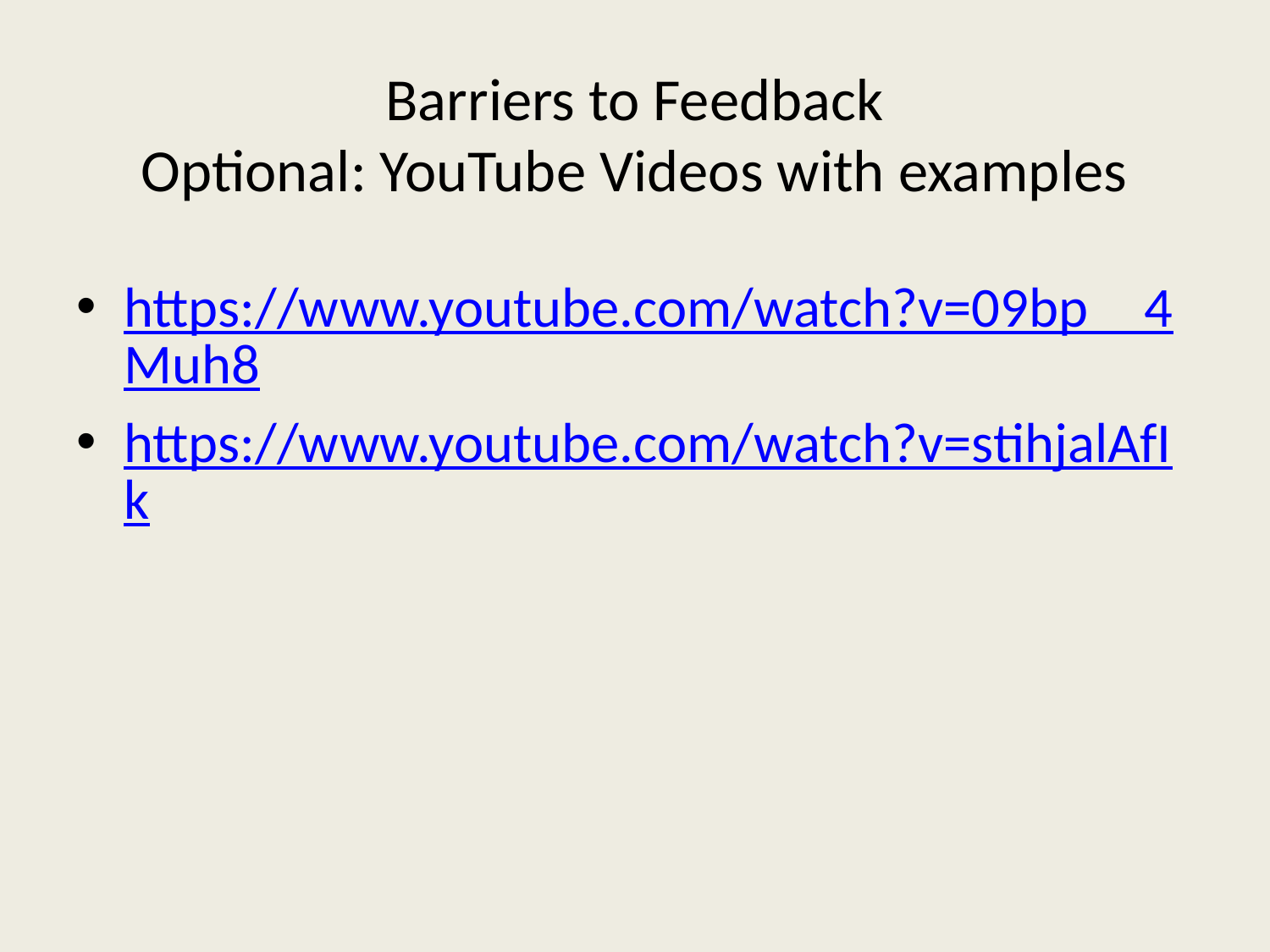

# Barriers to Feedback Optional: YouTube Videos with examples
https://www.youtube.com/watch?v=09bp__4Muh8
https://www.youtube.com/watch?v=stihjalAfIk

## Slide 13
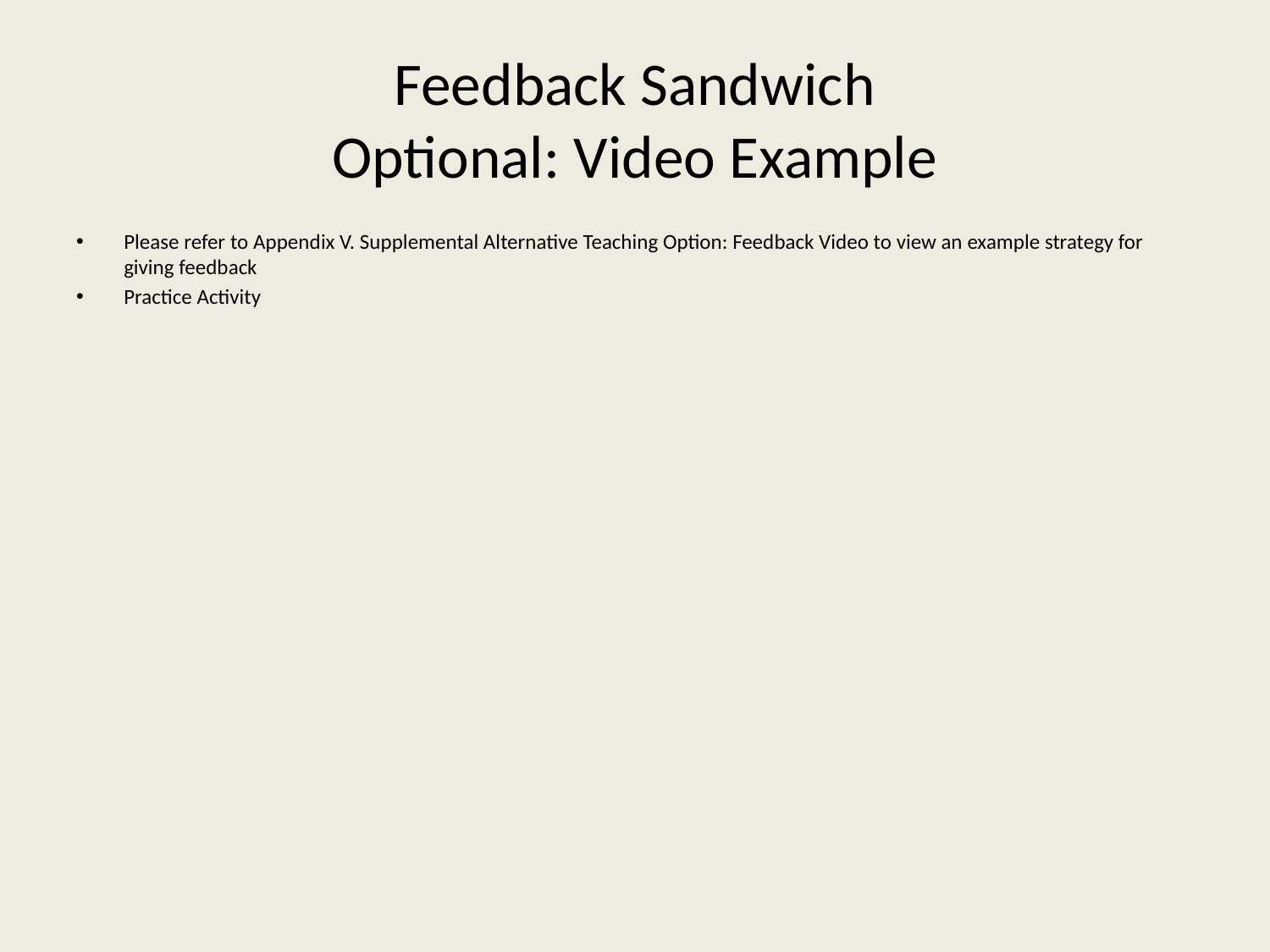

# Feedback Sandwich Optional: Video Example
Please refer to Appendix V. Supplemental Alternative Teaching Option: Feedback Video to view an example strategy for giving feedback
Practice Activity
