# Supplementary material for: Preparing Medical Students to Be Physician Leaders: A Leadership Training Program for Students Designed and Led by Students
Source: MedEdPORTAL. 2019 Dec 13;15:10863. doi: 10.15766/mep_2374-8265.10863 (PMC7012310; doi:10.15766/mep_2374-8265.10863)
Supplement: Supplementary file 1 — A. Session 1 PPT Leadership Styles.pptx B. Session 2 PPT Teamwork.pptx C. Session 3 PPT Delegation.pptx D. Session 4 PPT Feedback.pptx E. Session 5 PPT Direction.pptx F. Session 6 Optional Review PPT Consolidation.pptx G. Session 1 Activity Instructions.docx H. Session 2 Activity Instructions.docx I. Session 3 Activity Instructions.docx J. Session 4 Activity Instructions and Figure.docx K. Session 5 Activity Instructions.docx L. Session 6 Activity Instructions.docx M. Precourse and Postcourse Evaluation.docx N. Session 1 Evaluation.docx O. Session 2 Evaluation.docx P. Session 3 Evaluation.docx Q. Session 4 Evaluation.docx R. Session 5 Evaluation.docx S. Posttraining Evaluation.docx T. Supplemental Alternative Activity - PACE Palette.docx U. Supplemental Alternative Activity - ACLS Video.docx V. Supplemental Alternative Activity - Feedback Video.docx [file mep-15-10863-s001.zip › E. Session 5 PPT Direction.pptx]

## Slide 1
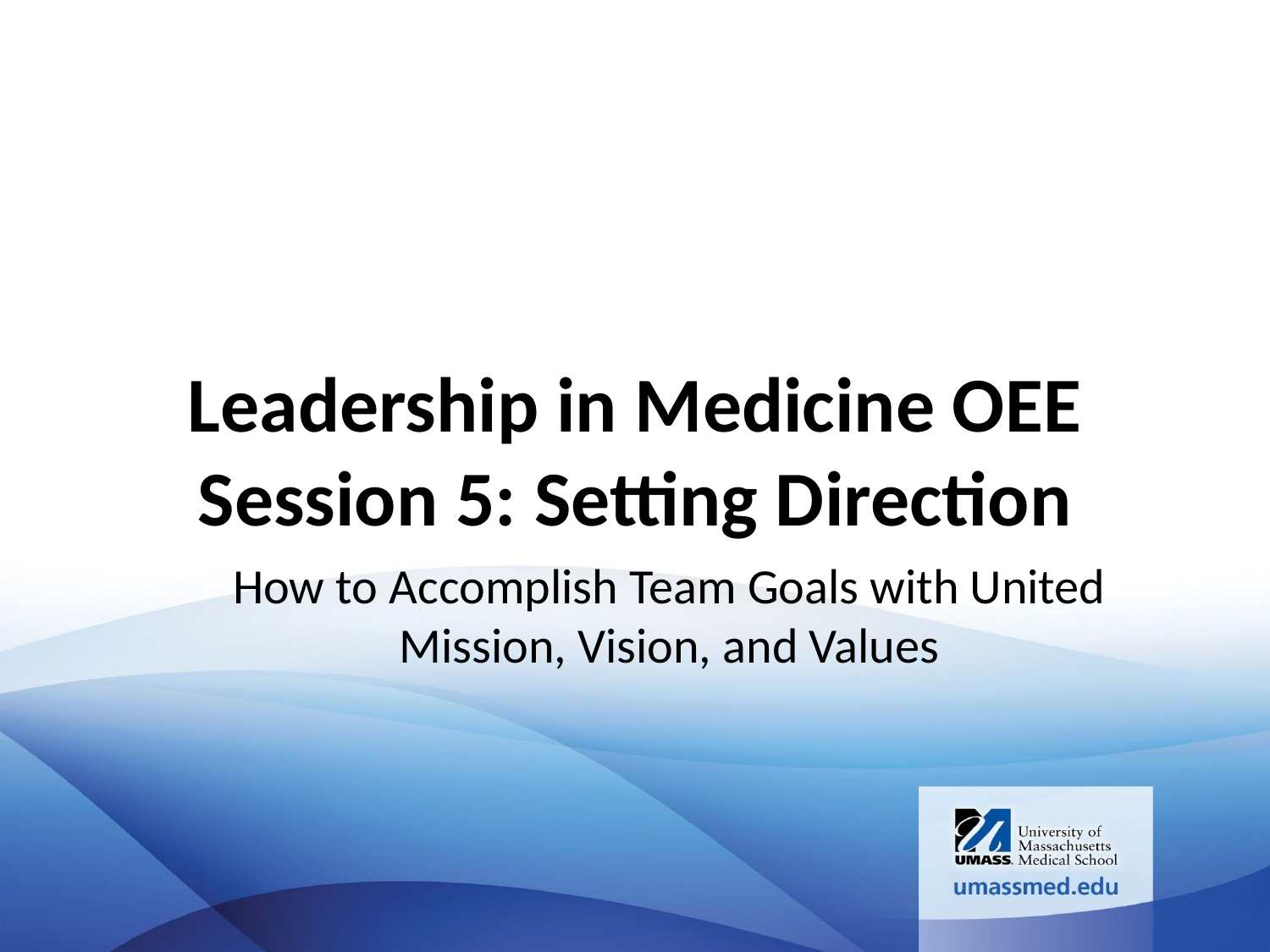

# Leadership in Medicine OEE Session 5: Setting Direction
How to Accomplish Team Goals with United Mission, Vision, and Values

## Slide 2
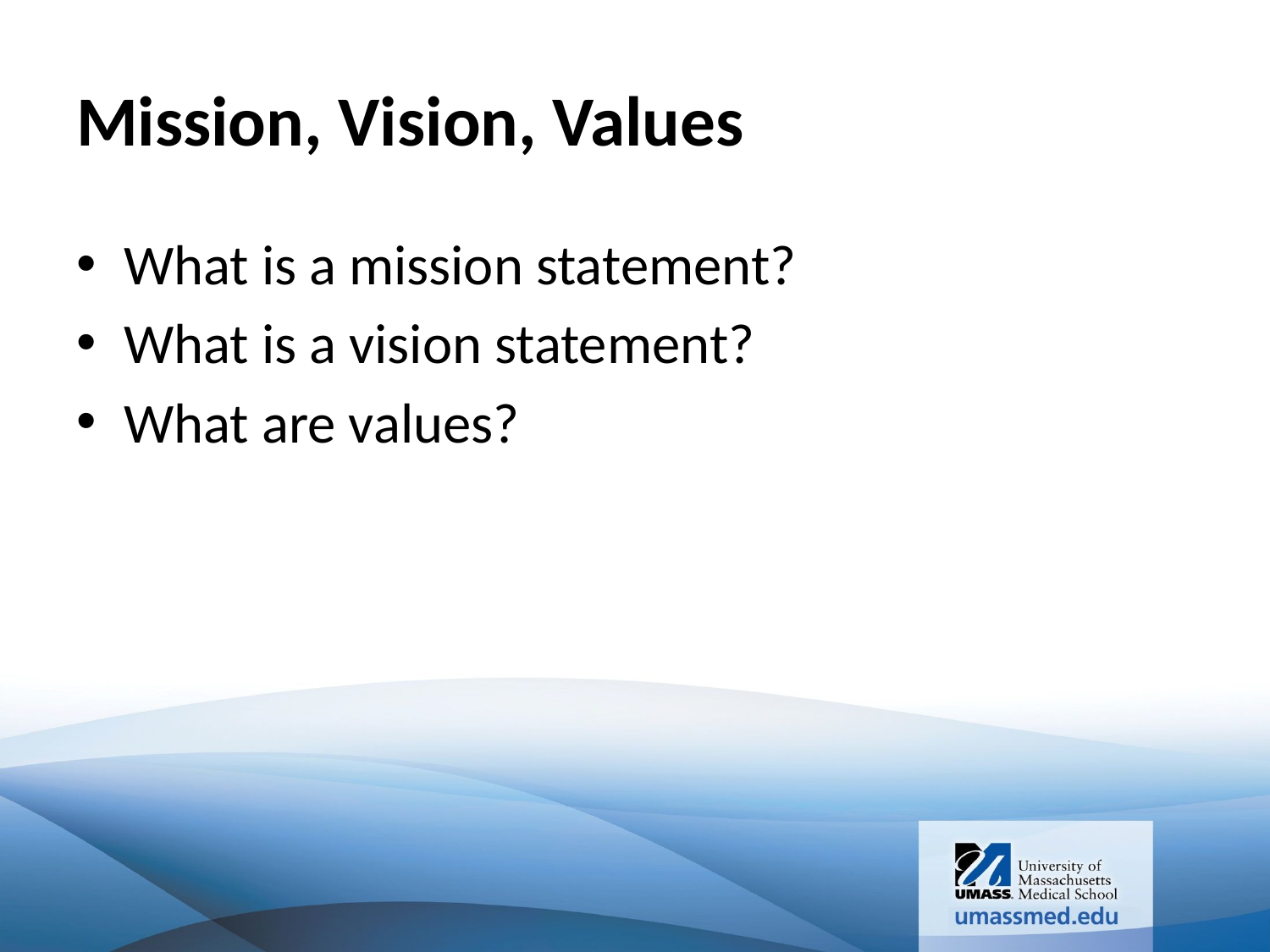

# Mission, Vision, Values
What is a mission statement?
What is a vision statement?
What are values?

## Slide 3
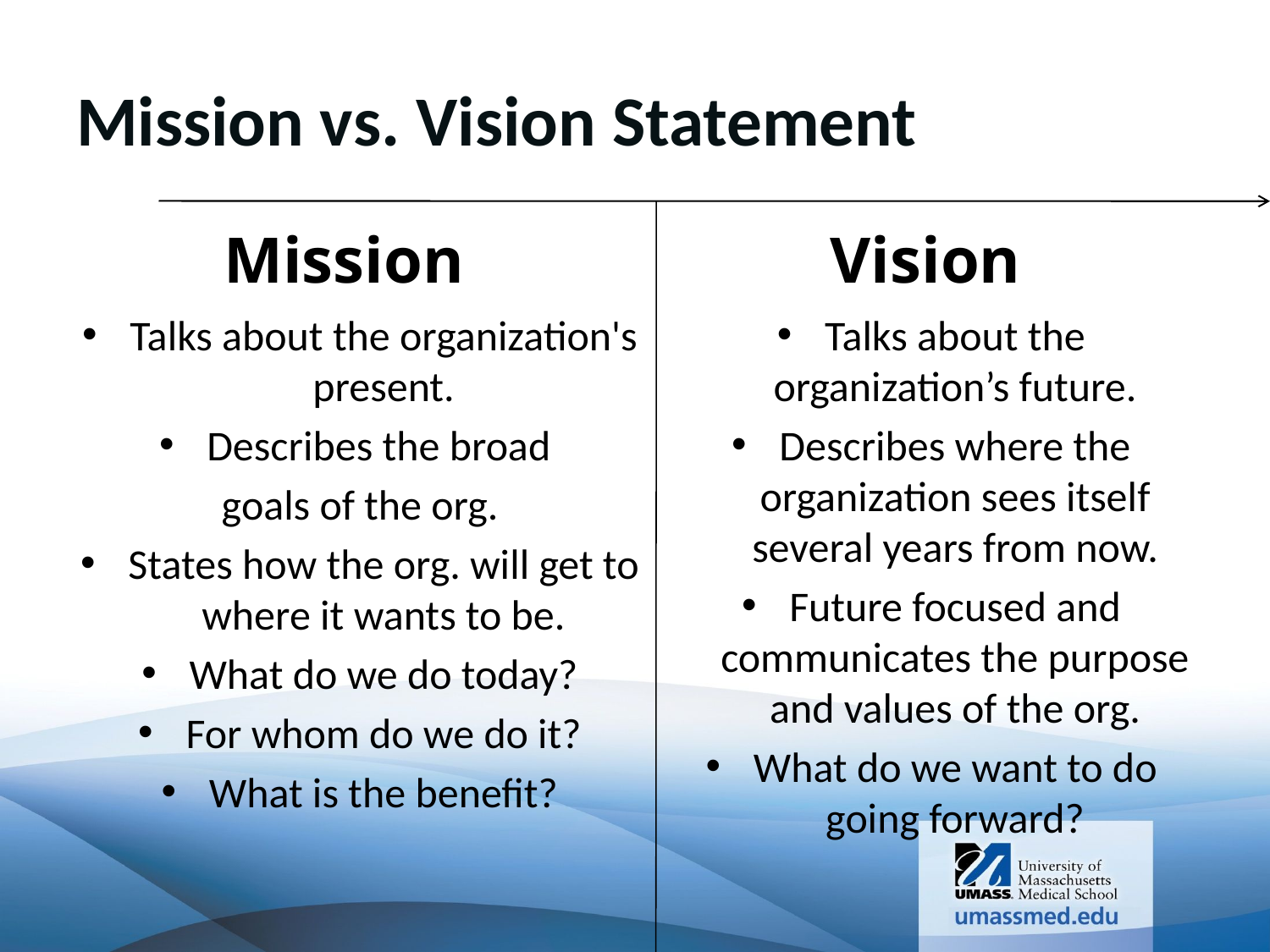

# Mission vs. Vision Statement
Mission
Vision
Talks about the organization's present.
Describes the broad
goals of the org.
States how the org. will get to where it wants to be.
What do we do today?
For whom do we do it?
What is the benefit?
Talks about the organization’s future.
Describes where the organization sees itself several years from now.
Future focused and communicates the purpose and values of the org.
What do we want to do going forward?

## Slide 4
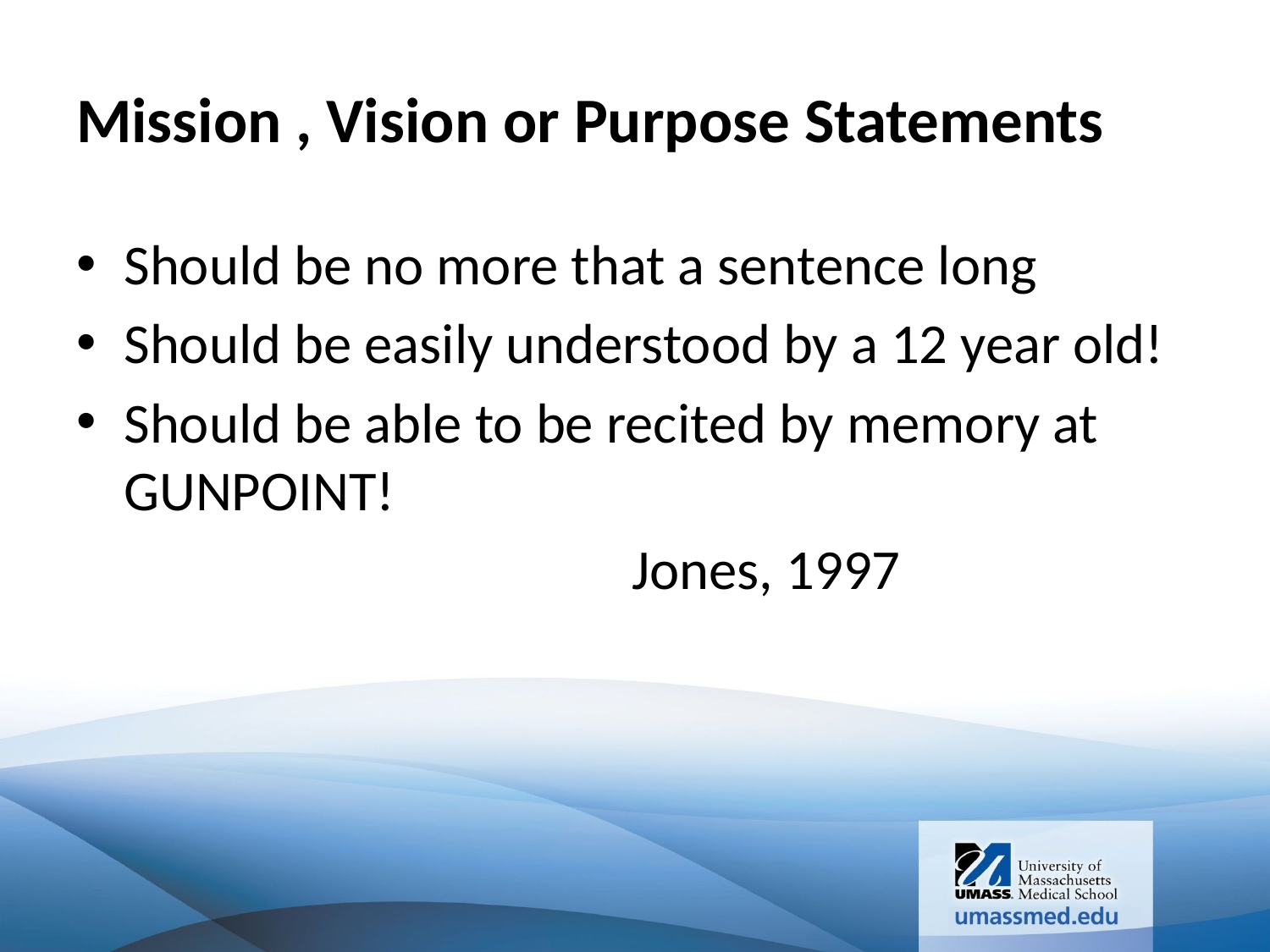

# Mission , Vision or Purpose Statements
Should be no more that a sentence long
Should be easily understood by a 12 year old!
Should be able to be recited by memory at GUNPOINT!
					Jones, 1997

## Slide 5
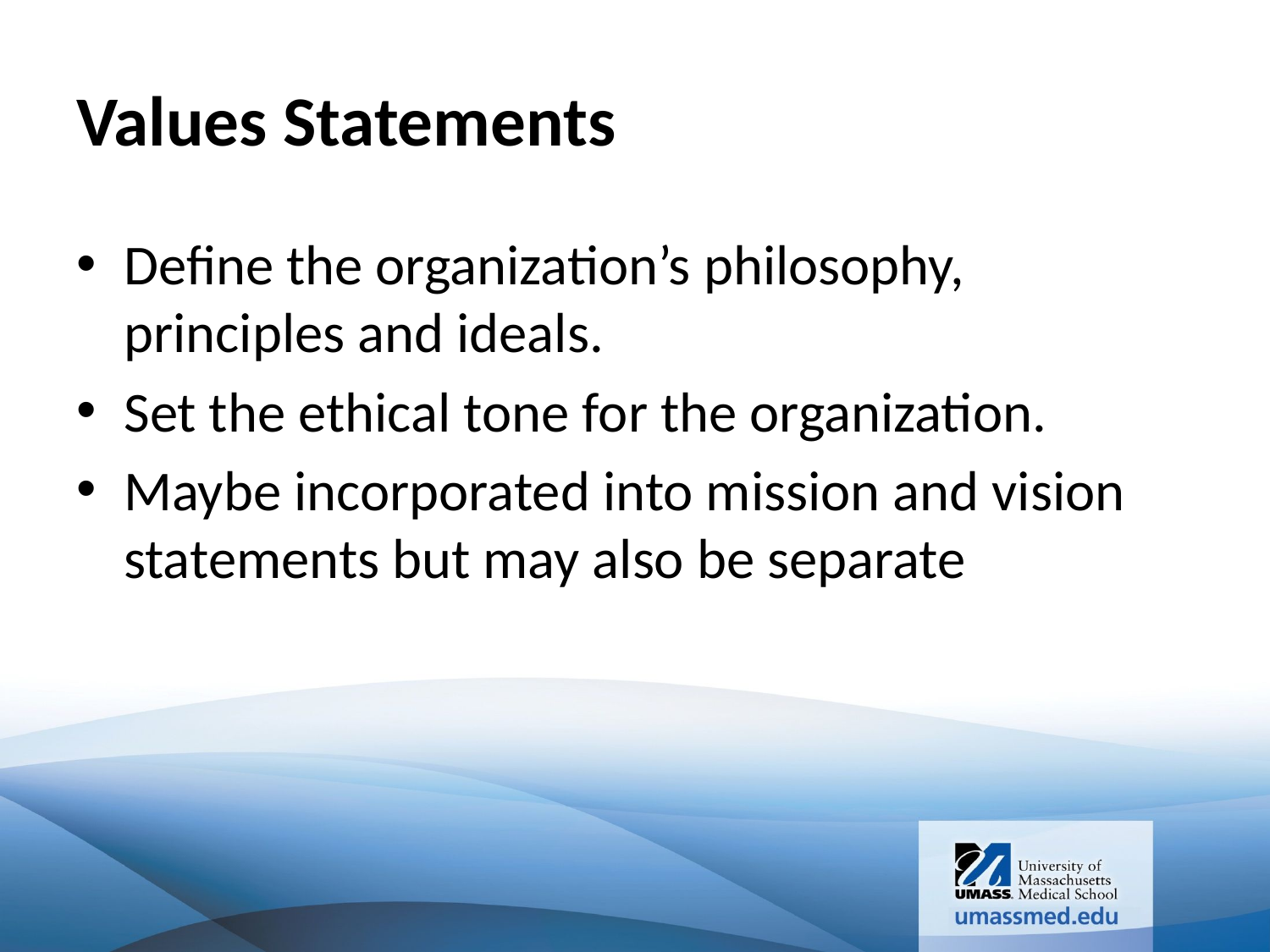

# Values Statements
Define the organization’s philosophy, principles and ideals.
Set the ethical tone for the organization.
Maybe incorporated into mission and vision statements but may also be separate

## Slide 6
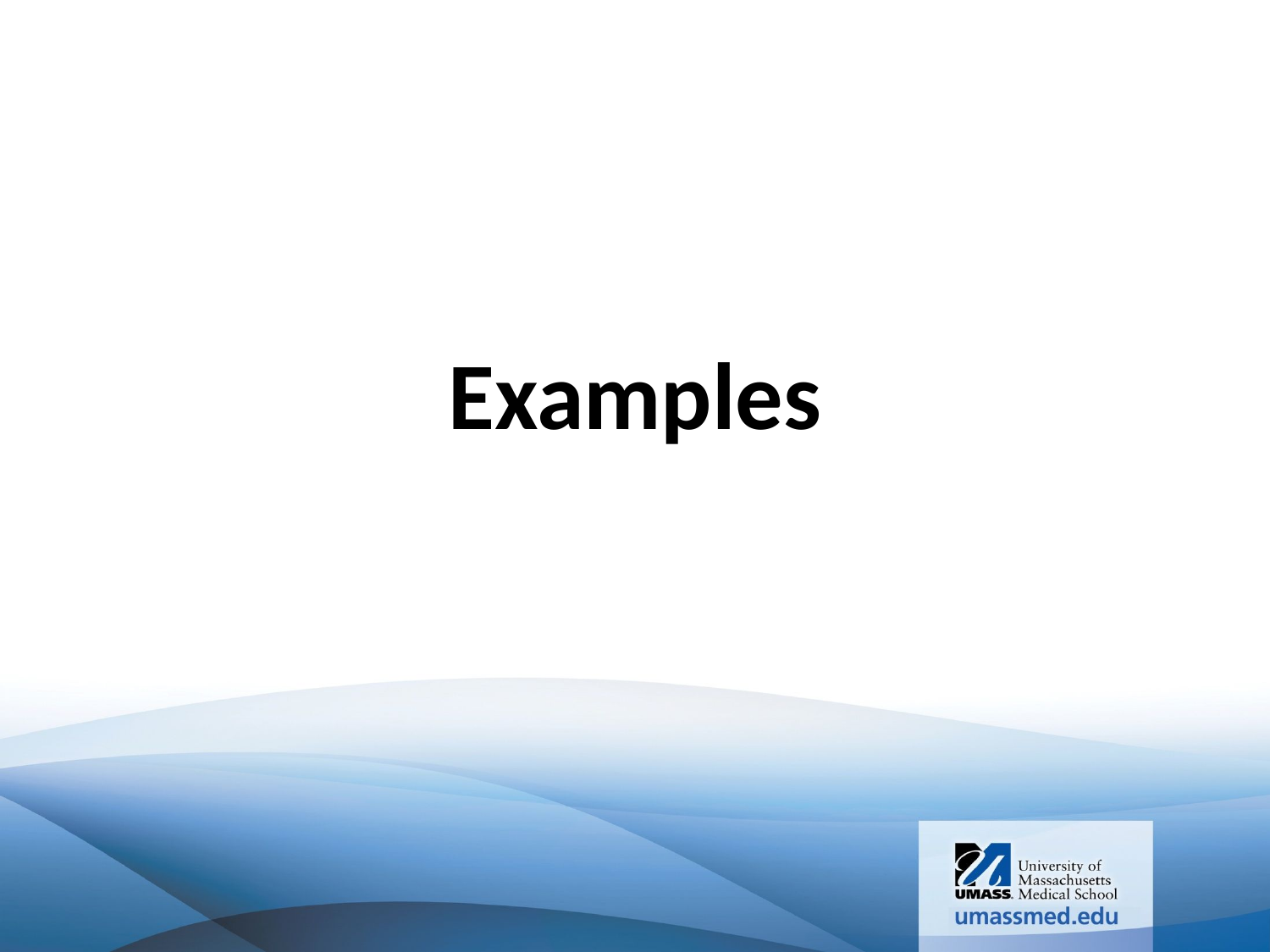

Examples

## Slide 7
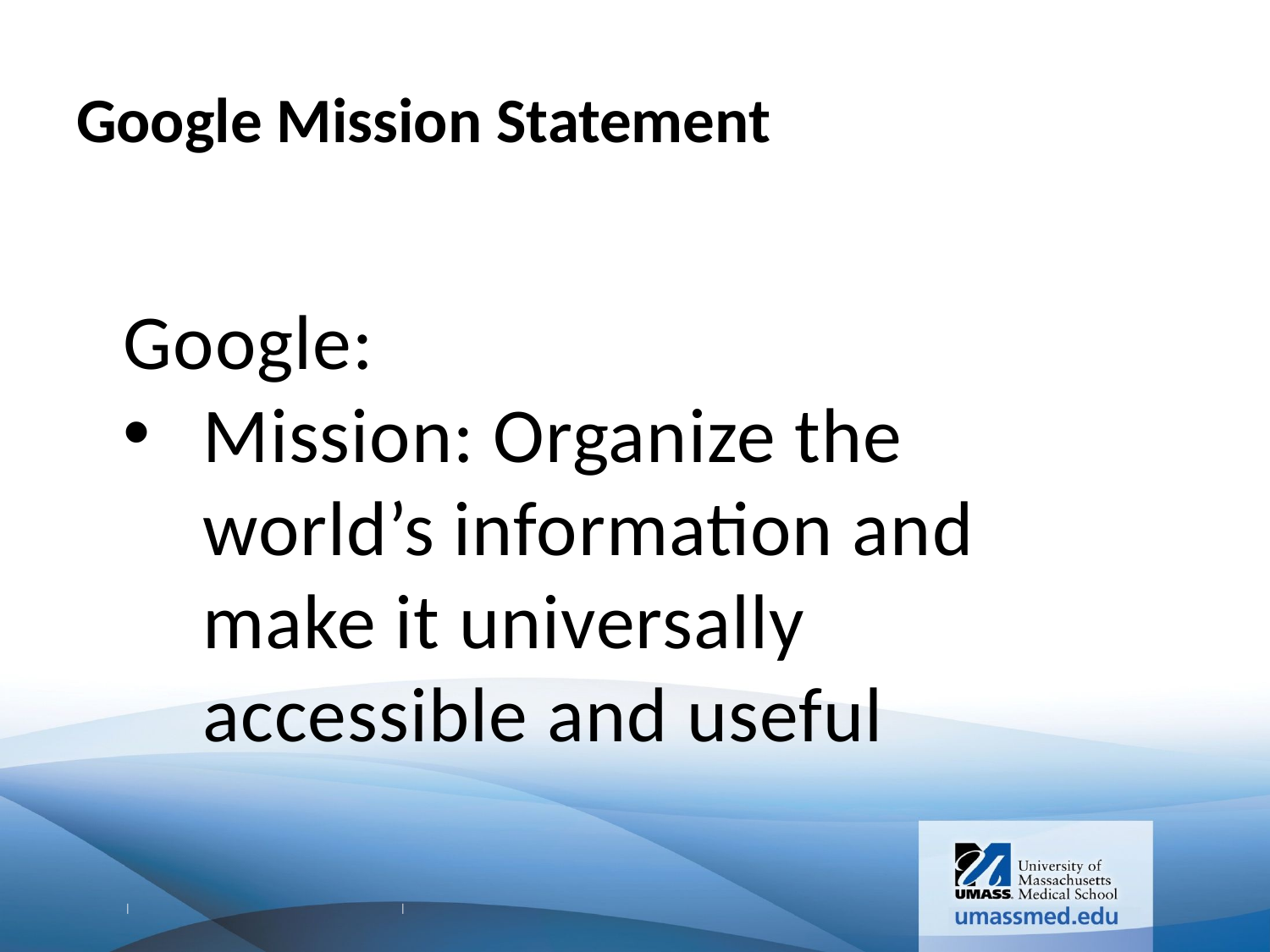

# Google Mission Statement
Google:
Mission: Organize the world’s information and make it universally accessible and useful

## Slide 8
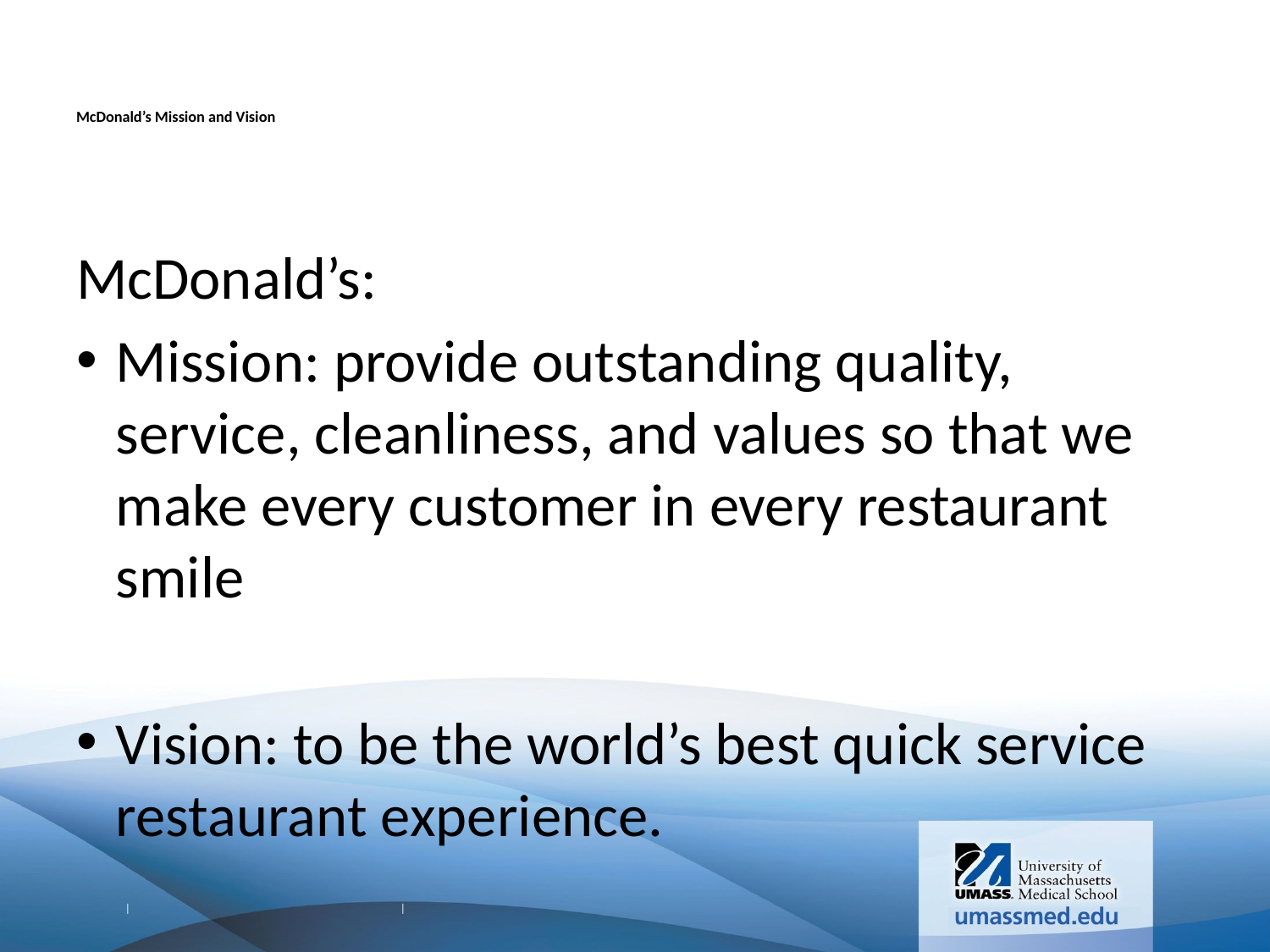

# McDonald’s Mission and Vision
McDonald’s:
Mission: provide outstanding quality, service, cleanliness, and values so that we make every customer in every restaurant smile
Vision: to be the world’s best quick service restaurant experience.

## Slide 9
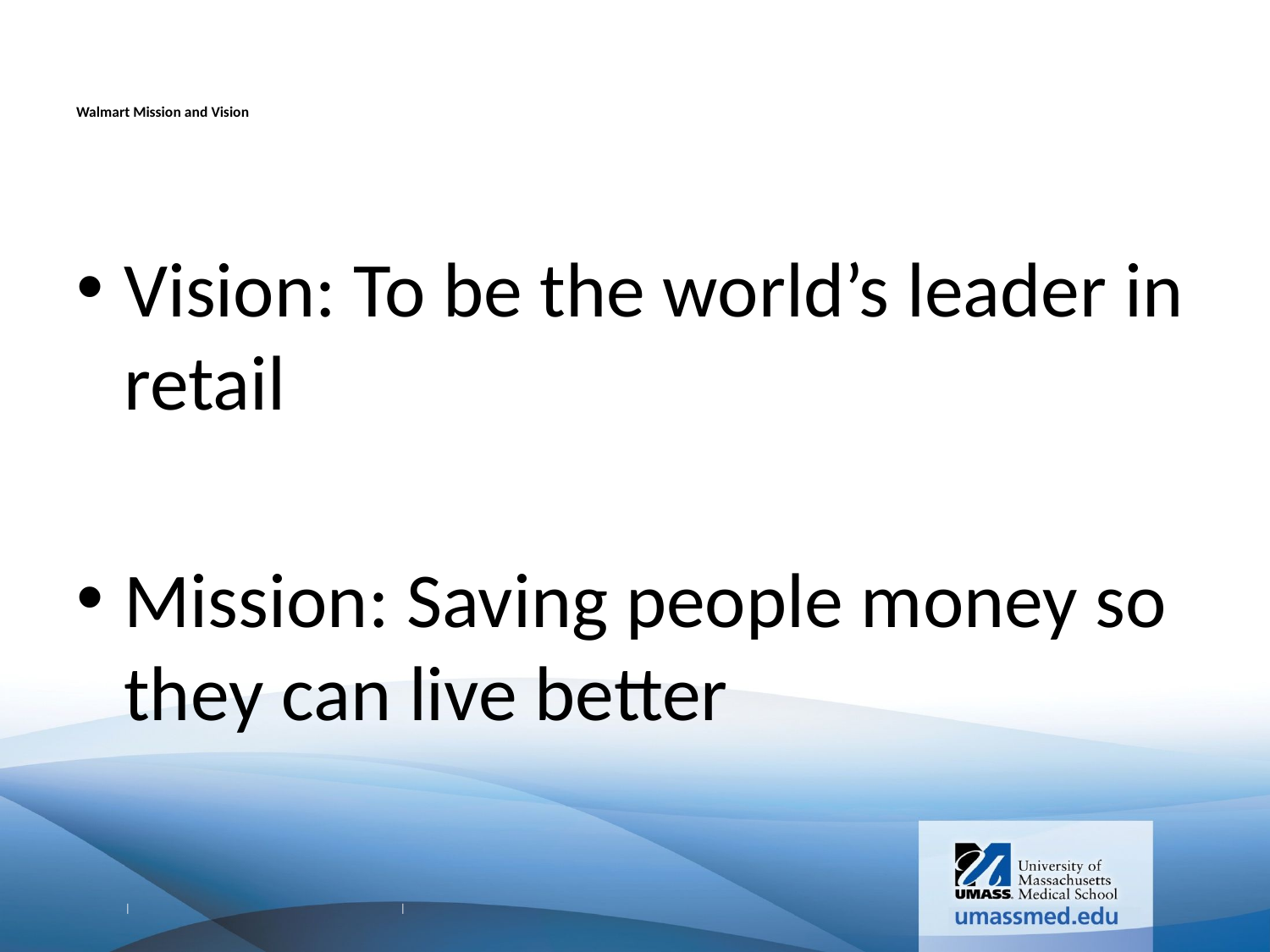

# Walmart Mission and Vision
Vision: To be the world’s leader in retail
Mission: Saving people money so they can live better

## Slide 10
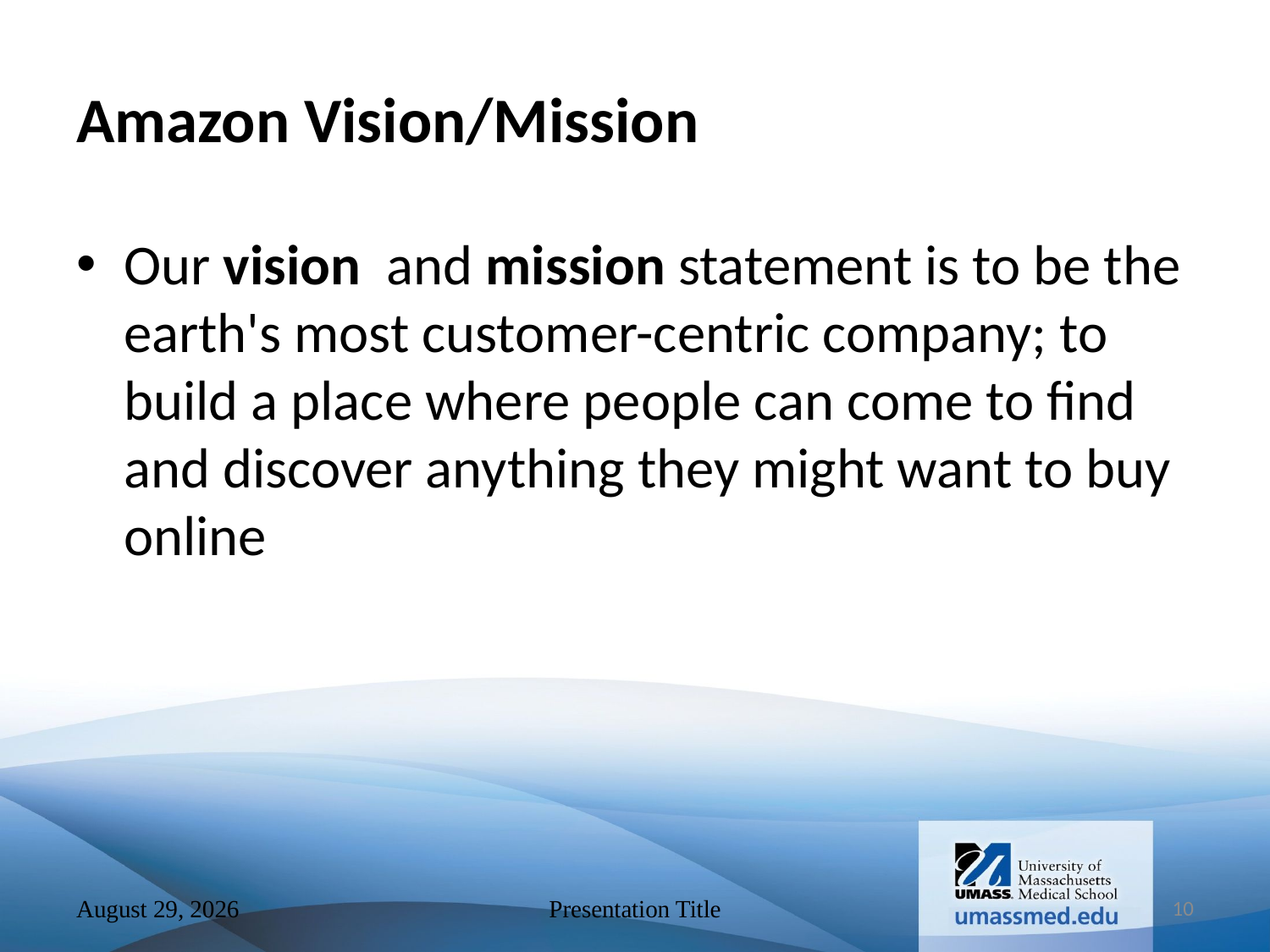

# Amazon Vision/Mission
Our vision and mission statement is to be the earth's most customer-centric company; to build a place where people can come to find and discover anything they might want to buy online
July 30, 2019
Presentation Title
10

## Slide 11
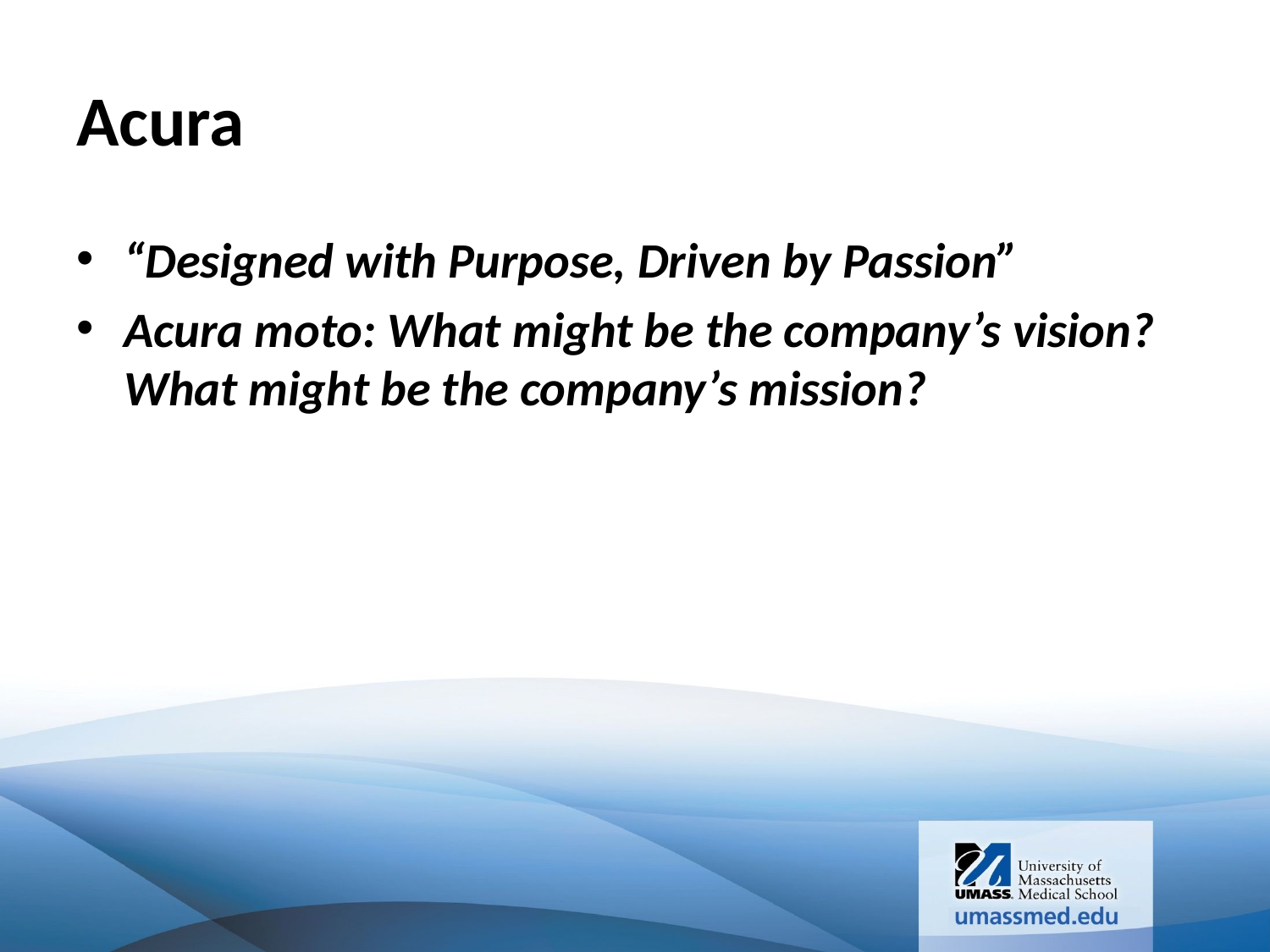

# Acura
“Designed with Purpose, Driven by Passion”
Acura moto: What might be the company’s vision? What might be the company’s mission?

## Slide 12
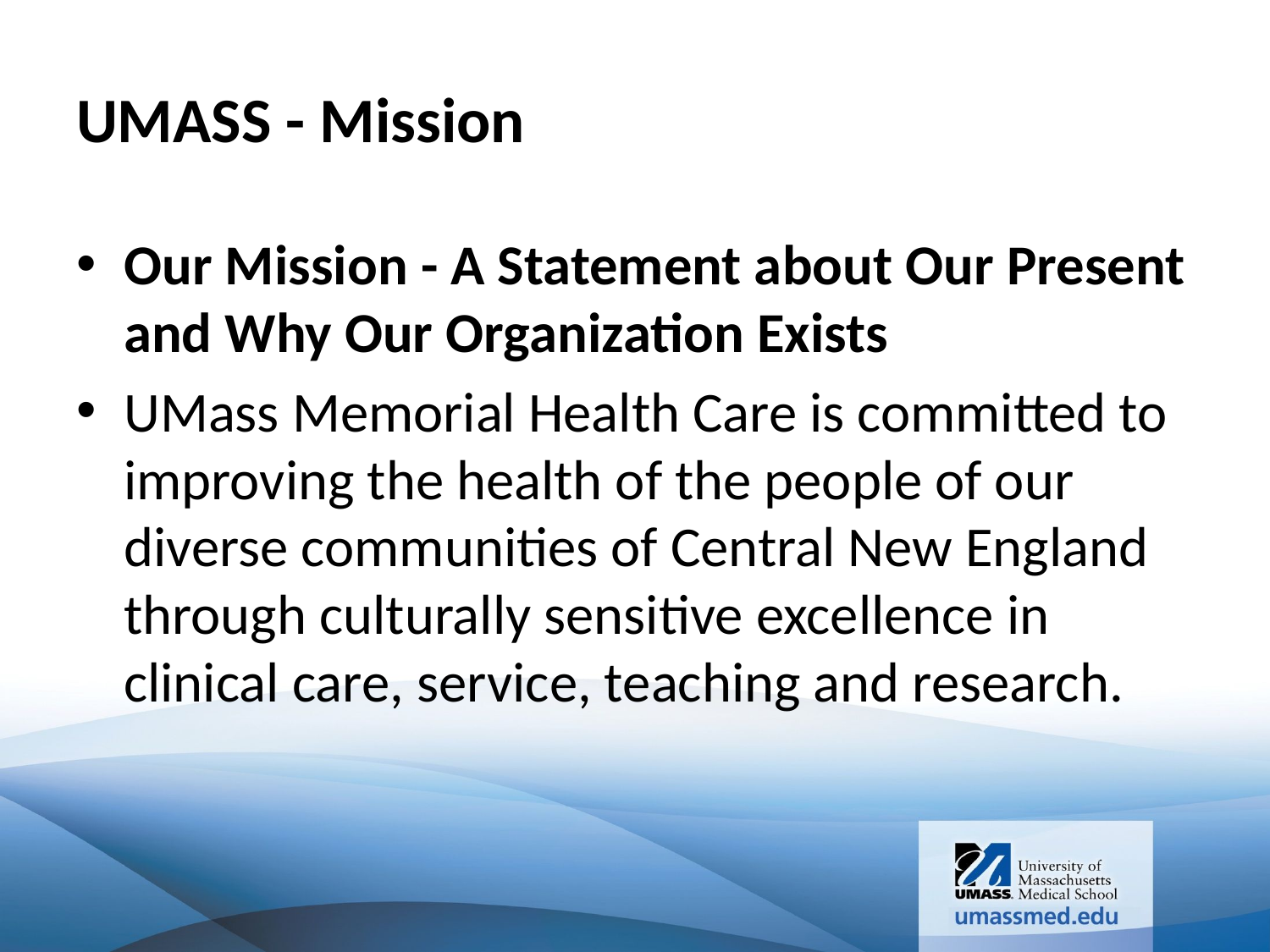

# UMASS - Mission
Our Mission - A Statement about Our Present and Why Our Organization Exists
UMass Memorial Health Care is committed to improving the health of the people of our diverse communities of Central New England through culturally sensitive excellence in clinical care, service, teaching and research.

## Slide 13
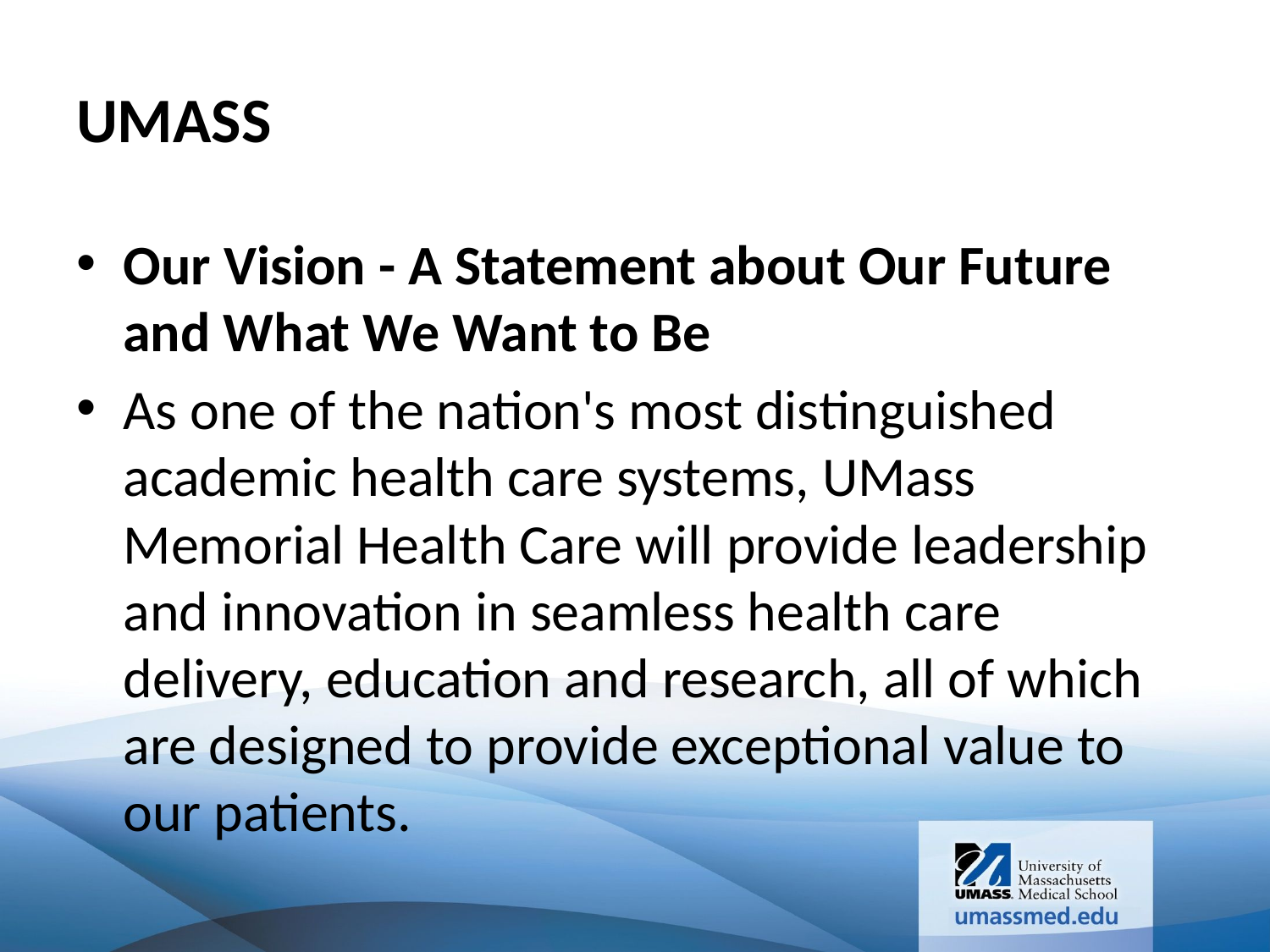

# UMASS
Our Vision - A Statement about Our Future and What We Want to Be
As one of the nation's most distinguished academic health care systems, UMass Memorial Health Care will provide leadership and innovation in seamless health care delivery, education and research, all of which are designed to provide exceptional value to our patients.

## Slide 14
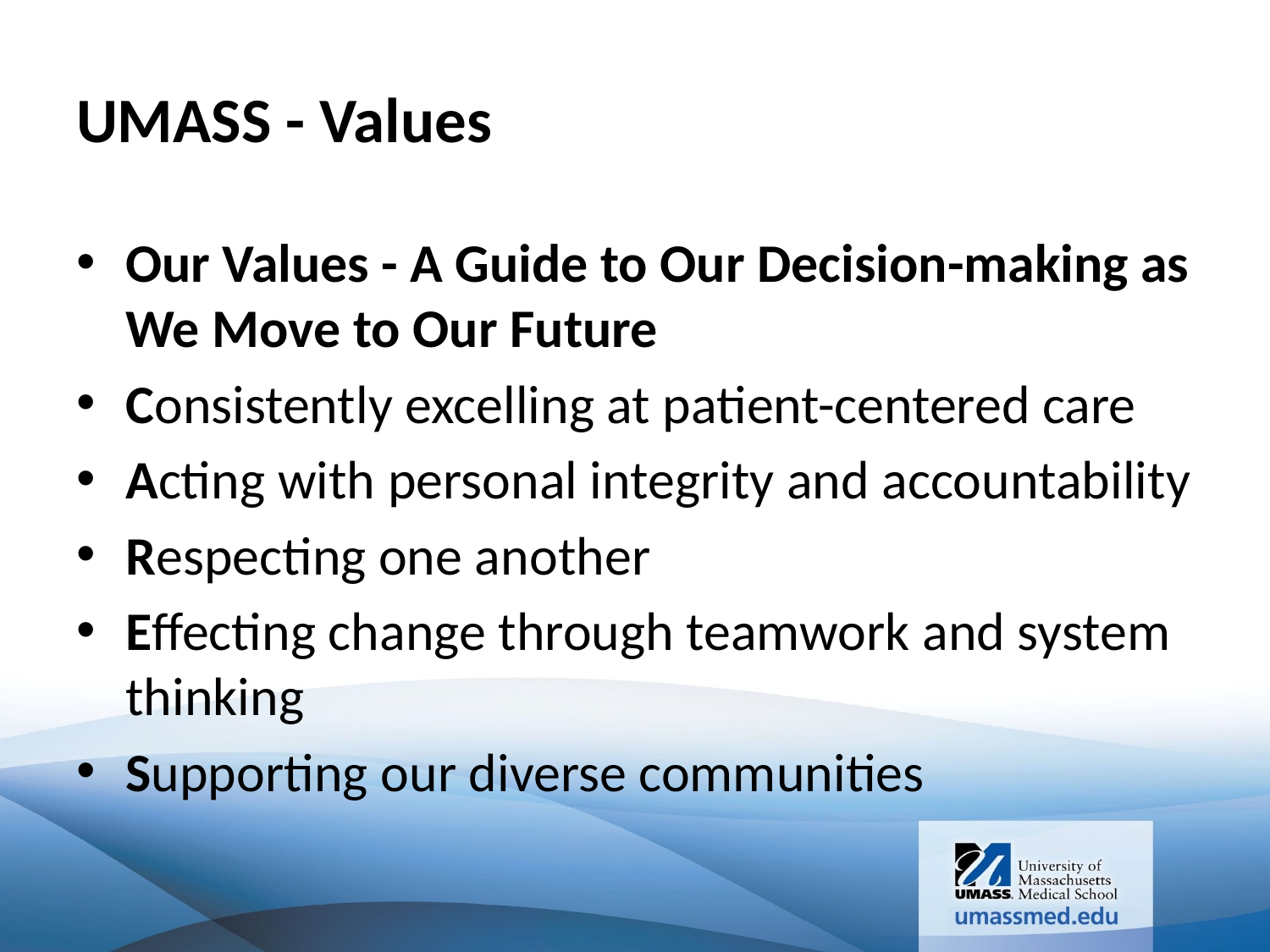

# UMASS - Values
Our Values - A Guide to Our Decision-making as We Move to Our Future
Consistently excelling at patient-centered care
Acting with personal integrity and accountability
Respecting one another
Effecting change through teamwork and system thinking
Supporting our diverse communities

## Slide 15
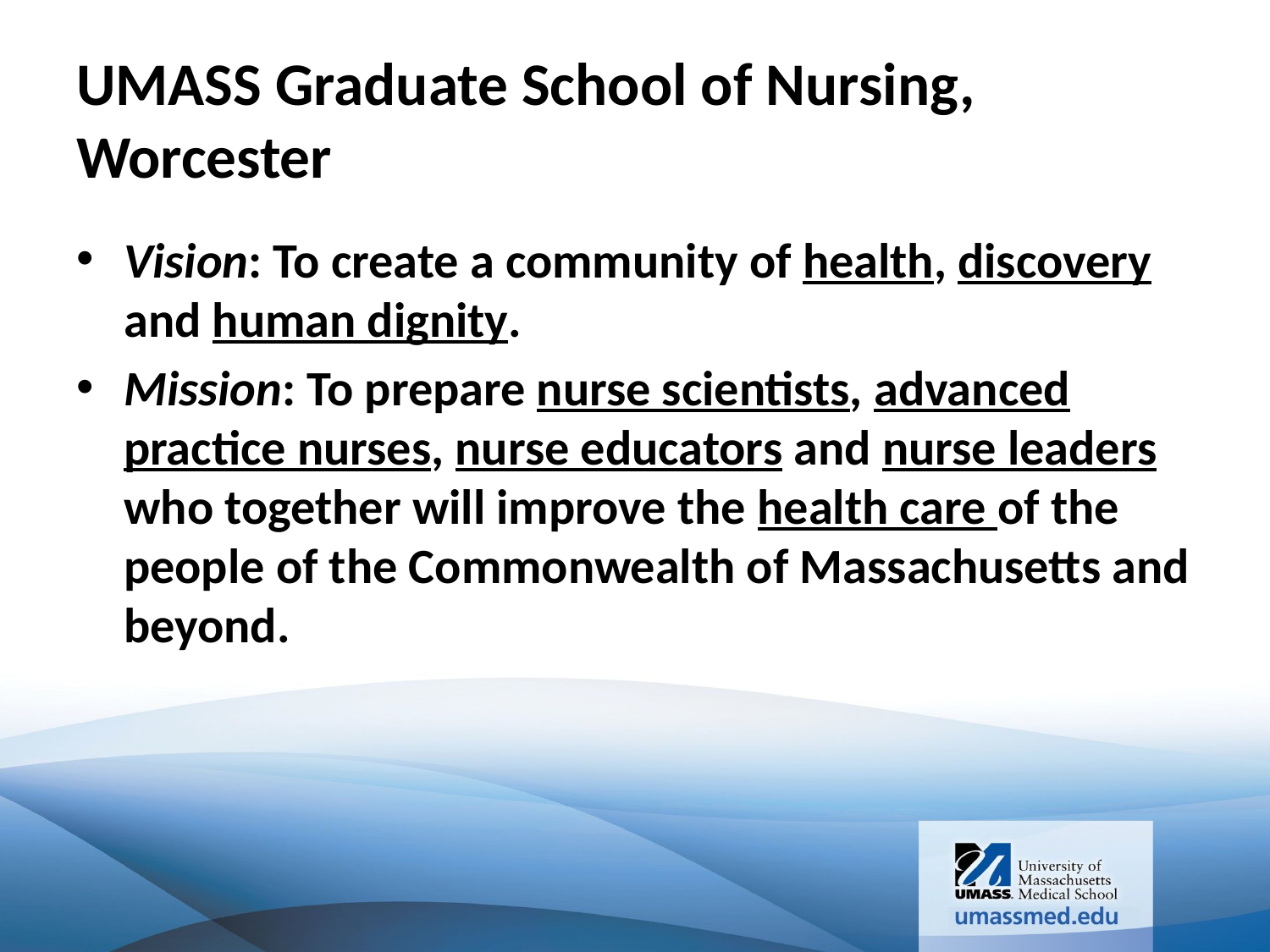

# UMASS Graduate School of Nursing, Worcester
Vision: To create a community of health, discovery and human dignity.
Mission: To prepare nurse scientists, advanced practice nurses, nurse educators and nurse leaders who together will improve the health care of the people of the Commonwealth of Massachusetts and beyond.

## Slide 16
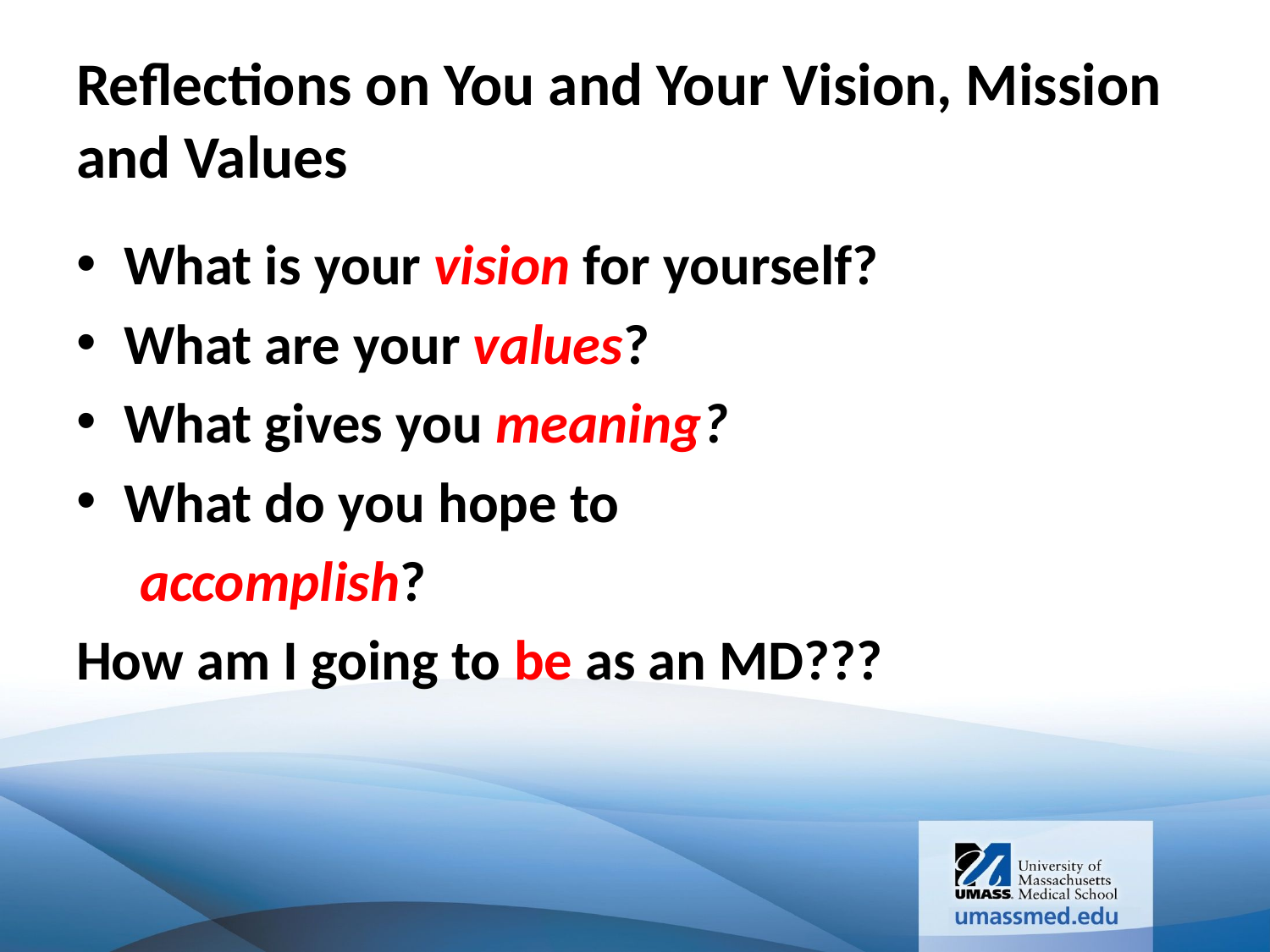

# Reflections on You and Your Vision, Mission and Values
What is your vision for yourself?
What are your values?
What gives you meaning?
What do you hope to
 accomplish?
How am I going to be as an MD???

## Slide 17
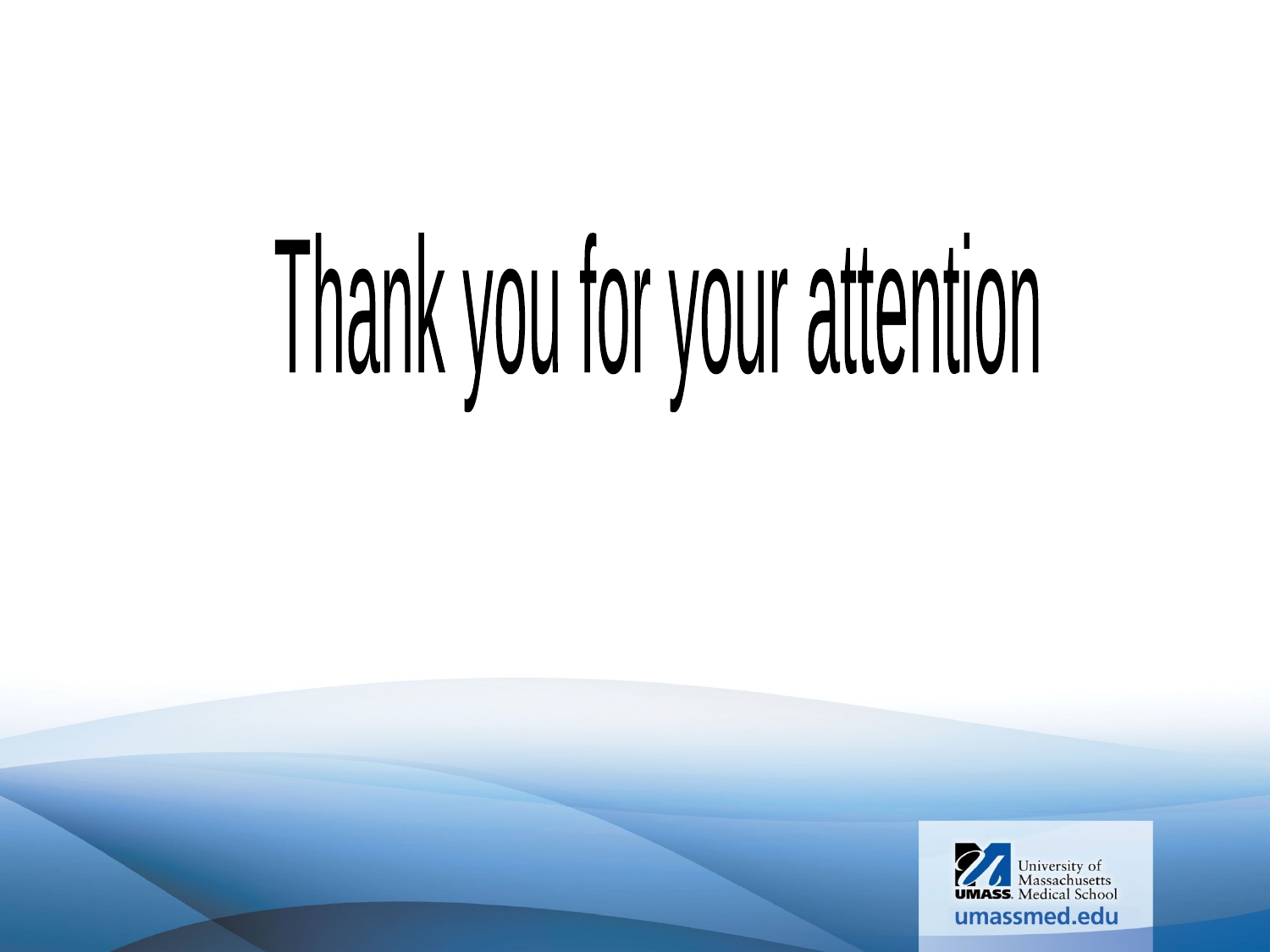

Thank you for your attention
