# Supplementary material for: Preparing Medical Students to Be Physician Leaders: A Leadership Training Program for Students Designed and Led by Students
Source: MedEdPORTAL. 2019 Dec 13;15:10863. doi: 10.15766/mep_2374-8265.10863 (PMC7012310; doi:10.15766/mep_2374-8265.10863)
Supplement: Supplementary file 1 — A. Session 1 PPT Leadership Styles.pptx B. Session 2 PPT Teamwork.pptx C. Session 3 PPT Delegation.pptx D. Session 4 PPT Feedback.pptx E. Session 5 PPT Direction.pptx F. Session 6 Optional Review PPT Consolidation.pptx G. Session 1 Activity Instructions.docx H. Session 2 Activity Instructions.docx I. Session 3 Activity Instructions.docx J. Session 4 Activity Instructions and Figure.docx K. Session 5 Activity Instructions.docx L. Session 6 Activity Instructions.docx M. Precourse and Postcourse Evaluation.docx N. Session 1 Evaluation.docx O. Session 2 Evaluation.docx P. Session 3 Evaluation.docx Q. Session 4 Evaluation.docx R. Session 5 Evaluation.docx S. Posttraining Evaluation.docx T. Supplemental Alternative Activity - PACE Palette.docx U. Supplemental Alternative Activity - ACLS Video.docx V. Supplemental Alternative Activity - Feedback Video.docx [file mep-15-10863-s001.zip › F. Session 6 Optional Review PPT Consolidation.pptx]

## Slide 1
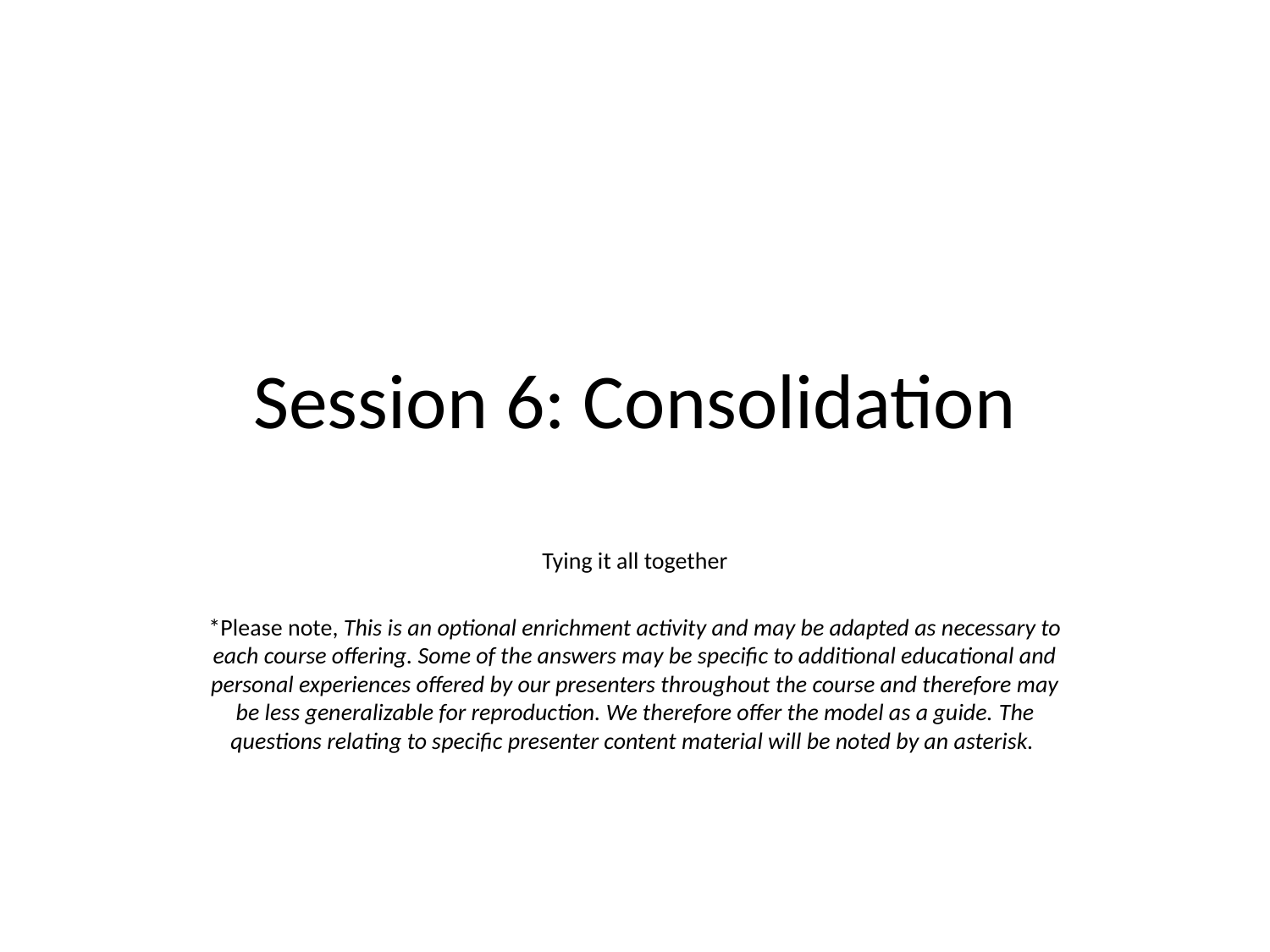

# Session 6: Consolidation
Tying it all together
*Please note, This is an optional enrichment activity and may be adapted as necessary to each course offering. Some of the answers may be specific to additional educational and personal experiences offered by our presenters throughout the course and therefore may be less generalizable for reproduction. We therefore offer the model as a guide. The questions relating to specific presenter content material will be noted by an asterisk.

## Slide 2
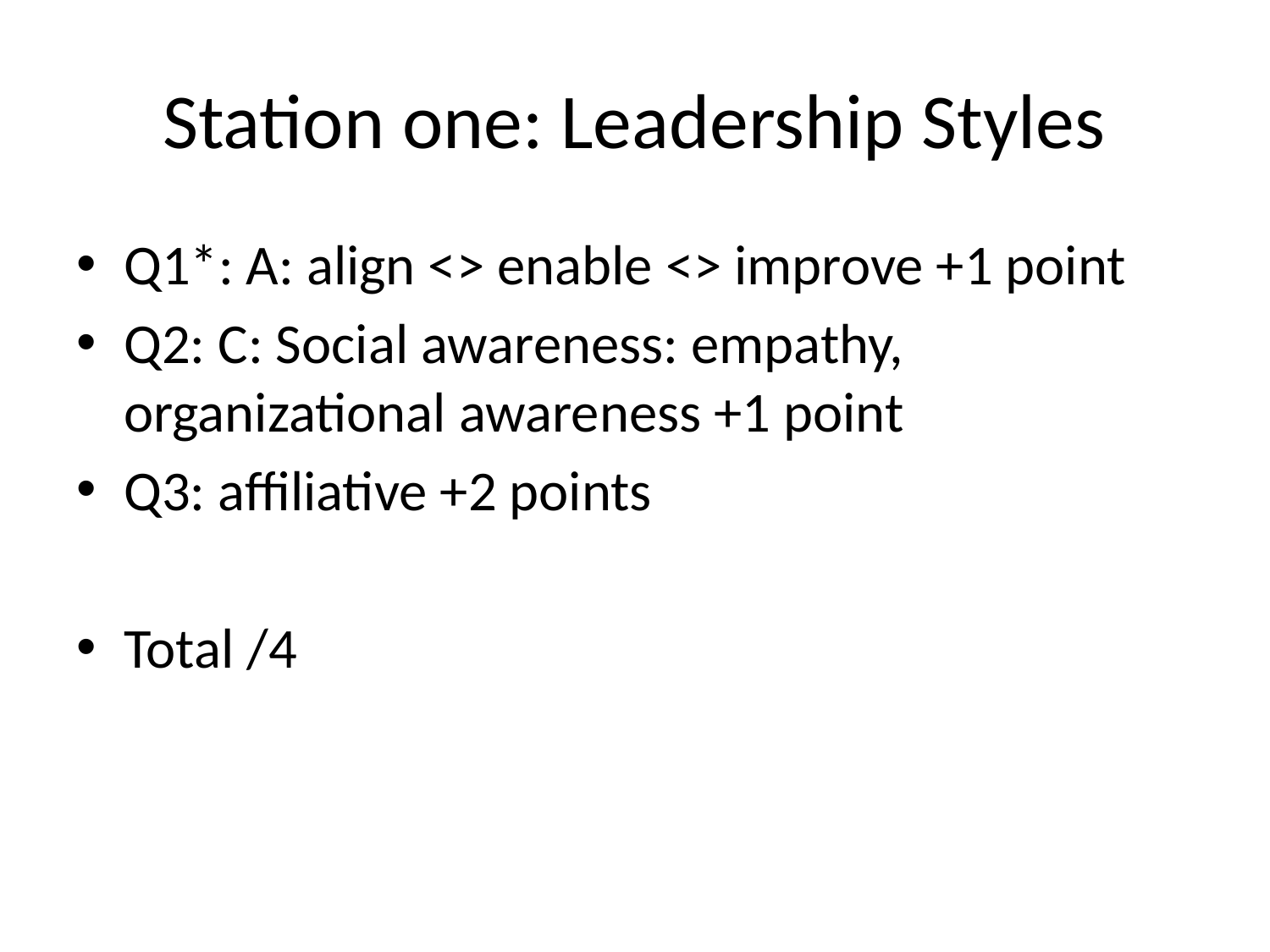

# Station one: Leadership Styles
Q1*: A: align <> enable <> improve +1 point
Q2: C: Social awareness: empathy, organizational awareness +1 point
Q3: affiliative +2 points
Total /4

## Slide 3
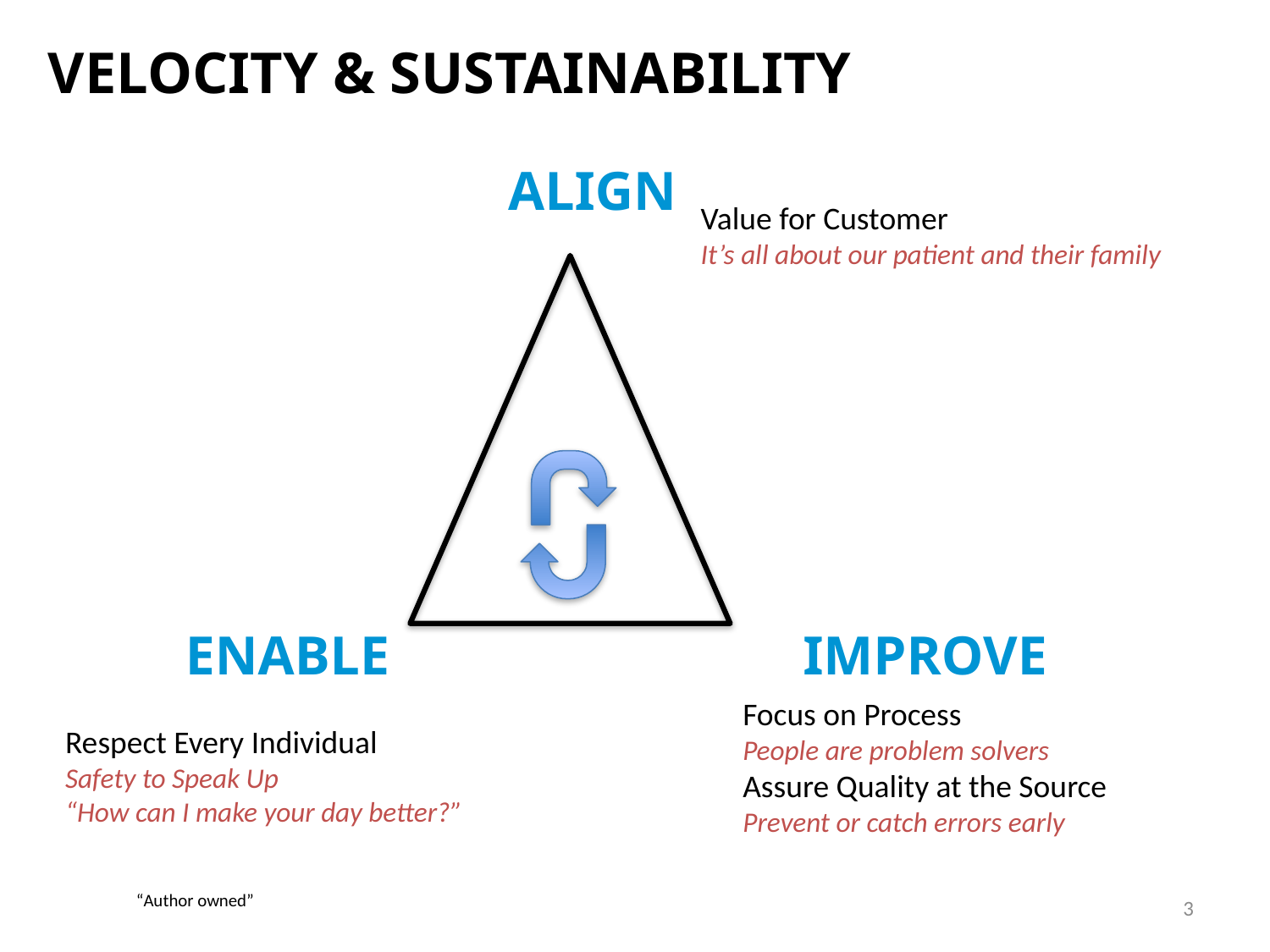

VELOCITY & SUSTAINABILITY
ALIGN
ENABLE
IMPROVE
Value for Customer
It’s all about our patient and their family
Focus on Process
People are problem solvers
Assure Quality at the Source
Prevent or catch errors early
Respect Every Individual
Safety to Speak Up
“How can I make your day better?”
“Author owned”
3

## Slide 4
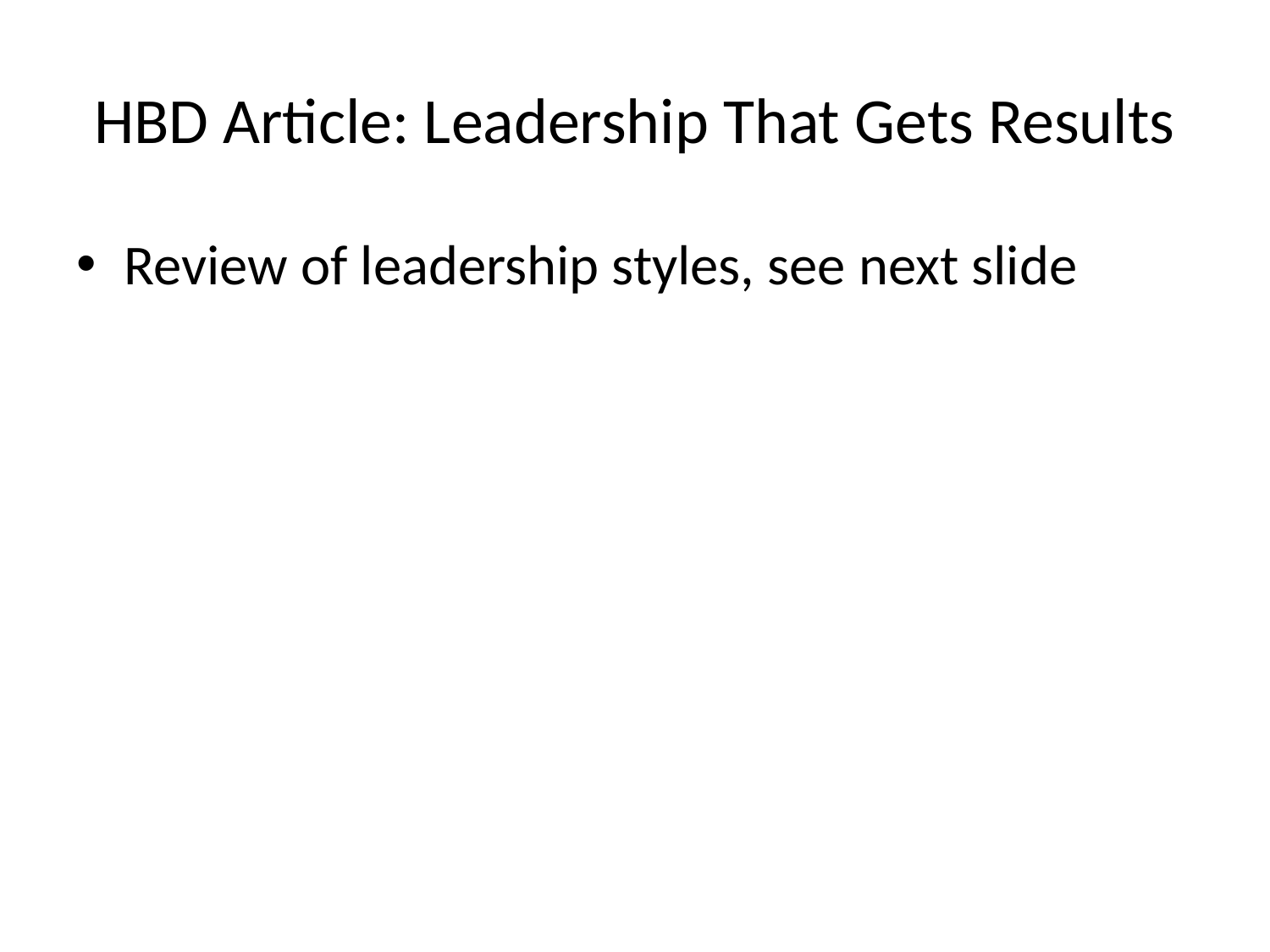

# HBD Article: Leadership That Gets Results
Review of leadership styles, see next slide

## Slide 5
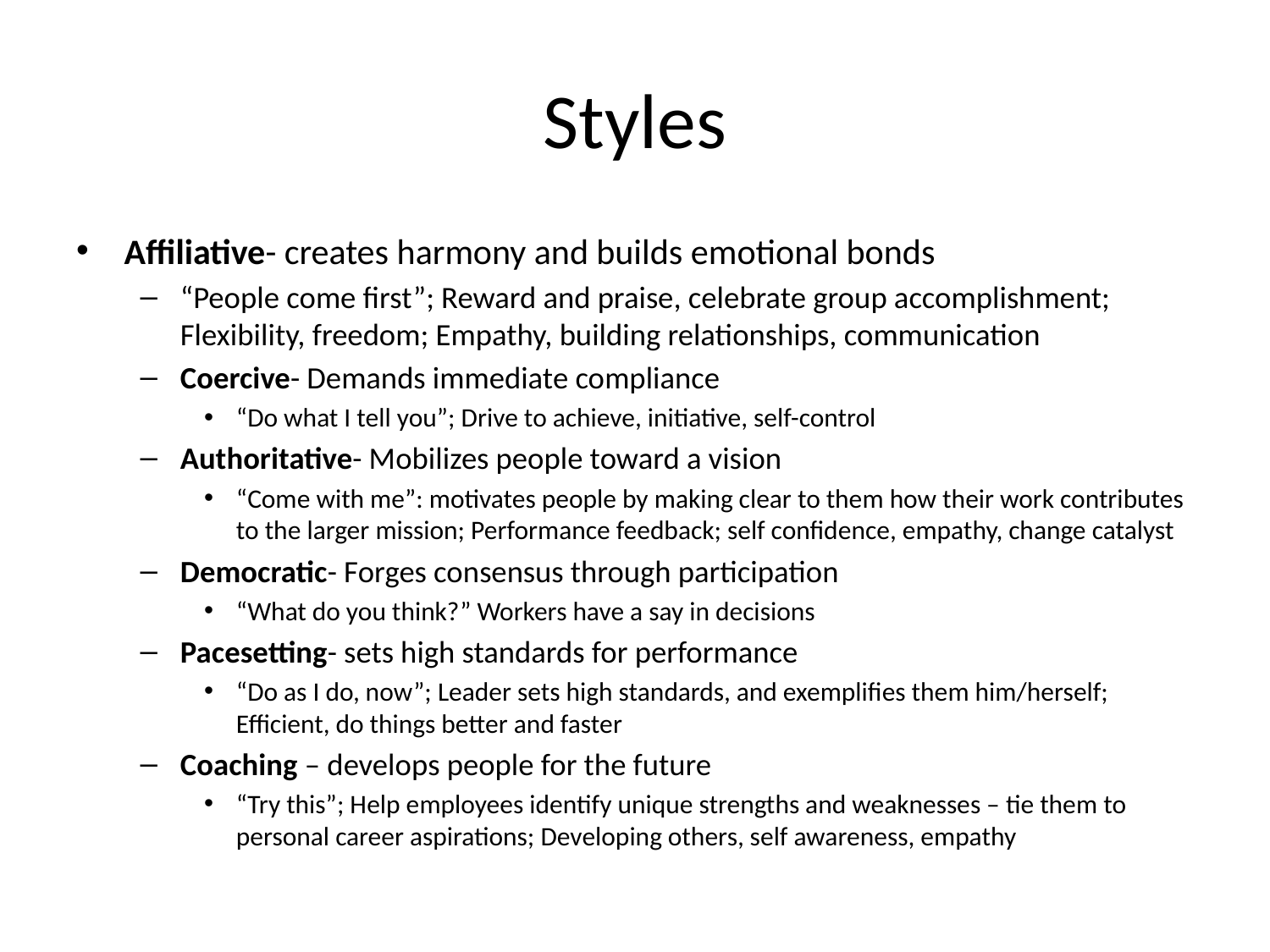

# Styles
Affiliative- creates harmony and builds emotional bonds
“People come first”; Reward and praise, celebrate group accomplishment; Flexibility, freedom; Empathy, building relationships, communication
Coercive- Demands immediate compliance
“Do what I tell you”; Drive to achieve, initiative, self-control
Authoritative- Mobilizes people toward a vision
“Come with me”: motivates people by making clear to them how their work contributes to the larger mission; Performance feedback; self confidence, empathy, change catalyst
Democratic- Forges consensus through participation
“What do you think?” Workers have a say in decisions
Pacesetting- sets high standards for performance
“Do as I do, now”; Leader sets high standards, and exemplifies them him/herself; Efficient, do things better and faster
Coaching – develops people for the future
“Try this”; Help employees identify unique strengths and weaknesses – tie them to personal career aspirations; Developing others, self awareness, empathy

## Slide 6
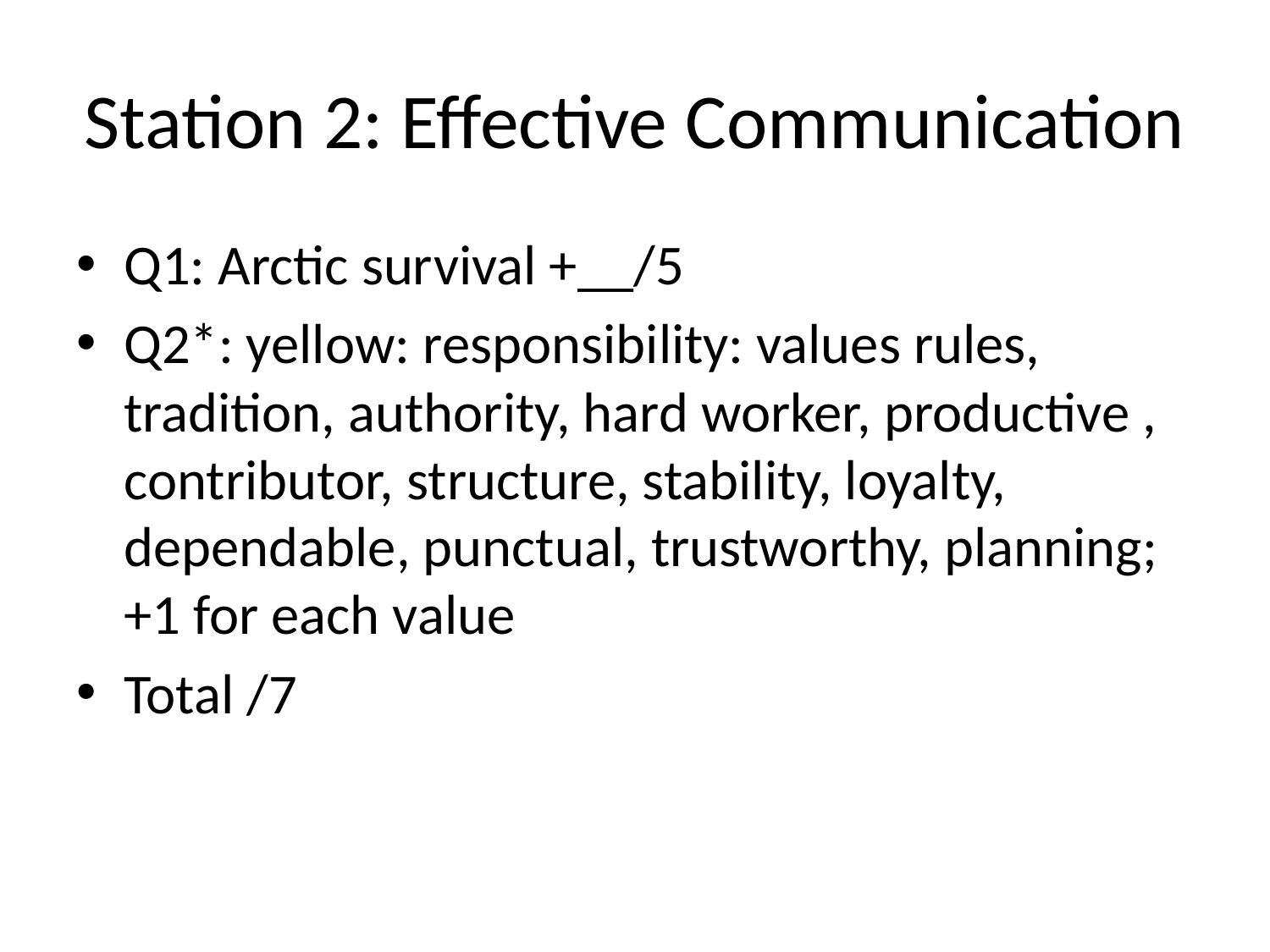

# Station 2: Effective Communication
Q1: Arctic survival +__/5
Q2*: yellow: responsibility: values rules, tradition, authority, hard worker, productive , contributor, structure, stability, loyalty, dependable, punctual, trustworthy, planning; +1 for each value
Total /7

## Slide 7
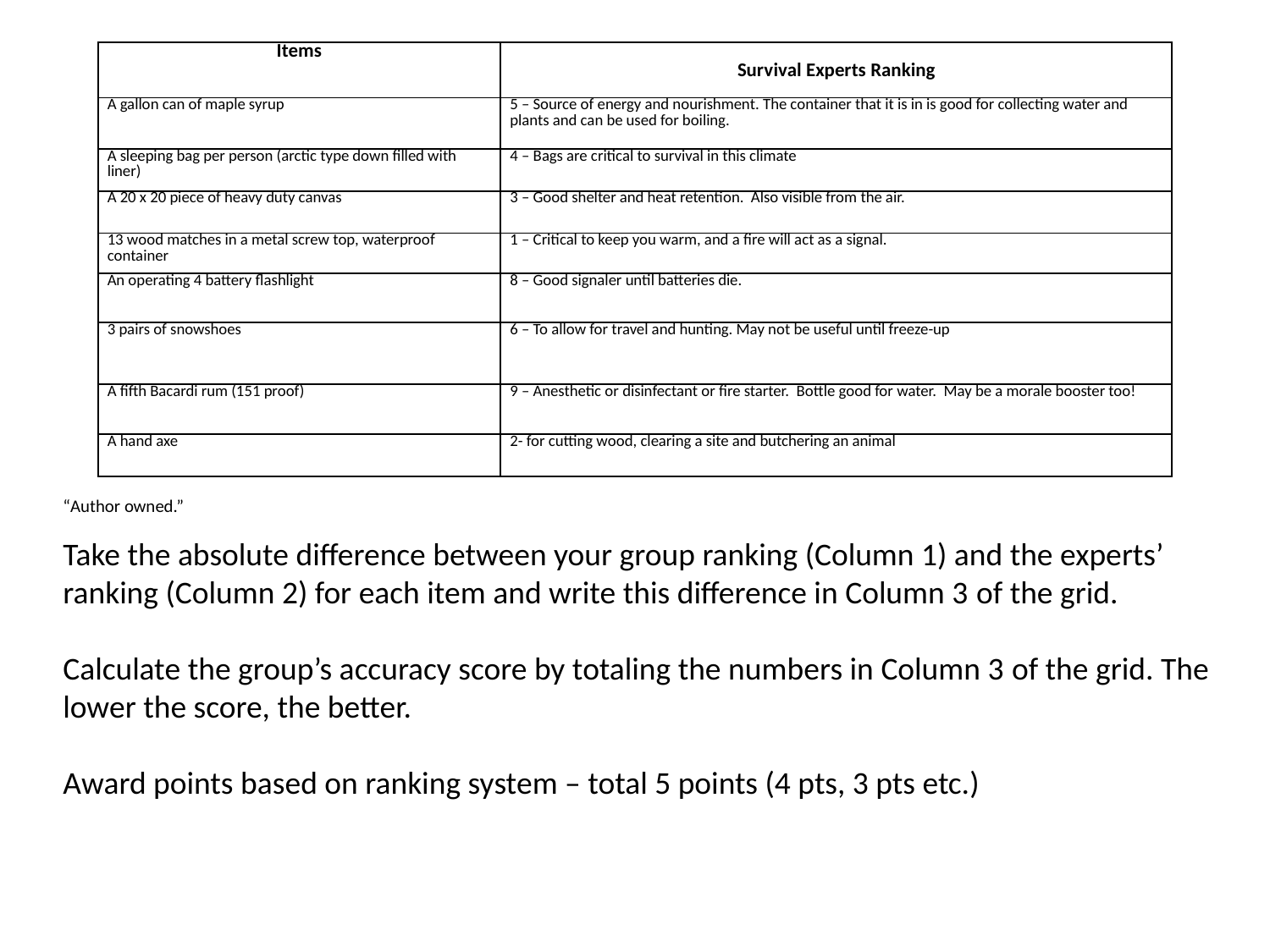

| Items | Survival Experts Ranking |
| --- | --- |
| A gallon can of maple syrup | 5 – Source of energy and nourishment. The container that it is in is good for collecting water and plants and can be used for boiling. |
| A sleeping bag per person (arctic type down filled with liner) | 4 – Bags are critical to survival in this climate |
| A 20 x 20 piece of heavy duty canvas | 3 – Good shelter and heat retention. Also visible from the air. |
| 13 wood matches in a metal screw top, waterproof container | 1 – Critical to keep you warm, and a fire will act as a signal. |
| An operating 4 battery flashlight | 8 – Good signaler until batteries die. |
| 3 pairs of snowshoes | 6 – To allow for travel and hunting. May not be useful until freeze-up |
| A fifth Bacardi rum (151 proof) | 9 – Anesthetic or disinfectant or fire starter. Bottle good for water. May be a morale booster too! |
| A hand axe | 2- for cutting wood, clearing a site and butchering an animal |
“Author owned.”
Take the absolute difference between your group ranking (Column 1) and the experts’ ranking (Column 2) for each item and write this difference in Column 3 of the grid.
Calculate the group’s accuracy score by totaling the numbers in Column 3 of the grid. The lower the score, the better.
Award points based on ranking system – total 5 points (4 pts, 3 pts etc.)

## Slide 8
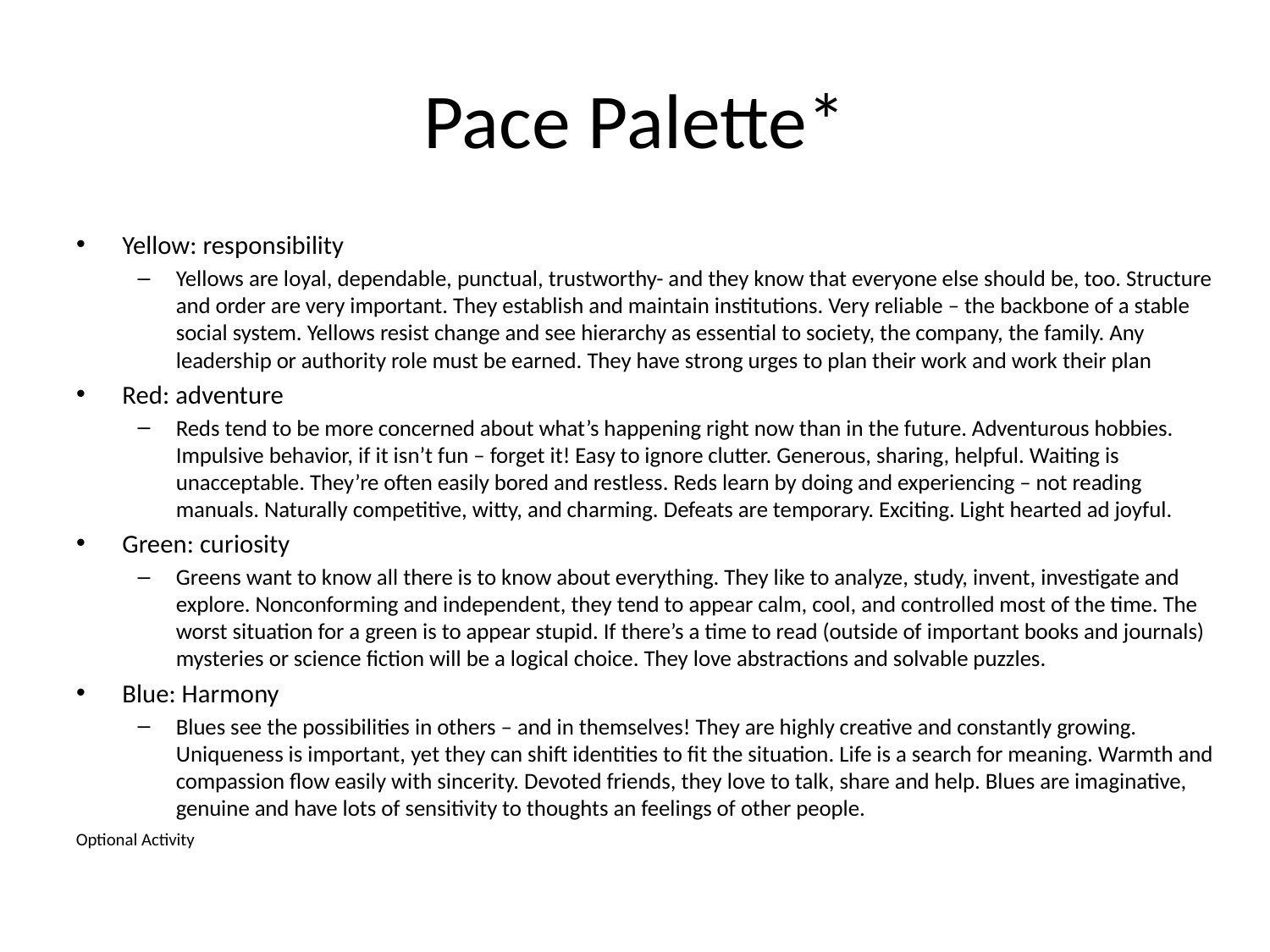

# Pace Palette*
Yellow: responsibility
Yellows are loyal, dependable, punctual, trustworthy- and they know that everyone else should be, too. Structure and order are very important. They establish and maintain institutions. Very reliable – the backbone of a stable social system. Yellows resist change and see hierarchy as essential to society, the company, the family. Any leadership or authority role must be earned. They have strong urges to plan their work and work their plan
Red: adventure
Reds tend to be more concerned about what’s happening right now than in the future. Adventurous hobbies. Impulsive behavior, if it isn’t fun – forget it! Easy to ignore clutter. Generous, sharing, helpful. Waiting is unacceptable. They’re often easily bored and restless. Reds learn by doing and experiencing – not reading manuals. Naturally competitive, witty, and charming. Defeats are temporary. Exciting. Light hearted ad joyful.
Green: curiosity
Greens want to know all there is to know about everything. They like to analyze, study, invent, investigate and explore. Nonconforming and independent, they tend to appear calm, cool, and controlled most of the time. The worst situation for a green is to appear stupid. If there’s a time to read (outside of important books and journals) mysteries or science fiction will be a logical choice. They love abstractions and solvable puzzles.
Blue: Harmony
Blues see the possibilities in others – and in themselves! They are highly creative and constantly growing. Uniqueness is important, yet they can shift identities to fit the situation. Life is a search for meaning. Warmth and compassion flow easily with sincerity. Devoted friends, they love to talk, share and help. Blues are imaginative, genuine and have lots of sensitivity to thoughts an feelings of other people.
Optional Activity

## Slide 9
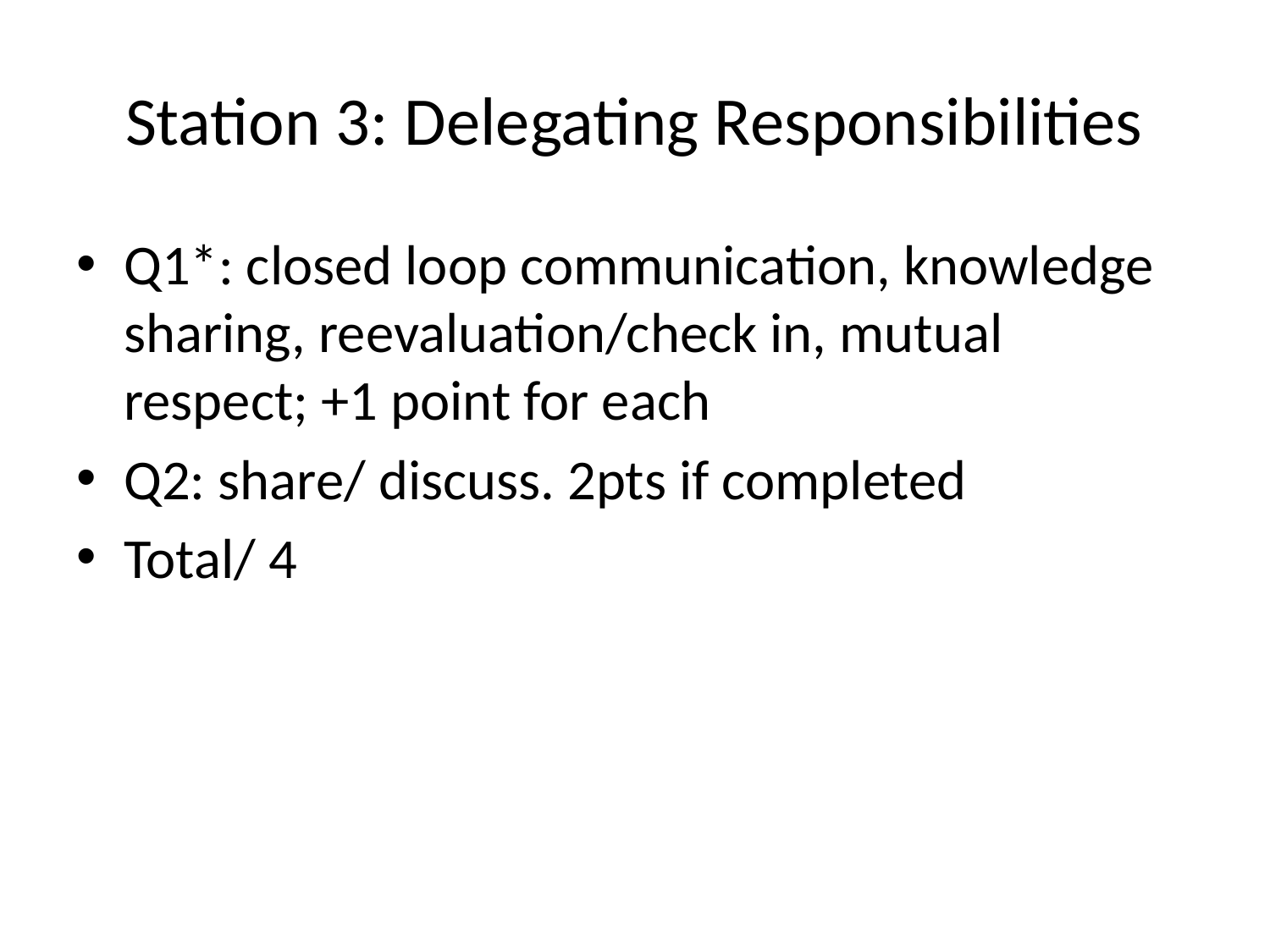

# Station 3: Delegating Responsibilities
Q1*: closed loop communication, knowledge sharing, reevaluation/check in, mutual respect; +1 point for each
Q2: share/ discuss. 2pts if completed
Total/ 4

## Slide 10
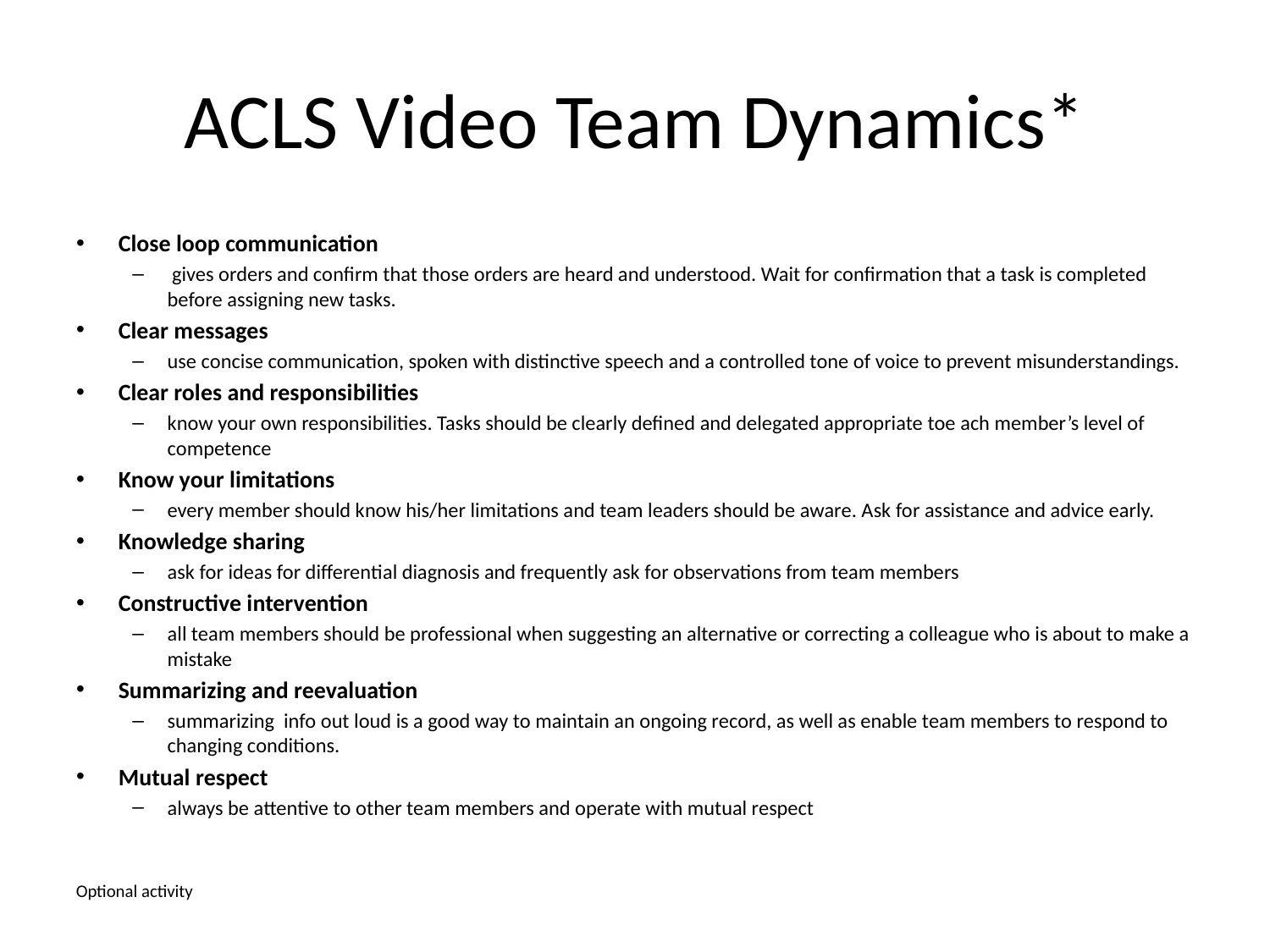

# ACLS Video Team Dynamics*
Close loop communication
 gives orders and confirm that those orders are heard and understood. Wait for confirmation that a task is completed before assigning new tasks.
Clear messages
use concise communication, spoken with distinctive speech and a controlled tone of voice to prevent misunderstandings.
Clear roles and responsibilities
know your own responsibilities. Tasks should be clearly defined and delegated appropriate toe ach member’s level of competence
Know your limitations
every member should know his/her limitations and team leaders should be aware. Ask for assistance and advice early.
Knowledge sharing
ask for ideas for differential diagnosis and frequently ask for observations from team members
Constructive intervention
all team members should be professional when suggesting an alternative or correcting a colleague who is about to make a mistake
Summarizing and reevaluation
summarizing info out loud is a good way to maintain an ongoing record, as well as enable team members to respond to changing conditions.
Mutual respect
always be attentive to other team members and operate with mutual respect
Optional activity

## Slide 11
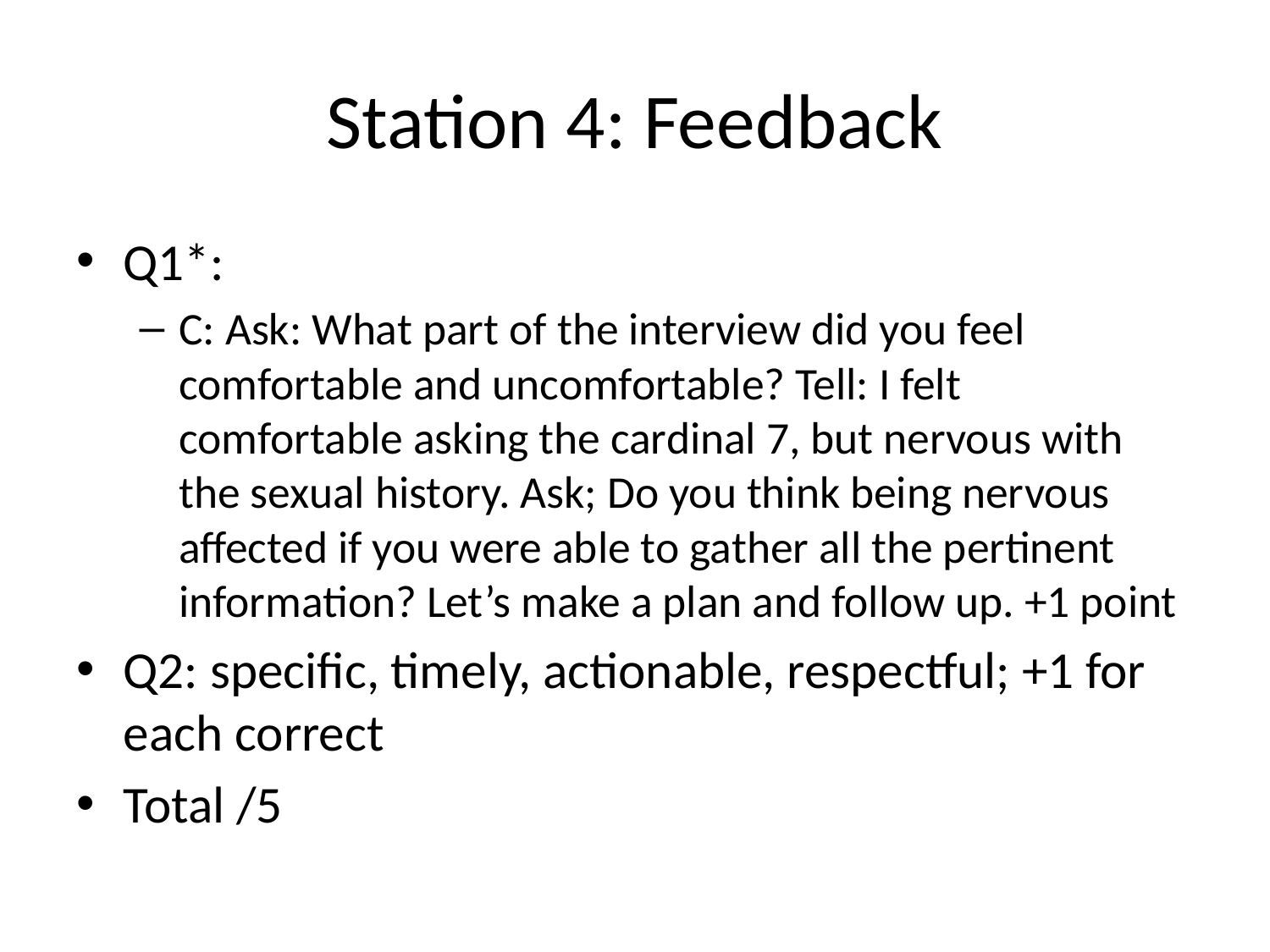

# Station 4: Feedback
Q1*:
C: Ask: What part of the interview did you feel comfortable and uncomfortable? Tell: I felt comfortable asking the cardinal 7, but nervous with the sexual history. Ask; Do you think being nervous affected if you were able to gather all the pertinent information? Let’s make a plan and follow up. +1 point
Q2: specific, timely, actionable, respectful; +1 for each correct
Total /5

## Slide 12
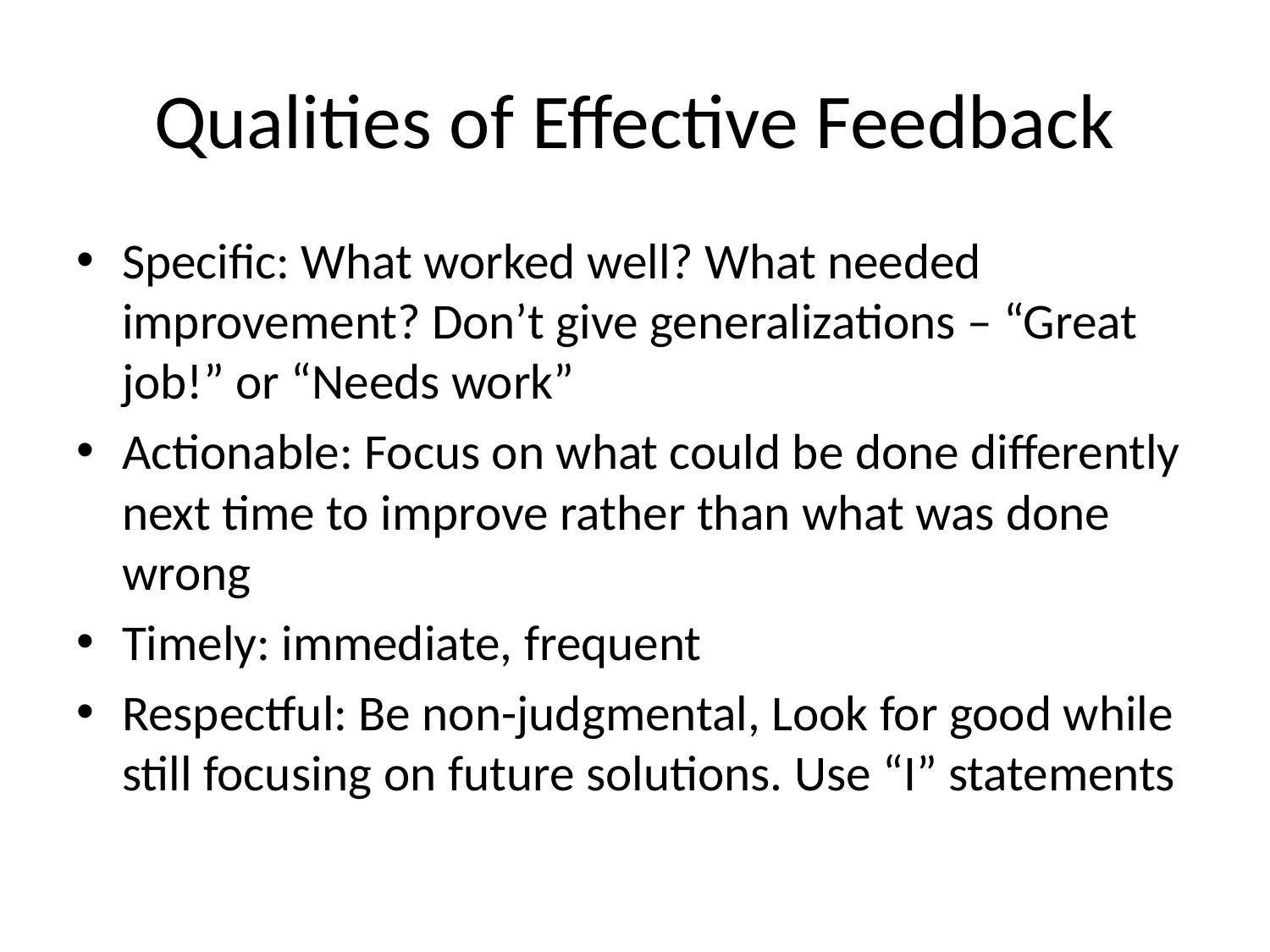

# Qualities of Effective Feedback
Specific: What worked well? What needed improvement? Don’t give generalizations – “Great job!” or “Needs work”
Actionable: Focus on what could be done differently next time to improve rather than what was done wrong
Timely: immediate, frequent
Respectful: Be non-judgmental, Look for good while still focusing on future solutions. Use “I” statements

## Slide 13
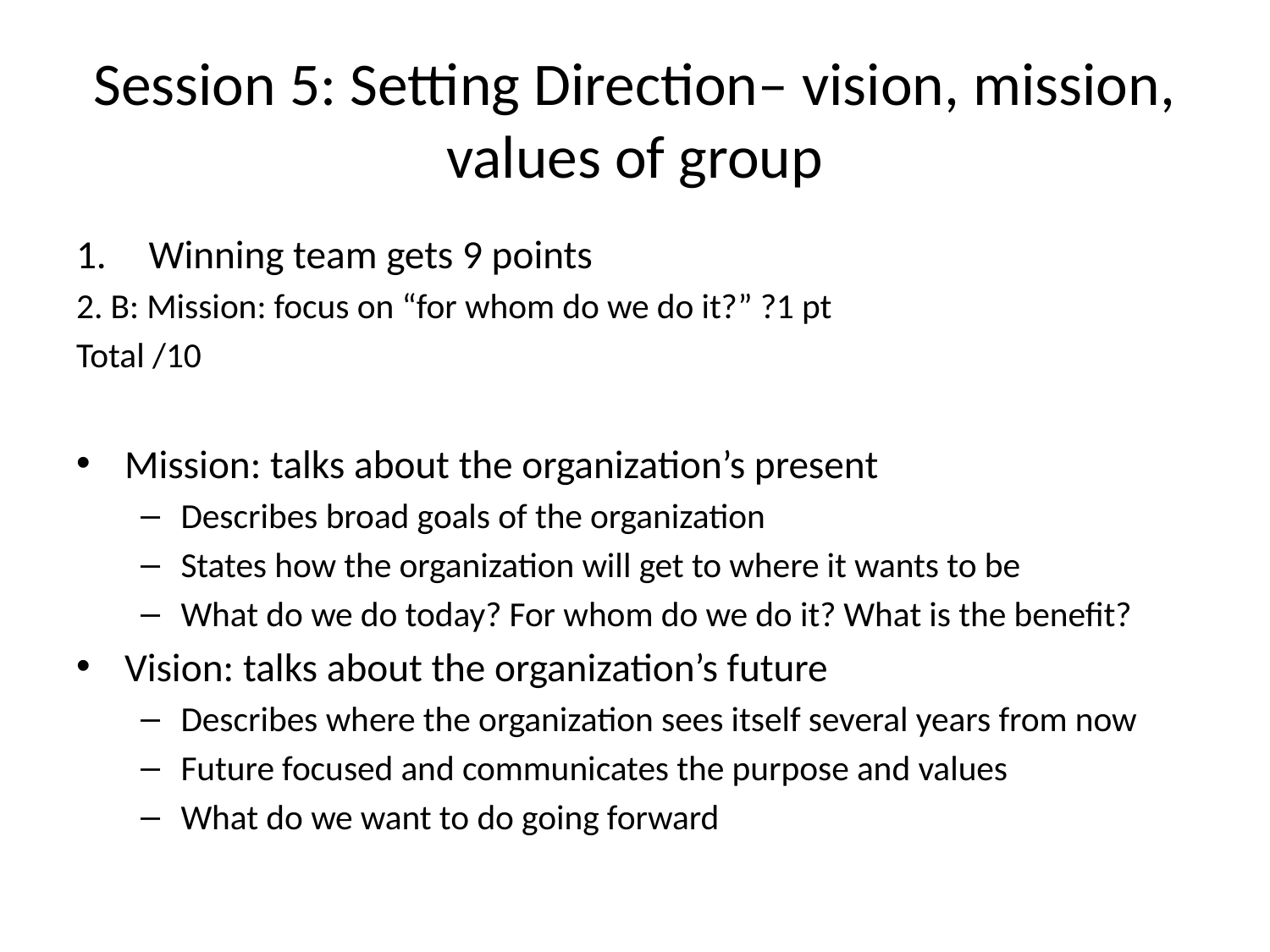

# Session 5: Setting Direction– vision, mission, values of group
Winning team gets 9 points
2. B: Mission: focus on “for whom do we do it?” ?1 pt
Total /10
Mission: talks about the organization’s present
Describes broad goals of the organization
States how the organization will get to where it wants to be
What do we do today? For whom do we do it? What is the benefit?
Vision: talks about the organization’s future
Describes where the organization sees itself several years from now
Future focused and communicates the purpose and values
What do we want to do going forward

## Slide 14
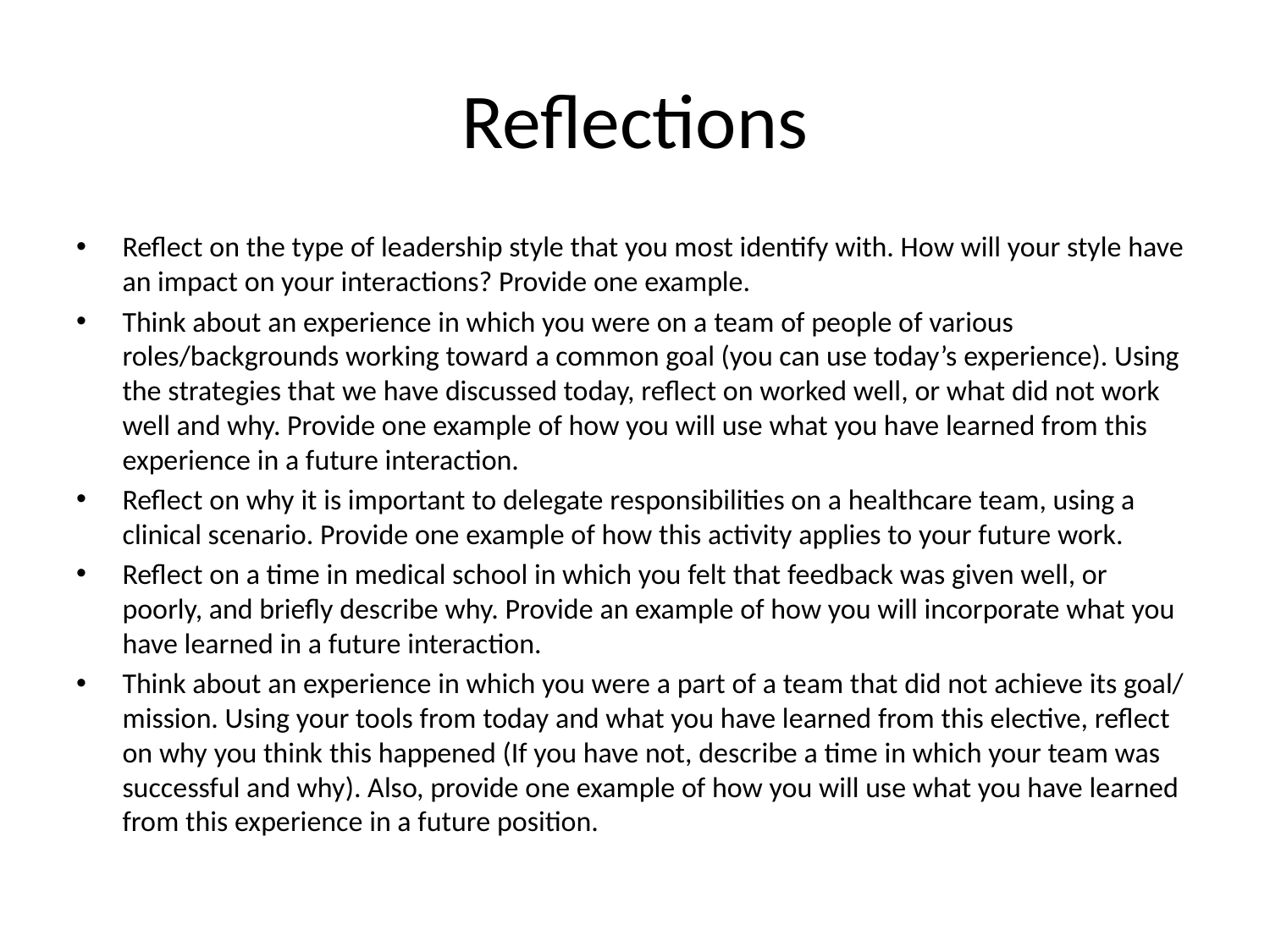

# Reflections
Reflect on the type of leadership style that you most identify with. How will your style have an impact on your interactions? Provide one example.
Think about an experience in which you were on a team of people of various roles/backgrounds working toward a common goal (you can use today’s experience). Using the strategies that we have discussed today, reflect on worked well, or what did not work well and why. Provide one example of how you will use what you have learned from this experience in a future interaction.
Reflect on why it is important to delegate responsibilities on a healthcare team, using a clinical scenario. Provide one example of how this activity applies to your future work.
Reflect on a time in medical school in which you felt that feedback was given well, or poorly, and briefly describe why. Provide an example of how you will incorporate what you have learned in a future interaction.
Think about an experience in which you were a part of a team that did not achieve its goal/ mission. Using your tools from today and what you have learned from this elective, reflect on why you think this happened (If you have not, describe a time in which your team was successful and why). Also, provide one example of how you will use what you have learned from this experience in a future position.

## Slide 15
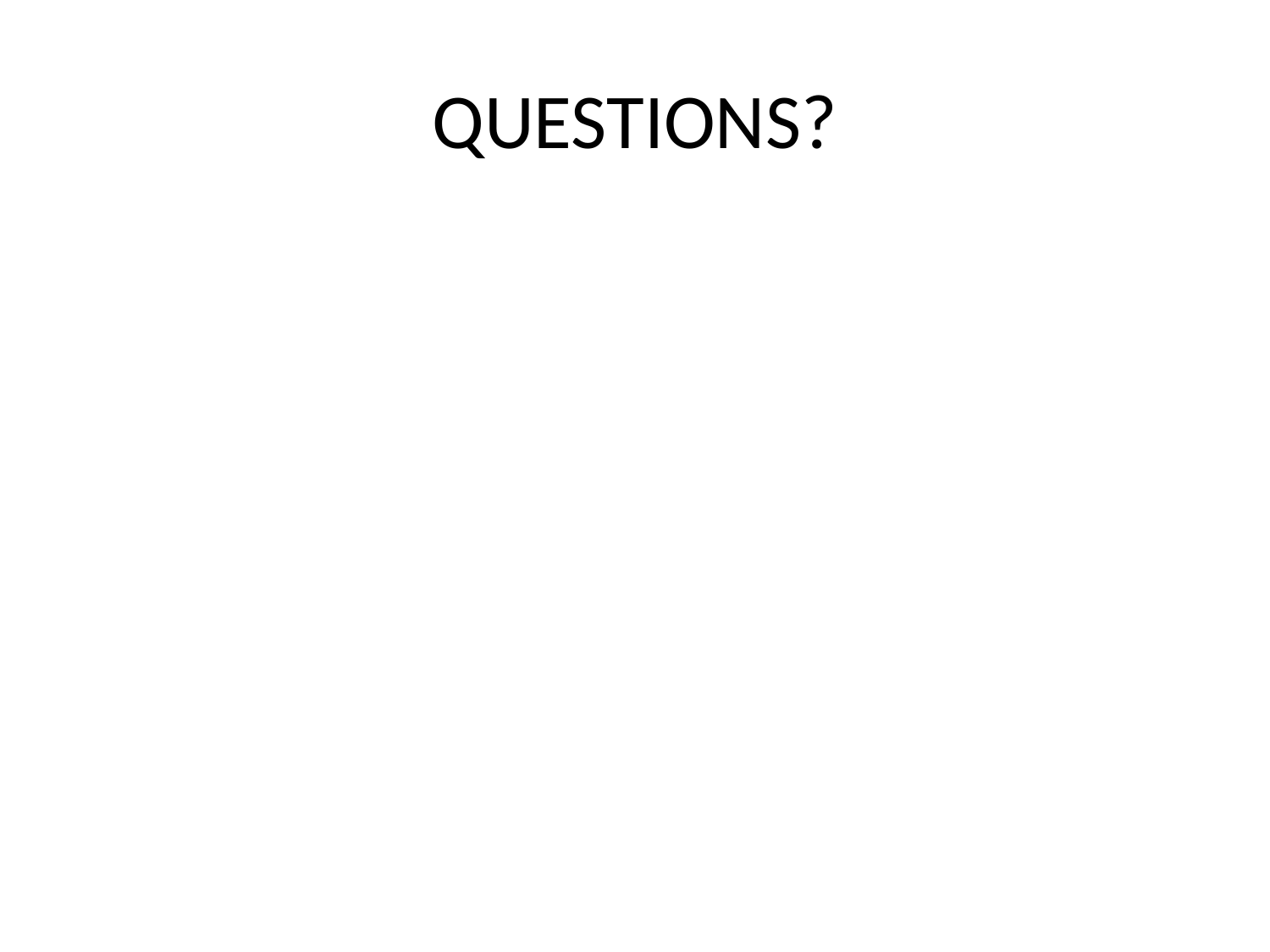

# QUESTIONS?
