# Supplementary material for: Preparing Medical Students to Be Physician Leaders: A Leadership Training Program for Students Designed and Led by Students
Source: MedEdPORTAL. 2019 Dec 13;15:10863. doi: 10.15766/mep_2374-8265.10863 (PMC7012310; doi:10.15766/mep_2374-8265.10863)
Supplement: Supplementary file 1 — A. Session 1 PPT Leadership Styles.pptx B. Session 2 PPT Teamwork.pptx C. Session 3 PPT Delegation.pptx D. Session 4 PPT Feedback.pptx E. Session 5 PPT Direction.pptx F. Session 6 Optional Review PPT Consolidation.pptx G. Session 1 Activity Instructions.docx H. Session 2 Activity Instructions.docx I. Session 3 Activity Instructions.docx J. Session 4 Activity Instructions and Figure.docx K. Session 5 Activity Instructions.docx L. Session 6 Activity Instructions.docx M. Precourse and Postcourse Evaluation.docx N. Session 1 Evaluation.docx O. Session 2 Evaluation.docx P. Session 3 Evaluation.docx Q. Session 4 Evaluation.docx R. Session 5 Evaluation.docx S. Posttraining Evaluation.docx T. Supplemental Alternative Activity - PACE Palette.docx U. Supplemental Alternative Activity - ACLS Video.docx V. Supplemental Alternative Activity - Feedback Video.docx [file mep-15-10863-s001.zip › G. Session 1 Activity Instructions.docx]

Leadership Style Scenario

*Activity to be performed following introduction to leadership styles, refer to Appendix A: PowerPoint to Session 1, Leadership Styles*

Objectives: Practice utilizing various styles of leadership and identify which leadership competencies are necessary to be successful leaders in various types of situations.

Materials: none

Time: Approximately 20 minutes, depending on number of groups

Instructions:

1. Break up participants into small groups (2-4 participant).
2. Present a specific scenario to the groups. The scenario is a team that is faced with a specific challenge. Each team will be assigned one of the leadership styles to portray.
3. Take turns acting out scenarios. The audience will have to then guess the style.
4. Discuss as a group the characteristics of the leadership style that worked well/ did not work well for he scenario.

Directions for participants: As a team, create the above role playing scenario demonstrating outcome/s of your assigned leadership style. Develop roles for each player and portray the appropriate interactions. If possible, include both positive and negative aspects of the style. Scenario length 3-5 minutes

Example scenarios

- You are students voting on school policy for uniforms
- You are congress voting on town law to raise/lower driving age
- You are townspeople voting on homes allowing pets
- You are students voting on banning homework
- Etc.
