# Supplementary material for: Preparing Medical Students to Be Physician Leaders: A Leadership Training Program for Students Designed and Led by Students
Source: MedEdPORTAL. 2019 Dec 13;15:10863. doi: 10.15766/mep_2374-8265.10863 (PMC7012310; doi:10.15766/mep_2374-8265.10863)
Supplement: Supplementary file 1 — A. Session 1 PPT Leadership Styles.pptx B. Session 2 PPT Teamwork.pptx C. Session 3 PPT Delegation.pptx D. Session 4 PPT Feedback.pptx E. Session 5 PPT Direction.pptx F. Session 6 Optional Review PPT Consolidation.pptx G. Session 1 Activity Instructions.docx H. Session 2 Activity Instructions.docx I. Session 3 Activity Instructions.docx J. Session 4 Activity Instructions and Figure.docx K. Session 5 Activity Instructions.docx L. Session 6 Activity Instructions.docx M. Precourse and Postcourse Evaluation.docx N. Session 1 Evaluation.docx O. Session 2 Evaluation.docx P. Session 3 Evaluation.docx Q. Session 4 Evaluation.docx R. Session 5 Evaluation.docx S. Posttraining Evaluation.docx T. Supplemental Alternative Activity - PACE Palette.docx U. Supplemental Alternative Activity - ACLS Video.docx V. Supplemental Alternative Activity - Feedback Video.docx [file mep-15-10863-s001.zip › H. Session 2 Activity Instructions.docx]

Teamwork

*Activity to be performed following introduction to teamwork and communication, refer to Appendix B: PowerPoint to Session 2, Teamwork*

Objectives: In this activity, team members trade pieces of playing cards to put together complete cards. This exercise is useful for showing team members others' perspectives. It builds communication and [negotiation skills](https://www.mindtools.com/CommSkll/NegotiationSkills.htm), and helps to develop insight into another’s reasoning.

People and Materials:

- Enough people for at least three teams of two.
- One deck of cards. Playing cards – use between four and six for each person.
- A private room.

Time:

- 15 minutes.

Instructions:

1. Cut each playing card into half diagonally, then in half diagonally again, so you have four triangular pieces for each card.
2. Mix all the pieces together and put equal numbers of cards into as many envelopes as you have teams.
3. Divide people up into teams of three or four. You need at least three teams. If you're short of people, teams of two will work just as well.
4. Give each team an envelope of playing card pieces.
5. Each team has three minutes to sort its pieces, determine which ones it needs to make complete cards, and develop a bargaining strategy.
6. After three minutes, allow the teams to start bartering for pieces. People can barter on their own or collectively with their team. Give the teams eight minutes to barter.
7. When the time is up, count each team's completed cards. Whichever team has the most cards wins the round.

Advice for the Facilitator

After the activity, ask your team members to think about the strategies they used. Discuss these questions:

- Which negotiation strategies worked? Which didn't?
- What could they have done better?
- What other skills, such as [active listening](https://www.mindtools.com/CommSkll/ActiveListening.htm)  or [empathy](https://www.mindtools.com/pages/article/newLDR_75.htm) , did they need to use?
