# Supplementary material for: Preparing Medical Students to Be Physician Leaders: A Leadership Training Program for Students Designed and Led by Students
Source: MedEdPORTAL. 2019 Dec 13;15:10863. doi: 10.15766/mep_2374-8265.10863 (PMC7012310; doi:10.15766/mep_2374-8265.10863)
Supplement: Supplementary file 1 — A. Session 1 PPT Leadership Styles.pptx B. Session 2 PPT Teamwork.pptx C. Session 3 PPT Delegation.pptx D. Session 4 PPT Feedback.pptx E. Session 5 PPT Direction.pptx F. Session 6 Optional Review PPT Consolidation.pptx G. Session 1 Activity Instructions.docx H. Session 2 Activity Instructions.docx I. Session 3 Activity Instructions.docx J. Session 4 Activity Instructions and Figure.docx K. Session 5 Activity Instructions.docx L. Session 6 Activity Instructions.docx M. Precourse and Postcourse Evaluation.docx N. Session 1 Evaluation.docx O. Session 2 Evaluation.docx P. Session 3 Evaluation.docx Q. Session 4 Evaluation.docx R. Session 5 Evaluation.docx S. Posttraining Evaluation.docx T. Supplemental Alternative Activity - PACE Palette.docx U. Supplemental Alternative Activity - ACLS Video.docx V. Supplemental Alternative Activity - Feedback Video.docx [file mep-15-10863-s001.zip › I. Session 3 Activity Instructions.docx]

Delegation Exercise

*Activity to be performed following introduction to delegation skill, refer to Appendix C: PowerPoint to Session 3, Delegation*

Objective: Participants practice formulating a clear objective through a team activity and practice formulating clear instructions through a team abuilding activity, in clearly defined roles.

Materials: Multiple matching sets of children’s building blocks (e.g. Lego, or colored plastic cups), with 10 blocks in each set. Optional: jelly beans, tooth pics, popsicle sticks, etc. (Note, can improvise building materials: we used colored plastic cups with colored jellybeans in each cup, as well as popsicle sticks, to create a tower to stacked plastic cups)

Preparation: Using one set of blocks, build a random object using the 10 blocks.

*Group Size*– minimum 3 people
(You can have duplicate exercise running in parallel if group is larger, but will need more sets of building blocks).

There are 4 roles in this communication skills game.

Person A – director
Person B – runner
Person C – builder
Person(s) D – observer(s)

Directions:

1. Person A is given the built-up set of blocks, and is the only person who can see the object. It is the director’s job to give clear instructions to person B, the runner, so that person C can build an exact replica of the model.
2. Person B listens to the director’s instructions and runs to a different part of the room to where person C is sitting. The runner then passes on the building instructions, without seeing the building blocks, to Person C, the builder. The runner can make as many trips as required within the time allowed for the exercise.
3. Person C listens to the runner’s instructions and builds the object from the set of building blocks. The builder is the only person who can see the object under construction, and building materials.
4. Person(s) D observe the communication game, and make notes about what works, what doesn’t work, and how people behaved under pressure etc., to pass onto the group later.

Time: Set a time limit for the exercise of 10 minutes.

When the time is up, allow the group to compare the model and the replica, and see how closely it matches. Generally, the replica will bear little resemblance to the original, which usually causes heated discussion!

- Allow the group to reflect on how the exercise went, and agree 1 thing they did well, 1 thing that didn’t work, and 1 thing they would do better next time.

May run the exercise again, either switching or keeping original roles, and see if any improvements have been made. Make sure to de-construct the “original” model and create a new design!

This simple communication skills game can be run many times without losing learning potential. Teams can add layers of sophistication to their communication by making use of aids such as diagrams, codes, standard procedures and using active listening techniques. (could block the runner's path, change the time limit, etc.)

Points to address in discussion:

1. Closed loop communication: Did the director start by providing a clear objectives or vision of the model before the building process? Were instructions clear? Did the runner ask for clarifying information when instructions were not clear? Did the builder ask for clarifying instructions when the instructions were not clear?
2. Knowledge sharing: Did the builder as for a clear vision of the model at any point? Was the team on the same page regarding the model they were trying to build?
3. Re evaluate/check in: Did the director ask the runner about how the building was going?
4. Mutual respect: Did everyone stay calm and cooperative? Were there any respect issues?
