# Supplementary material for: Preparing Medical Students to Be Physician Leaders: A Leadership Training Program for Students Designed and Led by Students
Source: MedEdPORTAL. 2019 Dec 13;15:10863. doi: 10.15766/mep_2374-8265.10863 (PMC7012310; doi:10.15766/mep_2374-8265.10863)
Supplement: Supplementary file 1 — A. Session 1 PPT Leadership Styles.pptx B. Session 2 PPT Teamwork.pptx C. Session 3 PPT Delegation.pptx D. Session 4 PPT Feedback.pptx E. Session 5 PPT Direction.pptx F. Session 6 Optional Review PPT Consolidation.pptx G. Session 1 Activity Instructions.docx H. Session 2 Activity Instructions.docx I. Session 3 Activity Instructions.docx J. Session 4 Activity Instructions and Figure.docx K. Session 5 Activity Instructions.docx L. Session 6 Activity Instructions.docx M. Precourse and Postcourse Evaluation.docx N. Session 1 Evaluation.docx O. Session 2 Evaluation.docx P. Session 3 Evaluation.docx Q. Session 4 Evaluation.docx R. Session 5 Evaluation.docx S. Posttraining Evaluation.docx T. Supplemental Alternative Activity - PACE Palette.docx U. Supplemental Alternative Activity - ACLS Video.docx V. Supplemental Alternative Activity - Feedback Video.docx [file mep-15-10863-s001.zip › J. Session 4 Activity Instructions and Figure.docx]

**Feedback games:** The feedback figure

*Activity to be performed following introduction to giving and receiving feedback, refer to Appendix D: PowerPoint to Session 4, Feedback*

Objectives: This feedback game is used to demonstrate the value of giving clear instructions as well as practicing giving feedback about someone’s ability to do so using the Ask, Tell, Ask method.

Materials: This training game is played in pairs. It is played in two rounds. The seating for this training game are pairs of chairs are placed around the room giving the participants the room to spread out. The chairs have their back to each other. So when participants are seated they will have their backs to each other.

Time: Approximately 10 minutes.

Instructions:

Part One:

1. In the pairs one of the participants is assigned the letter name A and the other is assigned the letter name B. All the 'A's in this training game are given the following figure on a paper.

This image was created by Microsoft word, “Insert – Shapes” options. Please note, any shape is acceptable.

“Author owned.”

The 'B's in this training game are given an empty sheet of paper and a pen or a pencil. This is the first round.

1. The 'A's are expected to communicate to 'B' the figure in their hands so that the 'B's are able to draw an exact replica on the sheet given to them. In this round of the feedback game a constraint is introduced. The 'B's are told not to speak while playing this round of the training game. They have just to listen and draw according to the instructions by 'A'. Time limit of 60 seconds.
2. After everybody has finished playing the round the 'B's share their version of the figure with the 'A's. Very rarely does anybody get the figure right. There's quite a bit of laughter at the copies of the figure that the 'B's have made.
3. B’s Give feedback on A’s instructions – practicing the ask, tell, ask model. Share some examples:
   1. Example “Ask” questions: Examples: What went well? What was the easiest thing for you? What was the hardest part for you? What could have gone better?
   2. TELL: Acknowledge their response. Respond with an appropriate follow up. Examples: “I agree, it was helpful when you. (for ex. “used common objects as reference sizes”), But I had trouble understanding… (for ex. “the overall direction of the drawing”)
   3. ASK: If you got it right – does your feedback make sense to your learner? Do you agree? What will you change to incorporate what we have discussed?

Part 2: Switch positions. Repeat.

In the next round of this feedback game the 'A's are given a second figure.

“Author owned.”

This image was created by Microsoft word, “Insert – Shapes” options. Please note, any shape is acceptable.
