# Supplementary material for: Preparing Medical Students to Be Physician Leaders: A Leadership Training Program for Students Designed and Led by Students
Source: MedEdPORTAL. 2019 Dec 13;15:10863. doi: 10.15766/mep_2374-8265.10863 (PMC7012310; doi:10.15766/mep_2374-8265.10863)
Supplement: Supplementary file 1 — A. Session 1 PPT Leadership Styles.pptx B. Session 2 PPT Teamwork.pptx C. Session 3 PPT Delegation.pptx D. Session 4 PPT Feedback.pptx E. Session 5 PPT Direction.pptx F. Session 6 Optional Review PPT Consolidation.pptx G. Session 1 Activity Instructions.docx H. Session 2 Activity Instructions.docx I. Session 3 Activity Instructions.docx J. Session 4 Activity Instructions and Figure.docx K. Session 5 Activity Instructions.docx L. Session 6 Activity Instructions.docx M. Precourse and Postcourse Evaluation.docx N. Session 1 Evaluation.docx O. Session 2 Evaluation.docx P. Session 3 Evaluation.docx Q. Session 4 Evaluation.docx R. Session 5 Evaluation.docx S. Posttraining Evaluation.docx T. Supplemental Alternative Activity - PACE Palette.docx U. Supplemental Alternative Activity - ACLS Video.docx V. Supplemental Alternative Activity - Feedback Video.docx [file mep-15-10863-s001.zip › K. Session 5 Activity Instructions.docx]

Vision and Mission

*Activity to be performed following introduction to setting direction, refer to Appendix E: PowerPoint to Session 5, Direction*

Objective: Participants will practice organizational management through conceptualizing a shared vision and mission statement.

Materials: paper, pencil

Time: 15 – 20 min

Instructions:

- You are given a core value. As a team, develop a company with a new product promoting this core value, including a clear vision and mission statement.
- Consider: Who is your target audience? Where will you market your product? What is the cost? How will you achieve your mission and vision? How will you promote your value to employees? How will you promote your value to customers?
- To take it a step further: Develop a brand image/ slogan/ other marketing tool.
- Share your product and marketing design.

Group Discussion: Was it difficult to create a mission statement? Was it difficult to create a vision statement? What barriers did you run in to and have to overcome for marketing your product to employees? To customers? Did all team members agree in setting direction?

Examples of values:

- Going green/ environmental awareness
- Companionship
- Family fun
- Love
- Efficiency
- Speed
- Up to date technology / tech savvy
- Safety
