# Supplementary material for: Preparing Medical Students to Be Physician Leaders: A Leadership Training Program for Students Designed and Led by Students
Source: MedEdPORTAL. 2019 Dec 13;15:10863. doi: 10.15766/mep_2374-8265.10863 (PMC7012310; doi:10.15766/mep_2374-8265.10863)
Supplement: Supplementary file 1 — A. Session 1 PPT Leadership Styles.pptx B. Session 2 PPT Teamwork.pptx C. Session 3 PPT Delegation.pptx D. Session 4 PPT Feedback.pptx E. Session 5 PPT Direction.pptx F. Session 6 Optional Review PPT Consolidation.pptx G. Session 1 Activity Instructions.docx H. Session 2 Activity Instructions.docx I. Session 3 Activity Instructions.docx J. Session 4 Activity Instructions and Figure.docx K. Session 5 Activity Instructions.docx L. Session 6 Activity Instructions.docx M. Precourse and Postcourse Evaluation.docx N. Session 1 Evaluation.docx O. Session 2 Evaluation.docx P. Session 3 Evaluation.docx Q. Session 4 Evaluation.docx R. Session 5 Evaluation.docx S. Posttraining Evaluation.docx T. Supplemental Alternative Activity - PACE Palette.docx U. Supplemental Alternative Activity - ACLS Video.docx V. Supplemental Alternative Activity - Feedback Video.docx [file mep-15-10863-s001.zip › L. Session 6 Activity Instructions.docx]

Consolidation

*Please note: This is an optional enrichment activity and may be adapted as necessary to each course offering. Some of the questions on this score card may be specific to the individualized content offered by our presenters or by our optional supplemental material, as indicated with an asterisk, and therefore may not be generalizable for reproduction. We offer the model as a guide.*

Objective: Participate in a review of previous learning points, objectives, and skills, by completing the following tasks, working with a partner.

Materials: score card (below), questions (below), jelly beans, jar

Instructions: Set up 5 stations (desks/tables). Place a question at each station. With a partner, complete the following questions:

Questions:

Station 1

1. *UMMS has adopted the LEAN philosophy as a model of healthcare delivery. In this model, what are the three leadership behaviors that provide sustainable results?
   1. align <-> enable <-> improve
   2. manage <-> individual reward <-> efficiency
   3. enable <-> cut costs <-> values
2. Emotional intelligence consists of four fundamental capabilities. Each capability, in turn, is composed of a specific set of competencies. Match the capability with the correct competencies.
   1. self awareness: self control, adaptability
   2. self management: communication, building bonds
   3. social awareness: empathy, organizational awareness
   4. social skills: self confidence, accurate self assessment
3. Act it out! One of the six leadership styles is written on the back of this paper (coercive, authoritative, affiliative, democratic, pacesetting, coaching). Directions: ONE partner will turn this paper over and read the style (only one person should look). This person will then demonstrate the leadership style, acting it out (can use phrases, make up a scenario, examples). The other person will write down their guess on the score card.

Station 2

1. Arctic survival: You are in a plane crash and deserted in the arctic with certain materials which you must use for survival. Before the plane drifted away and sank, you were able to salvage the 8 items. **Your task is to rank these items according to their importance to your survival, starting with “1” the most important, to “8” the least important.** Record in column 1.
2. *Name two values of a “yellow” personality

Station 3

1. *Name two elements of effective team dynamics (ACLS video)
2. Discuss with your partner a situation in your future medical practice in which you foresee yourself practicing effective delegation skills.

Station 4

1. *Identify the correct feedback sandwich involving a providing effective feedback for a medical student/ patient interview.
   1. You have great rapport with patients. Your organization was confusing. You made great eye contact.
   2. You look nice today. Your interview was awful. You take concise notes.
   3. ASK- At which part of the interview did you feel comfortable and uncomfortable? TELL- I felt comfortable asking the cardinal 7, but nervous with the sexual history. ASK- Do you think being nervous affected if you were able to gather all the pertinent information? Let’s make a plan and follow up.
   4. ASK- How did that go for you? TELL- Ok. I was prepared but still nervous. TELL: Yes, I could tell you were nervous. You should work on that.
2. Name the four qualities of effective feedback.

Station 5

1. Estimate how many jelly beans are in the jar and write it on your score card in the first row labeled “Your guess.”
   1. Combine with another pair (that has not already combined with more than two pairs – groups should not exceed three pairs) and come to a consensus on the number of items in the jar. Write this answer on your paper.
   2. After two pairs have come to consensus, they are to find a third group (total: three pairs, one team of 6). This team is also to come to a consensus on the number of items in the container. Write this final consensus on your paper under “final team guess.” (NOTE: you may have to combine with a team that has already made two guesses, if this is the case, skip the second row and just fill in the final guess)
2. Choose the correct statement
   1. Vision: talks about the organizations present
   2. Mission: focus on “what is the benefit?”
   3. Vision: focus on “what do we do today?”
   4. Mission: focus on “what do we want to do going forward?”

| Items | 1  Your Ranking | 2  Expert Ranking | 3  Absolute Difference |
| --- | --- | --- | --- |
| A gallon can of maple syrup |  |  |  |
| A sleeping bag per person (arctic type down filled with liner) |  |  |  |
| A 20 x 20 piece of heavy duty canvas |  |  |  |
| 13 wood matches in a metal screw top, waterproof container |  |  |  |
| An operating 4 battery flashlight |  |  |  |
| 3 pairs of snowshoes |  |  |  |
| A fifth Bacardi rum (151 proof) |  |  |  |
| A hand axe |  |  |  |
| Total column 3 |  |  |  |

Score Card

Station 1

Q1. ________________

Q2. ________________

Q3. ________________

Station 2

Q2. ________________

________________

Station 3

Q1. ________________

________________

Station 4

Q1. ________________

Q2. ________________

________________

________________

________________

Station 5

Q1. Your guess________________

(Second team guess)_____________

Final guess________________

Q2. ________________

Total: __________/30

*Following the activity, please refer to Appendix F: Optional Review Session 6, Consolidation, to review answers and scoring*
