# Supplementary material for: Preparing Medical Students to Be Physician Leaders: A Leadership Training Program for Students Designed and Led by Students
Source: MedEdPORTAL. 2019 Dec 13;15:10863. doi: 10.15766/mep_2374-8265.10863 (PMC7012310; doi:10.15766/mep_2374-8265.10863)
Supplement: Supplementary file 1 — A. Session 1 PPT Leadership Styles.pptx B. Session 2 PPT Teamwork.pptx C. Session 3 PPT Delegation.pptx D. Session 4 PPT Feedback.pptx E. Session 5 PPT Direction.pptx F. Session 6 Optional Review PPT Consolidation.pptx G. Session 1 Activity Instructions.docx H. Session 2 Activity Instructions.docx I. Session 3 Activity Instructions.docx J. Session 4 Activity Instructions and Figure.docx K. Session 5 Activity Instructions.docx L. Session 6 Activity Instructions.docx M. Precourse and Postcourse Evaluation.docx N. Session 1 Evaluation.docx O. Session 2 Evaluation.docx P. Session 3 Evaluation.docx Q. Session 4 Evaluation.docx R. Session 5 Evaluation.docx S. Posttraining Evaluation.docx T. Supplemental Alternative Activity - PACE Palette.docx U. Supplemental Alternative Activity - ACLS Video.docx V. Supplemental Alternative Activity - Feedback Video.docx [file mep-15-10863-s001.zip › M. Precourse and Postcourse Evaluation.docx]

Pre Course Evaluation

1. Demographic Information

The purpose of this questionnaire is to gather demographic information. This data will provide information about our student population and will be used to help interpret course learning. Please answer all questions honestly. Your answers will remain anonymous. Thank you for your participation.

- Have you ever participated in formal leadership training/s?
  - Yes/ No
  - If yes – please list and briefly explain _______________________
- Have you ever held a leadership position?
  - Yes/ No
  - If yes, please list and explain briefly _______________________
- Why are you interested in this course? Please check all that apply
  - To learn more about effective leadership
  - I am interested in pursuing a leadership position during medical school
  - I am interested in a career that involves leadership
  - I struggle with leadership and hope to improve my skills
  - To enhance my residency application
  - Because my friends are enrolled
  - Other – please explain ___________________________

1. Baseline Information

The purpose of this questionnaire is to gather information of your baseline leadership knowledge and skills. Your responses will be used to help assess for learning throughout the course. Please answer all questions honestly. Your answers will remain anonymous. Thank you for your participation.

Directions: Please answer the following 10 questions by marking on the sliding scale (0 – 100) a numerical value indication of your confidence level in your ability in the following areas, ranging from not confident to very confident.

1. I identify my personal strengths as a leader

Not Confident Neutral Confidence Very Confident

0-------------------------------------------50------------------------------------------100

1. I identify personal areas that need improvement in order to be an effective leader

Not Confident Neutral Confidence Very Confident

0-------------------------------------------50------------------------------------------100

1. I communicate effectively with team members from different backgrounds

Not Confident Neutral Confidence Very Confident

0-------------------------------------------50------------------------------------------100

1. I identify situations in which team communication is effective, and situations in which it needs improvement

Not Confident Neutral Confidence Very Confident

0-------------------------------------------50------------------------------------------100

1. I delegate responsibilities appropriately when in a team setting

Not Confident Neutral Confidence Very Confident

0-------------------------------------------50------------------------------------------100

1. I recognize my limitation’s in a role and know when and how to ask for help as a responsible team member

Not Confident Neutral Confidence Very Confident

0-------------------------------------------50------------------------------------------100

1. I give appropriate and effective feedback to another person

Not Confident Neutral Confidence Very Confident

0-------------------------------------------50------------------------------------------100

1. I ask a preceptor for appropriate feedback in a professional manner

Not Confident Neutral Confidence Very Confident

0-------------------------------------------50------------------------------------------100

1. I develop an appropriate and successful strategy/plan when faced with a challenge or obstacle

Not Confident Neutral Confidence Very Confident

0-------------------------------------------50------------------------------------------100

1. I utilize my leadership skills to accomplish my future goals

Not Confident Neutral Confidence Very Confident

0-------------------------------------------50------------------------------------------100

Post Course Evaluation

Please answer the following questions.

- Please list the topic you found most useful. Briefly explain. (topics include leadership style, effective communication, delegation, feedback, management strategies)______________________________
- Please list a topic that you did not find useful. Briefly explain.________________________________
- Was any of the material surprising to you? Briefly explain.___________________________________
- Were there any topics that the course did not cover that you were hoping for? Briefly explain._______________________________________
- Were the topics covered by this course applicable to your future as a medical student?
  - Yes/ No
  - Please list examples of how this course is relevant to your medical school experience._____________________________________________
- Did you find that this course was applicable to your future medical career?
  - Yes/ No
  - Please list examples of how this course will apply to your medical career in the future.____________________________________________
- Did this course change your thinking about leadership skills and what it takes to be a good leader? Briefly explain.__________________________________________________________________
- Would you recommend this elective? Yes/ No
- Areas that I felt were done well (briefly explain):_______________________________________
- Areas that I felt needed improvement (briefly explain):__________________________________

Directions: Please answer the following 10 questions by marking on the sliding scale (0 – 100) a numerical value indication of your confidence level in your ability in the following areas, ranging from not confident to very confident. Thank you for your participation.

1. I identify my personal strengths as a leader

Not Confident Neutral Confidence Very Confident

0-------------------------------------------50------------------------------------------100

1. I identify personal areas that need improvement in order to be an effective leader

Not Confident Neutral Confidence Very Confident

0-------------------------------------------50------------------------------------------100

1. I communicate effectively with team members from different backgrounds

Not Confident Neutral Confidence Very Confident

0-------------------------------------------50------------------------------------------100

1. I identify situations in which team communication is effective, and situations in which it needs improvement

Not Confident Neutral Confidence Very Confident

0-------------------------------------------50------------------------------------------100

1. I delegate responsibilities appropriately when in a team setting

Not Confident Neutral Confidence Very Confident

0-------------------------------------------50------------------------------------------100

1. I recognize my limitation’s in a role and know when and how to ask for help as a responsible team member

Not Confident Neutral Confidence Very Confident

0-------------------------------------------50------------------------------------------100

1. I give appropriate and effective feedback to another person

Not Confident Neutral Confidence Very Confident

0-------------------------------------------50------------------------------------------100

1. I ask a preceptor for appropriate feedback in a professional manner

Not Confident Neutral Confidence Very Confident

0-------------------------------------------50------------------------------------------100

1. I develop an appropriate and successful strategy/plan when faced with a challenge or obstacle

Not Confident Neutral Confidence Very Confident

0-------------------------------------------50------------------------------------------100

1. I utilize my leadership skills to accomplish my future goals

Not Confident Neutral Confidence Very Confident

0-------------------------------------------50------------------------------------------100
