# Supplementary material for: Preparing Medical Students to Be Physician Leaders: A Leadership Training Program for Students Designed and Led by Students
Source: MedEdPORTAL. 2019 Dec 13;15:10863. doi: 10.15766/mep_2374-8265.10863 (PMC7012310; doi:10.15766/mep_2374-8265.10863)
Supplement: Supplementary file 1 — A. Session 1 PPT Leadership Styles.pptx B. Session 2 PPT Teamwork.pptx C. Session 3 PPT Delegation.pptx D. Session 4 PPT Feedback.pptx E. Session 5 PPT Direction.pptx F. Session 6 Optional Review PPT Consolidation.pptx G. Session 1 Activity Instructions.docx H. Session 2 Activity Instructions.docx I. Session 3 Activity Instructions.docx J. Session 4 Activity Instructions and Figure.docx K. Session 5 Activity Instructions.docx L. Session 6 Activity Instructions.docx M. Precourse and Postcourse Evaluation.docx N. Session 1 Evaluation.docx O. Session 2 Evaluation.docx P. Session 3 Evaluation.docx Q. Session 4 Evaluation.docx R. Session 5 Evaluation.docx S. Posttraining Evaluation.docx T. Supplemental Alternative Activity - PACE Palette.docx U. Supplemental Alternative Activity - ACLS Video.docx V. Supplemental Alternative Activity - Feedback Video.docx [file mep-15-10863-s001.zip › O. Session 2 Evaluation.docx]

Questionnaires for Session Two

Reflective Writing Question for Session Two

Outline:

5 point Likert scale (strongly disagree/disagree/neither disagree or agree/agree/strongly agree)

- Five questions specific to session material
- Ten questions specific to overall evaluation of session

Reflective writing question

Open-ended questions for Feedback Response

- Comments/ suggestions on what went well
- Comments/ suggestions on what could be improved
- General overall feedback and suggestions for future topics

Session Two Questionnaire:

1. Team Communication

|  | Strongly disagree | Disagree | Neither disagree or agree | Agree | Strongly agree |
| --- | --- | --- | --- | --- | --- |
| Utilizing collective intelligence is important for effective team management |  |  |  |  |  |
| I recognize the roles of others on a medical team and how they impact patient care |  |  |  |  |  |
| I am confident in my ability to actively listen to diverse points of view |  |  |  |  |  |
| I am confident in my ability to develop cooperative relationships among the people I work with |  |  |  |  |  |
| I am confident in my ability to communicate with others with dignity and respect |  |  |  |  |  |
| This session provided new information |  |  |  |  |  |
| This session was organized in a way that stimulated my learning |  |  |  |  |  |
| The material was relevant to me as a medical student and future physician |  |  |  |  |  |
| This session was worth the time that I invested |  |  |  |  |  |
| I found this session enjoyable |  |  |  |  |  |
| There was ample opportunity to ask questions |  |  |  |  |  |
| The amount of material covered was appropriate |  |  |  |  |  |
| The amount of participant involvement was appropriate |  |  |  |  |  |
| The instructors were well prepared and presented the content in a professional manner |  |  |  |  |  |
| Overall I am satisfied with this session |  |  |  |  |  |

1. Team Communication Learning Evaluation

Think about an experience in which you were on a team of people of various roles/backgrounds working toward a common goal (you can use today’s experience). Using the strategies that we have discussed today, reflect on worked well, or what did not work well and why. Provide one example of how you will use what you have learned from this experience in the future.___________________________________________________ __________________________________________________________________________________________

1. Please comment briefly on what areas of the session went well.______________________________________
2. Please comment briefly on what areas of the session need improvement.______________________________
3. Please comment on any other suggestions or concerns._____________________________________________
