# Supplementary material for: Preparing Medical Students to Be Physician Leaders: A Leadership Training Program for Students Designed and Led by Students
Source: MedEdPORTAL. 2019 Dec 13;15:10863. doi: 10.15766/mep_2374-8265.10863 (PMC7012310; doi:10.15766/mep_2374-8265.10863)
Supplement: Supplementary file 1 — A. Session 1 PPT Leadership Styles.pptx B. Session 2 PPT Teamwork.pptx C. Session 3 PPT Delegation.pptx D. Session 4 PPT Feedback.pptx E. Session 5 PPT Direction.pptx F. Session 6 Optional Review PPT Consolidation.pptx G. Session 1 Activity Instructions.docx H. Session 2 Activity Instructions.docx I. Session 3 Activity Instructions.docx J. Session 4 Activity Instructions and Figure.docx K. Session 5 Activity Instructions.docx L. Session 6 Activity Instructions.docx M. Precourse and Postcourse Evaluation.docx N. Session 1 Evaluation.docx O. Session 2 Evaluation.docx P. Session 3 Evaluation.docx Q. Session 4 Evaluation.docx R. Session 5 Evaluation.docx S. Posttraining Evaluation.docx T. Supplemental Alternative Activity - PACE Palette.docx U. Supplemental Alternative Activity - ACLS Video.docx V. Supplemental Alternative Activity - Feedback Video.docx [file mep-15-10863-s001.zip › P. Session 3 Evaluation.docx]

Questionnaire for Session Three

Reflective Writing Question for Session Three

Outline:

5 point Likert scale (strongly disagree/disagree/neither disagree or agree/agree/strongly agree)

- Five questions specific to session material
- Ten questions specific to overall evaluation of session

Reflective writing question

Open-ended questions for Feedback Response

- Comments/ suggestions on what went well
- Comments/ suggestions on what could be improved
- General overall feedback and suggestions for future topics

Session Three Questionnaire

1. Delegating Responsibilities

|  | Strongly disagree | Disagree | Neither disagree or agree | Agree | Strongly agree |
| --- | --- | --- | --- | --- | --- |
| I can utilize team dynamic management techniques when working with a team |  |  |  |  |  |
| Knowing how to delegate appropriate responsibilities is a necessary leadership skill to accomplish a complicated/intricate task |  |  |  |  |  |
| My ability to use effective team communication skills was improved from this session |  |  |  |  |  |
| I recognize my limitation’s in a role and know when and how to ask for help as a responsible team member |  |  |  |  |  |
| I am confident in my ability to delegate responsibilities as a team leader |  |  |  |  |  |
| This session provided new information |  |  |  |  |  |
| This session was organized in a way that stimulated my learning |  |  |  |  |  |
| The material was relevant to me as a medical student and future physician |  |  |  |  |  |
| This session was worth the time that I invested |  |  |  |  |  |
| I found this session enjoyable |  |  |  |  |  |
| There was ample opportunity to ask questions |  |  |  |  |  |
| The amount of material covered was appropriate |  |  |  |  |  |
| The amount of participant involvement was appropriate |  |  |  |  |  |
| The instructors were well prepared and presented the content in a professional manner |  |  |  |  |  |
| Overall I am satisfied with this session |  |  |  |  |  |

1. Delegating Responsibilities Learning Evaluation

Reflect on why it is important to delegate responsibilities on a healthcare team, using a clinical scenario. Provide one example of how this activity applies to your future work.____________________________________________

_____________________________________________________________________________________________

1. Please comment briefly on what areas of the session went well.______________________________________
2. Please comment briefly on what areas of the session need improvement.______________________________
3. Please comment on any other suggestions or concerns._____________________________________________
