# Supplementary material for: Preparing Medical Students to Be Physician Leaders: A Leadership Training Program for Students Designed and Led by Students
Source: MedEdPORTAL. 2019 Dec 13;15:10863. doi: 10.15766/mep_2374-8265.10863 (PMC7012310; doi:10.15766/mep_2374-8265.10863)
Supplement: Supplementary file 1 — A. Session 1 PPT Leadership Styles.pptx B. Session 2 PPT Teamwork.pptx C. Session 3 PPT Delegation.pptx D. Session 4 PPT Feedback.pptx E. Session 5 PPT Direction.pptx F. Session 6 Optional Review PPT Consolidation.pptx G. Session 1 Activity Instructions.docx H. Session 2 Activity Instructions.docx I. Session 3 Activity Instructions.docx J. Session 4 Activity Instructions and Figure.docx K. Session 5 Activity Instructions.docx L. Session 6 Activity Instructions.docx M. Precourse and Postcourse Evaluation.docx N. Session 1 Evaluation.docx O. Session 2 Evaluation.docx P. Session 3 Evaluation.docx Q. Session 4 Evaluation.docx R. Session 5 Evaluation.docx S. Posttraining Evaluation.docx T. Supplemental Alternative Activity - PACE Palette.docx U. Supplemental Alternative Activity - ACLS Video.docx V. Supplemental Alternative Activity - Feedback Video.docx [file mep-15-10863-s001.zip › Q. Session 4 Evaluation.docx]

Questionnaire for Session Four

Reflective Writing Question for Session Four

Outline:

5 point Likert scale (strongly disagree/disagree/neither disagree or agree/agree/strongly agree)

- Five questions specific to session material
- Ten questions specific to overall evaluation of session

Reflective writing question

Open-ended questions for Feedback Response

- Comments/ suggestions on what went well
- Comments/ suggestions on what could be improved
- General overall feedback and suggestions for future topics

1. Giving and Receiving Feedback

|  | Strongly disagree | Disagree | Neither disagree or agree | Agree | Strongly agree |
| --- | --- | --- | --- | --- | --- |
| I am prepared to give good positive feedback |  |  |  |  |  |
| I am prepared to give good negative feedback |  |  |  |  |  |
| Appropriate feedback influences the effectiveness of a medical team |  |  |  |  |  |
| I recognize when feedback is done well and when it is done poorly |  |  |  |  |  |
| I am confident to ask a preceptor for appropriate feedback |  |  |  |  |  |
| This session provided new information |  |  |  |  |  |
| This session was organized in a way that stimulated my learning |  |  |  |  |  |
| The material was relevant to me as a medical student and future physician |  |  |  |  |  |
| This session was worth the time that I invested |  |  |  |  |  |
| I found this session enjoyable |  |  |  |  |  |
| There was ample opportunity to ask questions |  |  |  |  |  |
| The amount of material covered was appropriate |  |  |  |  |  |
| The amount of participant involvement was appropriate |  |  |  |  |  |
| The instructors were well prepared and presented the content in a professional manner |  |  |  |  |  |
| Overall I am satisfied with this session |  |  |  |  |  |

1. Feedback Learning Evaluation

Reflect on a time in medical school in which you felt that feedback was given well, or poorly, and briefly describe why. Provide an example of how you will incorporate what you have learned in a future interaction.___________ _____________________________________________________________________________________________

1. Please comment briefly on what areas of the session went well.______________________________________
2. Please comment briefly on what areas of the session need improvement.______________________________
3. Please comment on any other suggestions or concerns._____________________________________________
