# Supplementary material for: Preparing Medical Students to Be Physician Leaders: A Leadership Training Program for Students Designed and Led by Students
Source: MedEdPORTAL. 2019 Dec 13;15:10863. doi: 10.15766/mep_2374-8265.10863 (PMC7012310; doi:10.15766/mep_2374-8265.10863)
Supplement: Supplementary file 1 — A. Session 1 PPT Leadership Styles.pptx B. Session 2 PPT Teamwork.pptx C. Session 3 PPT Delegation.pptx D. Session 4 PPT Feedback.pptx E. Session 5 PPT Direction.pptx F. Session 6 Optional Review PPT Consolidation.pptx G. Session 1 Activity Instructions.docx H. Session 2 Activity Instructions.docx I. Session 3 Activity Instructions.docx J. Session 4 Activity Instructions and Figure.docx K. Session 5 Activity Instructions.docx L. Session 6 Activity Instructions.docx M. Precourse and Postcourse Evaluation.docx N. Session 1 Evaluation.docx O. Session 2 Evaluation.docx P. Session 3 Evaluation.docx Q. Session 4 Evaluation.docx R. Session 5 Evaluation.docx S. Posttraining Evaluation.docx T. Supplemental Alternative Activity - PACE Palette.docx U. Supplemental Alternative Activity - ACLS Video.docx V. Supplemental Alternative Activity - Feedback Video.docx [file mep-15-10863-s001.zip › S. Posttraining Evaluation.docx]

Behavior Assessment

The purpose of this survey is to collect information regarding the applicability of the training.

Post Training Evaluation Online submission. To be distributed 6 months post course completion.

Since participating in the Leadership Elective:

1. Have you incorporated any components of your leadership style into your work?
   - Yes/ No,
   - Please briefly explain ____________________________________________
2. Have you utilized your skills in working w/ others to accomplish an identified goal or objective?
   - Yes/ No
   - Please briefly explain ____________________________________________
3. Have you utilized delegation skills to accomplish and balance your workload?
   - Yes/ No
   - Please briefly explain _____________________________________________
4. Since the training, have you given feedback to others?
   - Yes/ No
   - Please briefly explain _____________________________________________
5. Since the training, have you taken on a new leadership role?
   - Examples: student club leader, leader of free clinics, organized an event, run for a class position, summer work)
   - Yes/ No
   - Please briefly explain ______________________________________________
6. Do you feel confident in your skills as a leader and ability to utilize aspects of the training in a leadership role? (skills include using appropriate leadership style, effective communication, delegation, providing feedback, and management)
   - Yes/ No
   - Please briefly explain _______________________________________________
7. Do you aspire to a leadership position in the near future?
   - Yes/ No
   - If yes, have you identified a position? Please list:__________________________
8. Have you been able to share leadership knowledge with others?
   - Examples: Train others, write about your experience, recommend this elective
   - Yes/ No
   - Please briefly explain. _______________________________________________
9. Since you attended this elective, have you participated in other leadership related trainings?
   - Yes/ No
   - Please list _________________________________________________________

|  | I have not applied what I have learned | Somewhat applicable | Usually applicable | Applicable | I apply what I have learned regularly |
| --- | --- | --- | --- | --- | --- |
| 1. To what extent do you feel that what you have learned has been applicable in your daily life? |  |  |  |  |  |
