# Supplementary material for: Preparing Medical Students to Be Physician Leaders: A Leadership Training Program for Students Designed and Led by Students
Source: MedEdPORTAL. 2019 Dec 13;15:10863. doi: 10.15766/mep_2374-8265.10863 (PMC7012310; doi:10.15766/mep_2374-8265.10863)
Supplement: Supplementary file 1 — A. Session 1 PPT Leadership Styles.pptx B. Session 2 PPT Teamwork.pptx C. Session 3 PPT Delegation.pptx D. Session 4 PPT Feedback.pptx E. Session 5 PPT Direction.pptx F. Session 6 Optional Review PPT Consolidation.pptx G. Session 1 Activity Instructions.docx H. Session 2 Activity Instructions.docx I. Session 3 Activity Instructions.docx J. Session 4 Activity Instructions and Figure.docx K. Session 5 Activity Instructions.docx L. Session 6 Activity Instructions.docx M. Precourse and Postcourse Evaluation.docx N. Session 1 Evaluation.docx O. Session 2 Evaluation.docx P. Session 3 Evaluation.docx Q. Session 4 Evaluation.docx R. Session 5 Evaluation.docx S. Posttraining Evaluation.docx T. Supplemental Alternative Activity - PACE Palette.docx U. Supplemental Alternative Activity - ACLS Video.docx V. Supplemental Alternative Activity - Feedback Video.docx [file mep-15-10863-s001.zip › U. Supplemental Alternative Activity - ACLS Video.docx]

Session 3: Team Communication and Delegation

Optional Alternative Supplemental Teaching Tool – ACLS video on Team Dynamics

Instructions: view the video, and answer the video questions as a group, individually, or in partners

American Heart Association

eLearning

Course: ACLS Part 1

Optional Review Activity: Video, Team Dynamics; <https://elearning.heart.org/>

Must have course access; No copyrighted Permission.
